# Range and Frequency of Africanized Honey Bees in California (USA)

**DOI:** 10.1371/journal.pone.0137407

**Published:** 2015-09-11

**Authors:** Yoshiaki Kono, Joshua R. Kohn

**Affiliations:** Section of Ecology, Behavior and Evolution, Division of Biological Sciences, University of California San Diego, La Jolla, California, United States of America; CNRS, FRANCE

## Abstract

Africanized honey bees entered California in 1994 but few accounts of their northward expansion or their frequency relative to European honey bees have been published. We used mitochondrial markers and morphometric analyses to determine the prevalence of Africanized honeybees in San Diego County and their current northward progress in California west of the Sierra Nevada crest. The northernmost African mitotypes detected were approximately 40 km south of Sacramento in California’s central valley. In San Diego County, 65% of foraging honey bee workers carry African mitochondria and the estimated percentage of Africanized workers using morphological measurements is similar (61%). There was no correlation between mitotype and morphology in San Diego County suggesting Africanized bees result from bidirectional hybridization. Seventy percent of feral hives, but only 13% of managed hives, sampled in San Diego County carried the African mitotype indicating that a large fraction of foraging workers in both urban and rural San Diego County are feral. We also found a single nucleotide polymorphism at the DNA barcode locus COI that distinguishes European and African mitotypes. The utility of this marker was confirmed using 401 georeferenced honey bee sequences from the worldwide Barcode of Life Database. Future censuses can determine whether the current range of the Africanized form is stable, patterns of introgression at nuclear loci, and the environmental factors that may limit the northern range of the Africanized honey bee.

## Introduction

The honey bee subspecies *Apis mellifera scutellata*, was introduced from southern Africa to Brazil in 1956 in an effort to breed honey bees better suited to the Neotropics [1]. Swarms soon escaped containment and began to hybridize with European honey bees [2,3]. The resulting hybrid offspring are known as "Africanized" honey bees. Africanized bees have since spread throughout much of the Western hemisphere, and the advance of African genes both north and south from the introduction site has become one of the most-studied cases in the hybridization literature (reviewed in [4,5]). The Africanized honey bee reached Mexico by 1985, Texas by 1990 and California by 1994 [6,7]. Since 1994, the Africanized honey bee has continued to invade northward in California and east as far as Florida [8]. Africanized honey bees can exhibit highly aggressive behavior and at times attacks have resulted in human deaths [9]. Their invasion is of interest to evolutionary biologists as an example of the processes associated with hybridization, to apiarists whose hives may be taken over by more aggressive Africanized bees, and to the general public who may inadvertently disturb and be imperiled by feral or managed Africanized hives.

Many outcomes are possible when an invading population encounters a pre-existing one with which it can breed and produce fertile offspring. One form may completely displace the other because of some ecological advantage or strong selection against hybrids. Alternatively, the two forms may intermix opening the possibility that new genetic combinations could increase the fitness of the hybrid form in the invaded environment. Following the advance of Africanized honey bees over the past few decades, admixture, with the majority of nuclear genes and mitochondria coming from sub-Saharan Africa is the usual finding [5,10–15]. However, in some neotropical cases, near complete replacement of European by African mitotypes has been noted [16–17]. Despite persistence of some European genes, behaviorally and morphologically these admixed Africanized bees strongly resemble their sub-Saharan ancestor *Apis mellifera scutellata* [18–22].

Both morphological and genetic tests have been used to identify whether individual workers or groups of workers are of African or European origin. The simplest method to detect the presence of Africanized bees in a new world population is to restrict an amplified fragment of mtDNA to determine if the African (*Apis mellifera scutellata*) mitotype is present. Restriction of an amplified region of the mitochondrial cytochrome *b* gene with *Bgl*II discriminates bees carrying *Apis mellifera scutellata* mitochondria from those carrying mitochondria of European or Middle Eastern subspecies [23–24]. There are several additional methods to determine more precisely the region of origin of maternally inherited mtDNA by restriction or sequence analysis, but the cytochrome *b*—*Bgl*II assay remains the simplest and is highly reliable [24]. While mtDNA markers can distinguish whether the bee or bees in question are offspring of a maternal lineage carrying the mitochondria associated with *Apis mellifera scutellata*, microsatellite or other nuclear loci are needed to test the level of introgression in nuclear genes (see e.g. [14]).

There are also morphological differences between Africanized and European bees with Africanized workers tending to be smaller than their European counterparts. Using a few simple measurements and a discriminant function, one can determine with reasonable accuracy if bees are Africanized or European without the use of molecular tools [18,25]. Because body size and shape are likely to be partially or predominantly controlled by nuclear genes, morphological methods remain a cheap and efficient way to assess the extent of Africanization of honey bees in a sample, and to assess Africanization even when bees carry the European mitochondria, which may occur when Africanized drones mate with European queens.

While there have been many studies of the Africanization process in South and Central America as well as Texas, little research has been done in southern California since the initial invasion. The US department of agriculture maintains a database reporting the identification of swarms sent to the USDA ARS bee lab (Tucson, AZ). Africanized swarms have been reported as far north as Mariposa County in California’s central valley [7,26]. In addition to limited knowledge of their current range, little is known about the frequency of the Africanized bees in areas they have colonized.

Our study examines the current range of the Africanized honey bee in California as well as the frequency of Africanized honey bees in San Diego County by assaying foraging worker bees for mitotype and morphology. We also examine feral and managed honey bee colonies around San Diego County to determine whether the observed high frequency of the Africanized form is due to the predominance of feral bees or whether managed hives are commonly Africanized. Finally, we examine the linkage, if any, between polymorphism at the mitochondrial CO1 locus used for DNA barcoding, and previously used mtDNA polymorphisms for identifying Africanized bees. Use of DNA barcodes for mitochondrial origin of honey bee specimens would facilitate global monitoring of the spread of Africanized honey bees because honey bees are a common component of insect biodiversity samples collected for barcoding.

## Methods

No specific permissions were required for collecting activities as all sites were public roadsides and no sampling affected private property. No endangered species were sampled or harmed in this research.

### Bee Collections

Worker honey bees were collected in groups of three bees per site. Sites constituted any floral resource upon which honey bees workers were foraging. In urban areas these resources were often blooming horticultural plant species. In rural sites, native or naturalized plant species were the usual floral resource. No collections were made from crop plants. All three workers were usually collected within 10 meters of one another (max. 50 meters), often from the same flowering shrub, tree, or patch of groundcover. Collection of three bees per site allowed us to assess mitotype heterogeneity within foraging sites. North of San Diego County we collected bees throughout California west of the Sierra Nevada range at haphazardly chosen sites usually separated by 30 to 80 kilometers. Ninety-one sites were sampled extending as far north as Southern Oregon. In San Diego County we collected bees from 88 sites usually separated by approximately 8 kilometers. All bees were collected using hand held insect nets, killed in soapy water (one drop of dish soap in 50ml H_2_0), immediately rinsed in fresh water and then pinned to dry or placed in 95% EtOH and pinned to dry within 24–48 hrs. Coordinates and other Information on the collection sites for all specimens can be found in S1a and S1b Table.

We also obtained samples of ten workers from each of 23 beekeeper-managed hives in San Diego County. Hive bees came from 11 different beekeepers who represented a mixture of hobbyists and honey bee professionals. For these samples we asked beekeepers if the hive was started by ordering a colony from a commercial source or by capture of a feral swarm, and the time since last requeening of the hive. Commercial colonies are unlikely to be Africanized but swarms could arise from feral colonies and be Africanized. Our beekeepers only requeened with commercially reared European queens and thus requeening also increases the probability that colonies consist of European bees. Worker bees from managed hives were killed by freezing at -5°C, air dried, and shipped to our lab. One sample colony arrived with severe mold and was not used for morphological analysis although the mitotype of one bee was determined (see methods below). Using nets, we also obtained ten bees each from the nest entrances of ten feral colonies located in San Diego County.

### Morphological measurements

We followed the methods outlined in [25] to differentiate Africanized from non-Africanized honey bee workers based on morphology. Measurements taken were lengths of the right forewing, right hindwing, and the lengths of the tibia and femur of the right hind leg (character set 2 of [25]). Bee parts were mounted on slides and images were captured using IScapture and measured using imageJ software [27]. For bees sampled at foraging sites, the factor loadings for individual worker bees from Table 6 of [25] were applied (2.5164*forewing length + 1.2159*hindwing length + 16.3439*femur length– 10.6356*tibia length– 36.4909). For the groups of ten bees sampled from each managed or feral colony, means of each measurement were taken, and the factor loadings for colony means recommended in Table 3 of [25] were used (2.7535*forewing length + 2.6834*hindwing length + 27.9261*femur length– 19.5551*tibia length– 46.5884).

### Molecular analysis

The left hind legs of dried bees were crushed under liquid nitrogen, and DNA was extracted using the Qiagen DNeasy blood and tissue kit according to the manufacturer’s protocol. To assess if bees had mtDNA characteristic of sub-Saharan Africa, we amplified a region of the mitochondrial cytochrome *b* gene in 25 μl reactions using primers and conditions described in [24]. After amplification, 10 μl of the PCR product was digested with *Bgl*II at 56°C for 3 hours [24]. One band present after digestion and electrophoresis indicated the specimen had African mtDNA, while a two-banded phenotype was scored as European mtDNA [24,28].

To dissect further the origins of San Diego County honey bees, we amplified and sequenced the cytochrome I (COI)–cytochrome II (COII) intergenic spacer from a random subset (N = 48) of honey bee foraging workers using primers E2 and H2 of [29]. The PCR conditions were: denaturation at 94°C for 2 min, followed by 35 cycles of 94°C for 45s, 46°C for 45s, and 72°C for 45s, and then a final extension of 72°C for 5 min. PCR products were visualized on a 2% agar gel and sequenced by Retrogen Inc., San Diego, CA. The DNA barcode region of the mtDNA COI gene was also amplified from these same 48 bees in order to determine associations between COI variation and other markers. Amplifications used the protocol and the primers LCO1490 and HCO298 described in [30]. Sequence alignment and analyses were performed using the MEGA 6 software package [31].

## Results

### Restriction-site analysis

**Fig 1:**
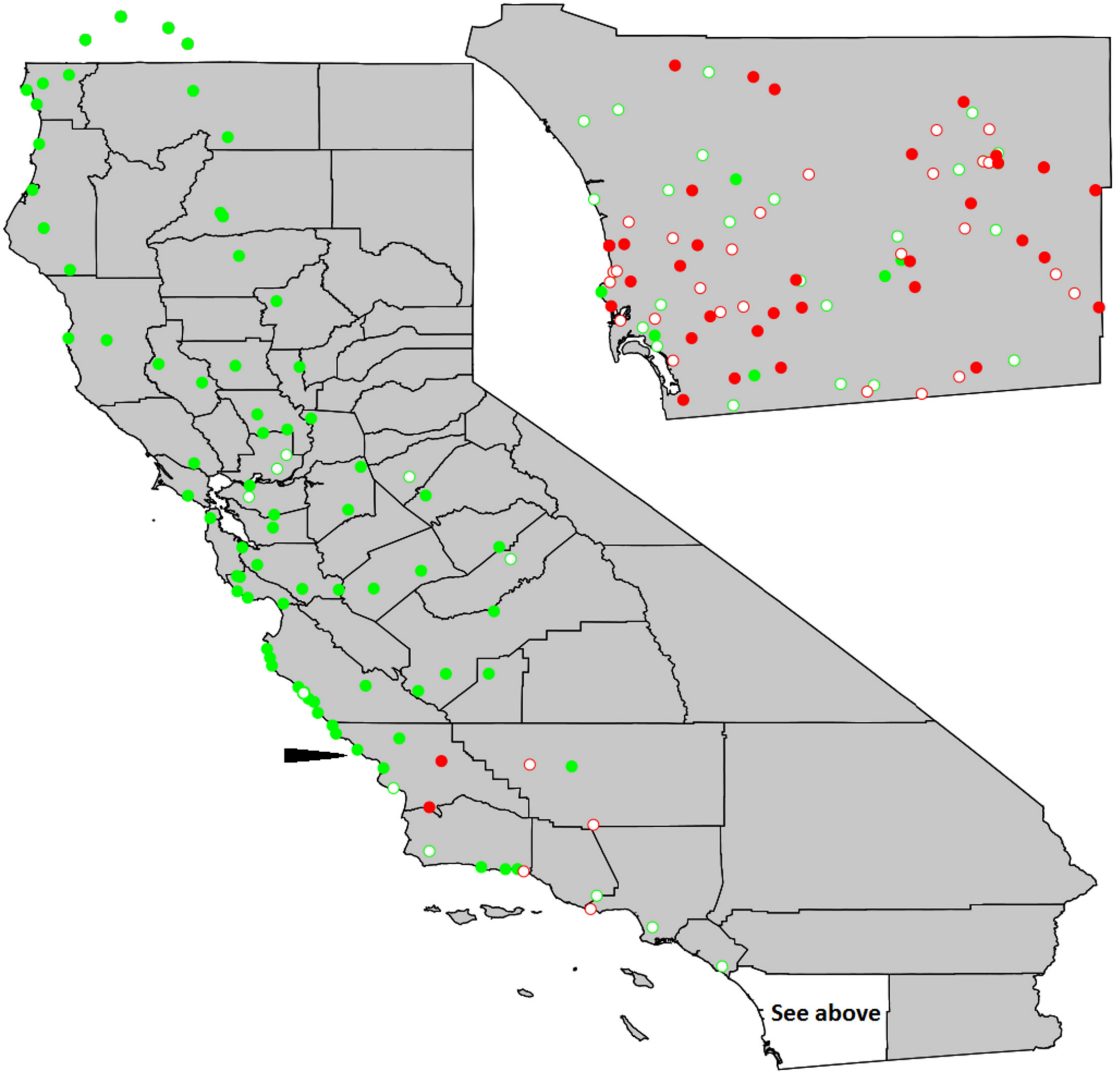
Map of California showing proportions of *Apis mellifera* with African vs. European mitochondria at each site. For coordinates of collection sites and number of worker bees sampled at each site (usually three) see S1 Table. Inset is sampling from San Diego County. The arrow indicates 35.5°N. Filled green, all bees European mitotype; unfilled green, two European, one African; unfilled red, two African, one European; filled red, all African.

North of San Diego County we performed the cytochrome *b*—*Bgl*II restriction analysis on 265 bees collected at 91 sites (Fig 1). Twenty-three bees (8.7%), carried the African mitochondria. South of 35.5°N latitude, bees with the African mitochondria were fairly common. North of that latitude, we found African mitochondria in only 6 of 233 bees. These occurred in scattered locations, one near the Pacific coast at the University of California Big Creek Reserve (36.074014, -121.59418), the others either in the foothills of the Sierra Nevada mountains, the Central Valley, or the easternmost regions of the San Francisco Bay Area. The northernmost African mitochondria was detected approximately 40 km south of Sacramento (38.1838840, -121.8433948; S1a Table).

In contrast to the statewide sample, honey bees throughout San Diego County were highly Africanized. Restriction analysis revealed that 64.8% of the 298 bees from San Diego County carried the African mitochondrion. There was no readily apparent spatial pattern to the where Africanized honey bees were collected, with bees carrying the African mitotype found in all habitats (scrub, chaparral, mountain forests, deserts) and at all levels of urbanization from the urban core in coastal regions to undeveloped areas in the eastern parts of the county. In addition, considerable heterogeneity in mitotype occurred at the level of the collecting site. Of the 95 sites from which three bees were collected, 55 contained workers with alternative cytochrome b–*Bgl*II restriction phenotypes indicating the presence of workers from different hives attending the same floral resource. The frequencies of sites with 0, 1, 2, or 3 bees with African mitochondria were not significantly different than expected by chance when drawing three bees at random from the observed mitotype frequencies (*χ* ^2^ = 7.0, DF = 3, NS).

### Morphological analysis

**Fig 2:**
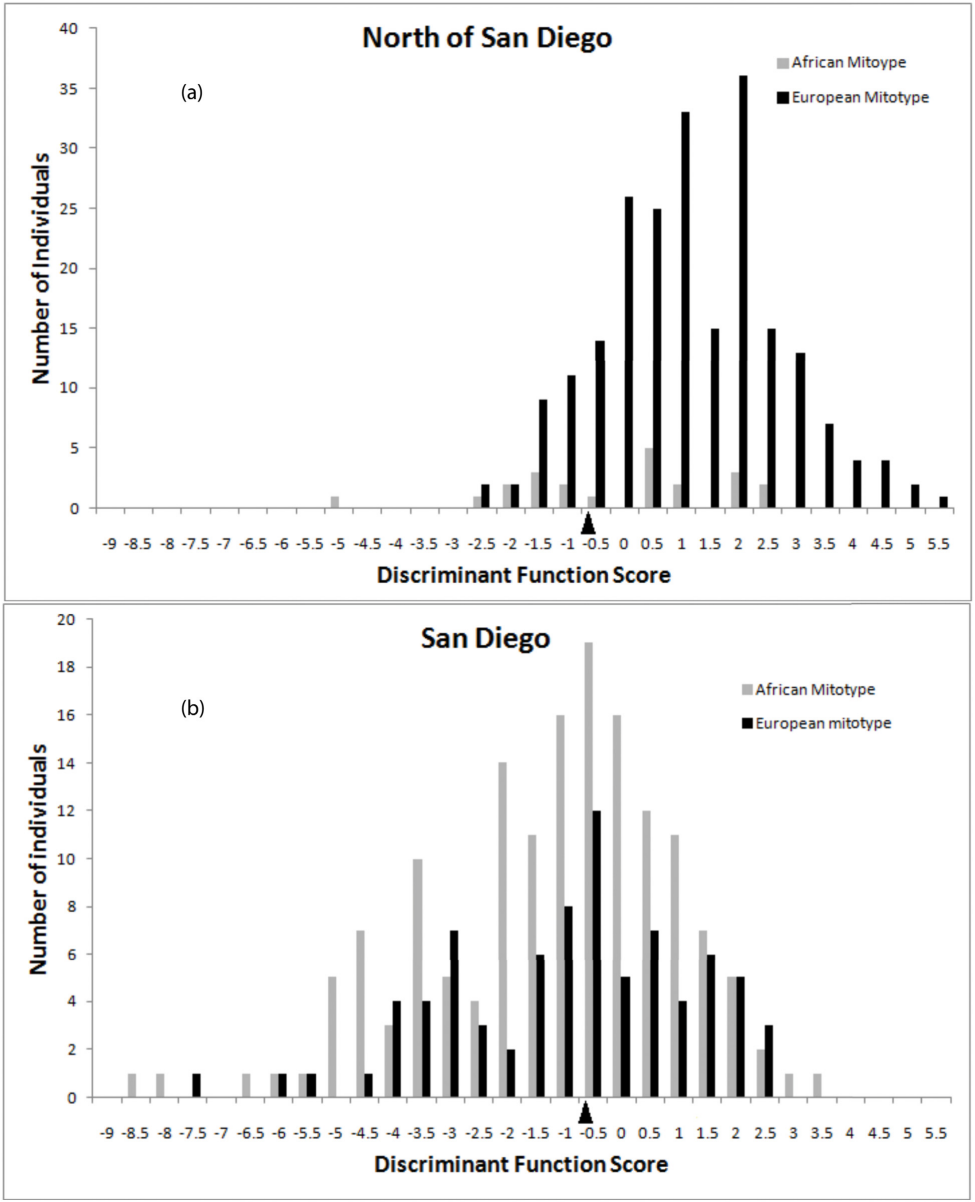
Distribution of morphological discriminant function scores for individual workers carrying European or African mitochondria sampled a) north of San Diego County and b) within San Diego County. Arrow indicates the midpoint between the mean discriminant function scores for individual European and Africanized workers [25]. Bees with scores smaller than the midpoint are classified by the discriminant function as Africanized while bees with scores larger than the midpoint are classified European. The expected misclassification rate of this discriminant function for individual workers is 9.9% [25].

Using the discriminant function for individual workers, 17.84% of the honey bees north of San Diego County were scored as African (Fig 2a). Honey bees with the African mitotype had a mean discriminant function score of -0.428, whereas those with the European mitotype had a significantly larger mean value (0.902, *t* = 14.3, DF = 239, P < 0.001; S2a Table). Within San Diego County, 60.85% of honey bees were morphologically classified as African (Fig 2b), a percentage similar to that found based on mtDNA. However, the mean discriminant function score for honey bees with the African mitotype (-1.473), did not differ from that of bees carrying the European mitotype (-1.180; *t* = 0.99, DF = 232, NS; S2b Table).

### Bees from San Diego County hives

Of the 24 managed hives sampled in San Diego County, 3 (12.5%) carried the African mitotype, a significantly lower frequency than found among workers collected at floral resources (*χ* ^2^ = 29.07692, DF = 1, P < 0.005). The three hives that contained bees with the African mitotype all originated from feral swarms brought into domestication. Of the ten feral colonies assessed, seven (70%) carried the African mitotype, a frequency not different than that found among foraging workers county-wide (*χ* ^2^ = 0.10989, DF = 1, NS; S3a Table).

**Fig 3:**
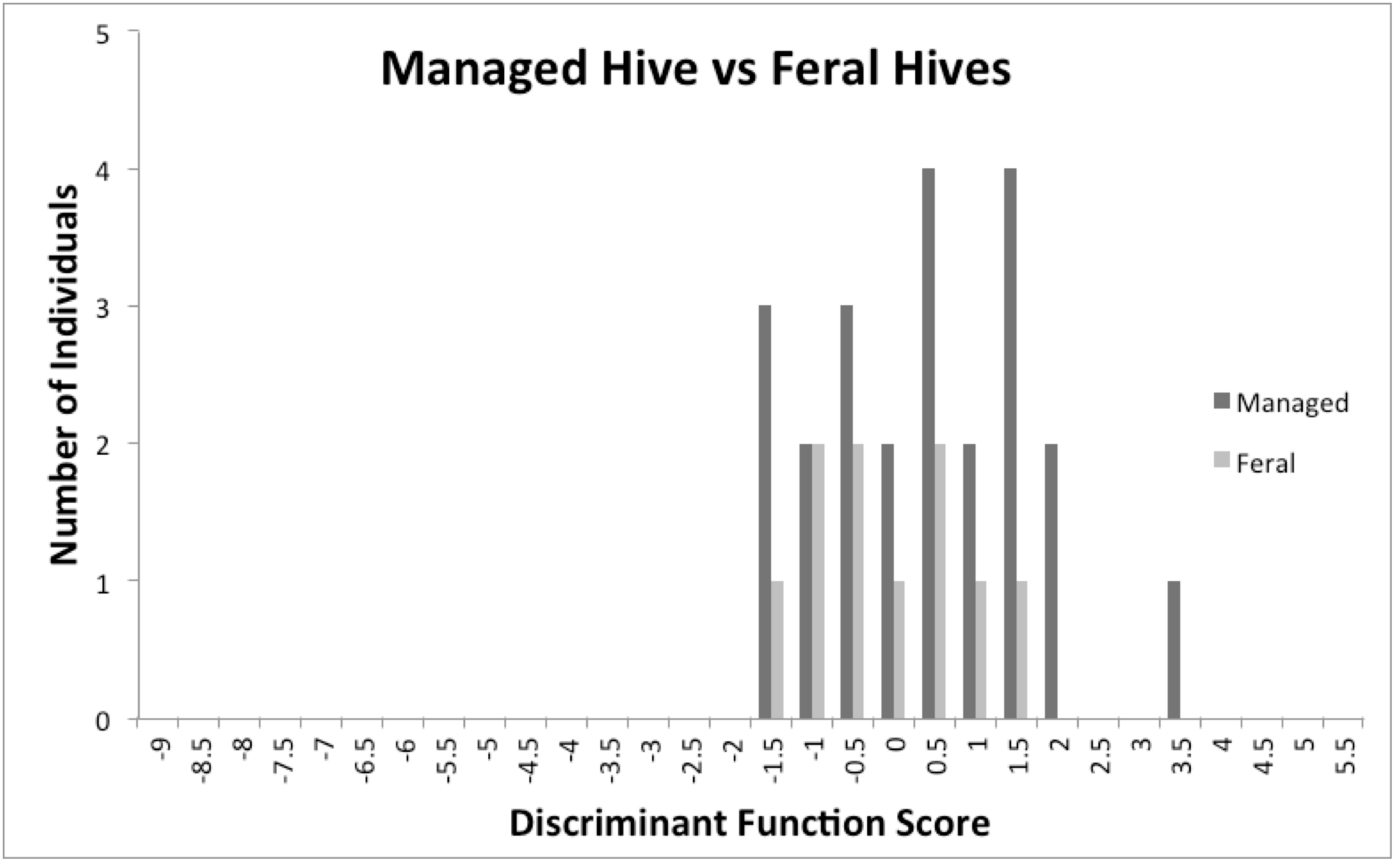
Distribution of morphological discriminant function scores for means of ten workers per colony collected from feral and domestic hives in San Diego County. Arrow indicates the midpoint between the mean discriminant function scores for worker mean measurements from European and Africanized colonies [25]. Colonies with scores smaller than the midpoint are classified by the discriminant function as Africanized while those with scores larger than the midpoint are classified European. The expected misclassification rate of the discriminant function for worker means is 3.5% [25].

Morphologically, 5 of 23 honey bee colonies from local beekeepers provided discriminant function scores indicating Africanization (Fig 3). Two of the three hives carrying the African mitotype scored as African by morphological analysis as well as a third hive known to have been started from a feral swarm but which carried the European mitotype. Of the remaining 20 hives, the fact that two (10%) provided discriminant scores identifying them as African may reflect some further Africanization, but is not significantly different than the expected misclassification rate (3.5%; [25]) of the discriminant function for colony means (*χ* ^2^ = 2.50, DF = 1, NS). The mean discriminant function score of colony workers from managed hives in which the queen carried the African mitochondria was -1.082 while that for hives carrying the European mitochondria was larger 0.2965 (*t* = 1.7915, DF = 21, P < 0.0438). Workers from feral colonies were surprisingly large given the high frequency of African mitotypes found within them. Only 3 of the 10 colonies scored as African using discriminant function of worker means (Fig 3), and there was no difference in the mean discriminant scores of feral hives carrying the African ($\bar{x}$ = -0.3530, N = 7) versus the European ($\bar{x}$ = -0.3526, n = 3) mitotypes (*t* = 0.0006, DF = 8, NS; S3b Table).

### Additional sequence analyses

For a random sample of 48 workers collected at floral resources in San Diego County we sequenced the COI-COII intergenic region (Genbank accessions KT279822-KT279869) previously used to distinguish within and among African and European mitotypes (see e.g. [24,32]) and the mitochondrial COI DNA barcode locus (Genbank accessions KT275960-KT276007). Using phylogenetic analysis (S1 Fig), the 19 bees carrying mitotypes assessed as European by the cytochrome *b*—*Bgl*II assay were found to represent a mixture of C, O, and M mitotypes (Table 1; S1 Fig; S4 Table) which are lineages from the northern Mediterranean, Middle East, and western Europe, respectively [32,33]. All 29 mitotypes classified as African by restriction analysis were found by sequencing the COI-COII intergenic spacer to represent the A mitotype characteristic of sub-Saharan Africa. Therefore perfect linkage between the cytochrome *b* restriction site and mitotypes defined using COI-COII intergenic sequences was confirmed [24].

**Table 1:** Linkage between mitotype sequence and restriction site variation in 48 randomly sampled honey bee workers from San Diego County. For cytochrome *b*, numbers denote one- (African) and two- (European) banded phenotypes after digestion with *Bgl*II. For CO1, numbers denote sequences that have a cytosine versus a thymine at position 2382. For COI—COII intergenic spacer sequences, numbers denote the frequency of previously described mitochondrial types (A = Africa, C = Carniola/Slovenia, M = Northern and Western Europe, and O = Oriental/Middle Eastern) determined by phylogenetic analysis (see S1 Fig).

	RFLP (Cytb)	COI	COI—COII Spacer
**African**	29	29	A—29
**European**	19	19	C—5
			O—8
			M—6
**Totals**	48	48	48

**Fig 4:**
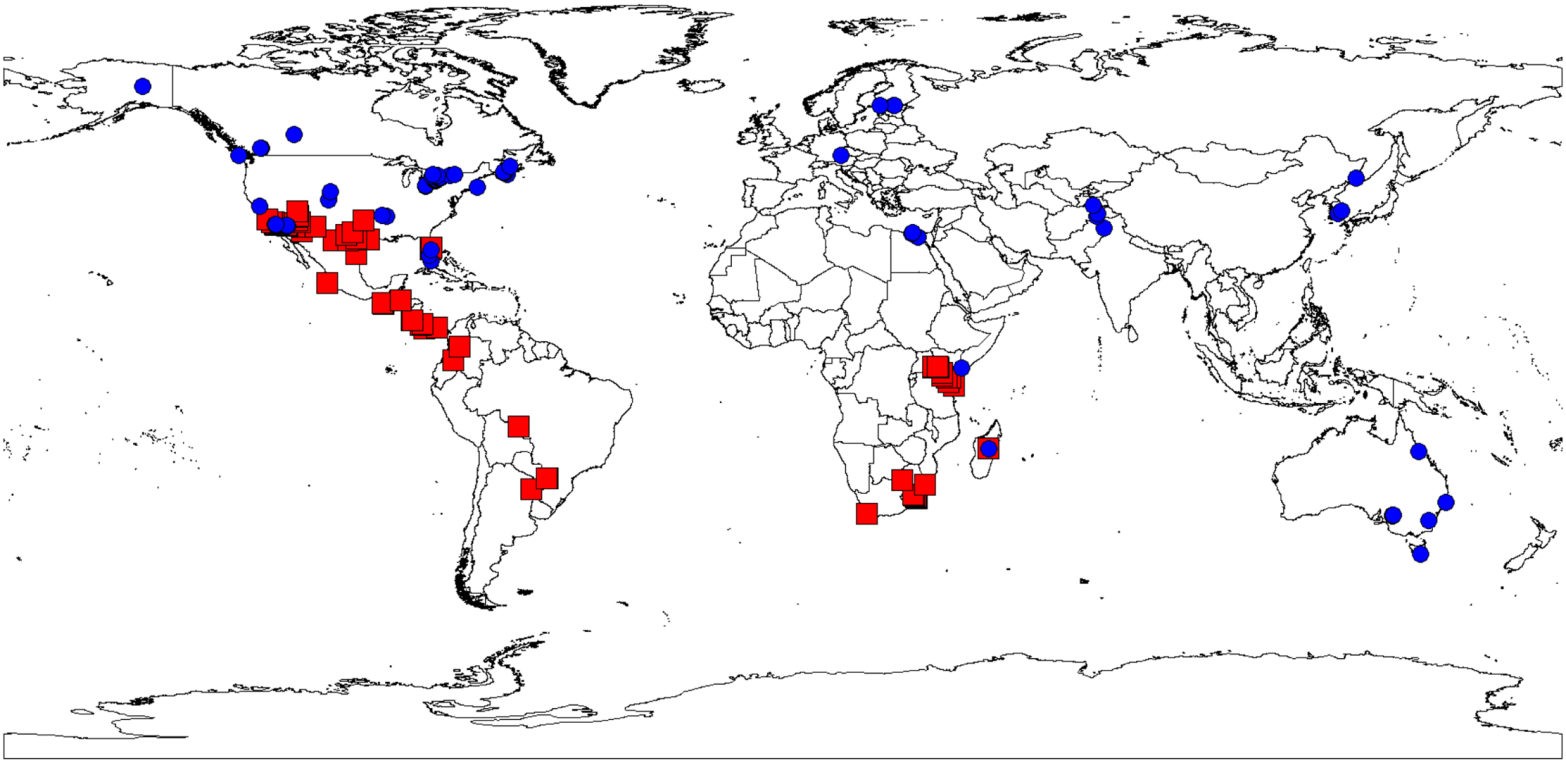
Worldwide distribution of *Apis mellifera* mitotypes based on a single nucleotide polymorphism at position 2382 [32] of the *Apis mellifera* mitochondrial genome. 401 high-quality *Apis mellifera* sequences were downloaded from the Barcode of Life Database (Barcode Index Number BOLD:AAA2326, S5 Table). Red: locations of individuals with a cytosine at position 2382. Blue: locations of individuals with a thymine at that position (see text).

At the DNA barcode region of the mitochondrial COI gene, we found a single nucleotide polymorphism (SNP) at position 2382 as defined in [34] that was in perfectly correlated with the cytochrome *b*—*Bgl*II restriction pattern (Table 1, Genbank accessions KT275960-6007). A cytosine at that position was found in all bees that gave the restriction pattern characteristic of the African mitotype, while a thymine was found at that site for all bees classified by restriction as European. To test whether this SNP is diagnostic on a wider sample of honey bees, we downloaded 401 high-quality *Apis mellifera* sequences (BIN AAA2326, S5 Table) with locality information from the Barcode of Life Database (http://www.boldsystems.org/) the worldwide repository of DNA barcode data [35]. The SNP phenotype diagnostic of the African mitochondrion was restricted to samples from sub-Saharan Africa, the neotropics, and the southern US (Fig 4; samples from South Africa, Argentina, Madagascar, Bolivia, Ghana, Kenya, Uganda, Colombia, Costa Rica, Ecuador, Panama, Mexico, Honduras, Texas, Florida, Arizona, New Mexico, California, and Oklahoma). The SNP phenotype diagnostic of European mitotypes was found in samples from Australia, Alaska, Alberta, Arizona, Arkansas, Bavaria, British Columbia, California, Colorado, Oklahoma, Florida, Tajikistan, Pakistan, Massachusetts, Nova Scotia, Ontario, Russia, Madagascar, Kenya, Egypt, South Korea, Vietnam, and Finland. Detection of some European honey bees in Africa is expected if managed hives are present. However, no mitotypes carrying a cytosine at position 2382 were reported outside of the currently known range of African and Africanized bees indicating this SNP is diagnostic of African versus non-African mitochondrial origin across a broad sample.

## Discussion

Since 1994, Africanized honey bees have expanded their range in California from the two counties bordering Mexico to the northern reaches of the Sacramento River delta. The northernmost site at which we recorded the presence of the African mitotype is approximately 250 kilometers northwest of the previously reported northernmost limit of Africanized honey bees in Mariposa County [7,26]. Africanized honey bees are thought to be capable of expanding their range by as much as 300–500 kilometers per year [6]. Since Africanized honey bees were recorded in Mariposa County in 2006, it would appear that range expansion has slowed considerably from maximal rates. More detailed sampling in subsequent years will be needed to determine both the exact range limit and whether it is stable.

Africanized bees appear to have a reduced ability to survive cold winter temperatures and two attempts at predicting the eventual northern limit of the Africanized honey bee have been made. One study [36] used data from the southern New World limit of the range of Africanized honey bees to predict that the northern limit would be set where the average temperature was below 16°C during the coolest month (January). Harrison et al. [26] noted that by 2006 Africanized honey bees were already found overwintering north of this prediction and our study found them even further north. However, as the climate warms, the predicted range limit will also shift north so expansion beyond the predicted limit might be explained by recent warming. Our sampling occurred following the warmest winter on record for California (http://www.ncdc.noaa.gov/). If winter temperatures limit the range of Africanized honey bees, the northern range limit may covary to some degree with fluctuations in winter temperatures. Southwick et al. [37] used physiological measurements of energy balance to predict Africanized honey bees would be limited by the isocline of 120 consecutive days not exceeding 10°C during winter. Under this prediction, Africanized honey bees were expected to extend their range at least to the border with Canada along the mild west coast of the United States. This has clearly not yet occurred.

Our data on further range extension should be treated with caution because we surveyed only summer foraging workers and therefore cannot determine whether Africanized bees were successfully overwintering in all areas in which they were detected. Studies of overwintering of feral hives are needed to determine this. In addition, our data on the frequency of Africanized honey bees needs to be tempered by the fact that our state-wide survey assayed only freely foraging workers, not swarms or feral hives. Although we assayed workers after nearly all crops had finished flowering and many imported commercial honey bee hives had presumably left the state, domestic hives were still frequently observed in agricultural regions such as the Central Valley. Therefore we cannot assess the frequency with which feral bees are Africanized in our statewide sample, only that among workers encountered while foraging, Africanized bees are currently the clear minority north of approximately 35.5 degrees of latitude.

North of San Diego County honey bees carrying the African mitotype were smaller on average than those carrying European mitotypes. This difference is expected for early stages of introgression, though such associations may subsequently disappear as linkage between mitotype and nuclear gene content breaks down with further introgression [5,38]. In addition, worker bees in the statewide sample were on average larger than those collected in San Diego (Fig 2a and 2b). This may be due partly to the more recent arrival and lower frequency of Africanized honey bees in northern regions, but another factor is that feral European honey bees in California exhibited Bergmann’s Rule (larger body size with higher latitude) prior to the invasion of Africanized honey bees [39].

In San Diego County, the African mitotype was found in nearly 65% of foraging workers and 61% of workers were classified as Africanized by morphological analysis, but there was no association between body size and mitotype. Assuming that genetic determinants of body size are at least partly controlled by nuclear genes, it appears that the process of Africanization in San Diego has likely followed the pattern previously documented in Texas [5] where initial linkage between mitotype and nuclear gene content broke down over a period of less than decade following invasion. What remained was a feral population of bidirectionally hybridized bees in which the frequencies of European nuclear genes and mitotypes averaged 25–37% with no linkage disequilibrium between genes from maternal and biparental genomes (Pinto et al. 2005). Our observed frequencies of European mitotypes and morphologically European bees are at, or slightly in excess of, the higher limit of the range documented in Texas [5]. However, in that study [5] only bees from feral colonies were studied while our collecting strategy focused on workers that may have come from either domestic or feral sources, potentially explaining the slightly higher frequency of European mitotypes and morphologies in our sample. Assays of nuclear gene content are needed, but it seems likely that rather than considering > 60% of honey bees in San Diego as Africanized, the actual situation is that most worker bees in the county represent a hybrid population containing > 60% African genes.

Our sample indicates that the African mitotype remains uncommon in domesticated hives in San Diego County, except for the few cases where hives were founded from feral swarms. By contrast the frequency of the African mitotype in the ten feral hives sampled (70%) was not different than that found among freely foraging workers in San Diego County. Because 21 of 24 (87.5%) domestic hives but, only 35% of foraging workers, carried the European mitotype, it appears that the great majority of foraging worker honey bees encountered in San Diego County do not come from domestic hives, but rather represent a large feral honey bee population. In addition, we observed heterogeneity of mitotypes among the three workers collected at each site as often as would be expected by randomly sampling the entire population. Therefore patches of flowers are often visited by workers from more than one hive. Honey bees can forage as much as 10 kilometers from their hive [40], but in our sampling, the floral resource from which bees were collected were common on the landscape, consisting of either frequently planted horticultural varieties in urban landscapes or common native species in rural ones. When floral resources are common, median foraging distances of honey bees may be considerably shorter than the maximum. For instance, a median foraging distance of 1.1 kilometers was estimated by analysis of waggle dances in a study area in Germany [40]. In a study of foraging distances of honey bee from hives on the UC San Diego campus, the median distance was 0.929 kilometers (Park, B. and Nieh, J. unpublished data). If foraging distances are similar throughout San Diego County, the distances between feral hives may often be less than 2 kilometers.

Our most puzzling data come from measurements of workers from feral hives. Only three of ten colonies were scored as Africanized by the discriminant function method [25], and bees from feral hives did not differ morphologically from bees from managed hives even though the managed colonies much more frequently carried European mitotypes. Our best explanation is that this result may be due simply to the small sample size (N = 10), although even with this small sample, finding that only 3 (30%) colonies that scored as Africanized rather than the expected 60.85%, as found in our large sample of San Diego County foraging workers is marginally significant (*χ* ^2^ = 3.995, P = 0.046).

Sequencing of the COI—COII spacer region confirmed that the cytochrome *b*–*Bgl*II restriction method reliably separates the “A” (African) mitotype from several mitochondrial lineages that are found in Europe and the Middle East. It should be noted that Franck et al. [32] found that the A mitotype occurs north of the Sahara in western North Africa, Sicily and the Iberian peninsula. Therefore, honey bees with ancestry from these sources could be detected as carrying the African mitochondrial lineage and might account for the occasional report of “African” mitochondria that pre-date the recognized arrival of Africanized honey bees from Central and South America. For instance, in a survey of 451 feral honey bee colonies collected across the southern United States (excluding California) prior to the recognized arrival of Africanized honey bees, one colony, collected in 1980 from Georgia carried the African mitotype [25,28]. This finding suggests some previous introduction, perhaps through port traffic. If such prior introductions succeeded in California, their offspring appear to be rare or absent in the northern parts of California and the pattern of occurrence of the “A” (African) mitotype strongly suggests little if any presence in California prior to the invasion of Africanized bees in 1994.

Our investigation of the DNA barcode region of the mitochondrial COI gene provides a new method to distinguish between African and European mitotypes. This region contains many other polymorphisms, and could potentially be used to distinguish further among African and European subtypes (see e.g. [8,32]). However the most important utility of this diagnostic SNP will be for further documentation of the spread and frequency of Africanized honey bees. DNA barcoding is becoming an increasingly popular method of assessing biodiversity used both in research and classroom contexts. Because honey bees are frequently collected during invertebrate biodiversity assessments that utilize DNA barcoding, the Barcode of Life Database will provide ongoing data on further spread of Africanized honey bees.

The pattern of Africanization we have documented in San Diego County and elsewhere in California appears consistent with patterns previously documented in Texas where Africanized honey bees first appeared in the United States [13]. In San Diego County, Africanized honey bees were first reported in 1994 and there now appears to be an admixture of European and African genes with the African component in the majority and no linkage between mitochondrial and morphological assessments of Africanization. Of great interest for further study would be complete assessment of nuclear genomic origins using molecular tools, [41], particularly whether there is a pattern as to which European and African nuclear genes persist after hybridization stabilizes across the geographic range of Africanized honey bee expansion. Secondly, we have not assessed aggressive behavior in Africanized honey bees in California. Doing so requires assessment of colony-level aggression as it is at this level that Africanized and European honey bees strongly differ behaviorally. Reports from the island of Puerto Rico suggest that Africanized bees there are less aggressive than elsewhere, perhaps due to limited penetration into the Africanized population of certain regions of the African nuclear genome [42,43]. Behavioral studies of Africanized honey bees at the northern limits of their continental range are lacking. It is thought that aggression or its behavioral correlates is a part of the ecological advantages that allow Africanized honey bees to displace European honey bees [4,6]. If so, we expect genes that control this behavior to persist in hybridized populations wherever aggression is advantageous. Conversely, whether certain European genes provide advantages to hybrids over pure African genomes remains to be studied.

## Supporting Information

S1 Table Honey bee collection sites north of San Diego County (S1a) and within San Diego County (S1b). The number of honey bees, date collected, number carrying African mitochondria, location and elevation of collecting sites north of San Diego County.
(DOCX)

S2 Table Morphometric measurements, discriminant function scores and outcomes, and cytochrome b restriction results for honey bees collected north of San Diego County (S2a) and within San Diego County (S2b): FWL, fore wing length; HWL, hind wing lenghth; TL, tibia length; FL, femur length (all in mm). (DOCX)

S3 Table Morphometric measurements, discriminant function scores and outcomes, and cytochrome b restriction results for honey bees collected from hives of 11 San Diego beekeepers (S3a) and from 10 feral hives within San Diego County (S3b): FWL, fore wing length; HWL, hind wing lenghth; TL, tibia length; FL, femur length (all in mm). (DOCX)

S4 Table Genbank accession numbers for honey bee mitochondrial COI—COII spacer region sequences used in S1 Fig. (DOCX)

S5 Table Collection information from 402 high quality sequences of honey bees (*Apis mellifera*) from the Barcode of Life Database (barcode index number (BIN) AAA2326). Honey bee sequences scored as African have a cytosine at position 2382 (Crozier and Crozier 1993) within the COI gene, all others have a thymine at that position.
(DOCX)

S1 Fig Maximum likelihood consensus tree of COI-COII spacer region sequences. Sequences from 48 honey bee workers collected in San Diego County (this study). Sequences labeled A, M, Y, and O are from Franck et al. (2001) and are used as representatives of the mitotype groups defined therein. All San Diego sequences are labeled (SD).
(DOCX)

## References

[1] Kerr WE. The history of the introduction of African bees to Brazil. S. Afr. Bee J. 1967;2: 3–5.

[2] Michener CD. The Brazilian bee problem. Annu. Rev. Entomol. 1975;20: 399–416. 1090242 10.1146/annurev.en.20.010175.002151

[3] Rinderer TE. African bees: the Africanization process and potential range in the United States. Bull. Entomol. Soc. Am. 1986;32: 222–227.

[4] Schneider SS, DeGrandi-Hoffman G, Smith DR. The African honey bee: factors contributing to a successful biological invasion. Annu. Rev. Entomol. 2004;49: 351–76. 10.1146/annurev.ento.49.061802.123359 14651468

[5] Pinto MA, Rubink WL, Patton JC, Coulson RN, Johnston JS. Africanization in the United States: replacement of feral European honeybees (*Apis mellifera* L.) by an African hybrid swarm. Genetics 2005;170: 1653–1665. 15937139 10.1534/genetics.104.035030 PMC1449774

[6] Winston ML. The biology and management of africanized honey bees. Annu. Rev. Entomol. 1992;37: 173–193.

[7] Lazaneo V. Bee Alert: Africanized Honey Bee Facts. University of California- Agriculture and Natural Resources 2002; Publication 8068.

[8] Szalanski AL, Magnus MM. Mitochondrial DNA characterization of Africanized honey bee (*Apis mellifera*) populations from the USA. J. Apic. Res. Bee World. 2010;49: 177–185. 10.3896/IBRA.1.49.2.06

[9] Franca FOS, Benvenuti LA, Fan HW, Santos D, Hain SH, Picchi-Martins FR, et al Severe and fatal mass attacks by 'killer' bees (Africanized bees-*Apis mellifera scutellata*) in Brazil: clinicopathological studies with measurements of serum venom concentrations. Q. J. Med. 1994;87: 269–282. 7938407

[10] Rinderer TE, Stelzer JA, Oldroyd BP, Buco SM, Rubink WL. Hybridization between European and africanized honey bees in the neotropical Yucatan peninsula. Science 1991;253: 309–311. 17794698 10.1126/science.253.5017.309

[11] Clarke KE, Rinderer TE, Franck P, Quezada-Euan JG, Oldroyd BP. The africanization of honeybees (Apis mellifera L.) of the Yucatan: a study of a massive hybridization event through time. Evolution 2002;56: 1462–1474. 12206246 10.1111/j.0014-3820.2002.tb01458.x

[12] Seguro JAL. Highly polymorphic DNA markers in an Africanized honey bee population in Costa Rica. Genet. Mol. Biol. 2000;23: 317–322.

[13] Pinto MA, Rubink WL, Coulson RN, Patton JC, Johnston JS. Temporal pattern of Africanization in a feral honeybee population from Texas inferred from mitochondrial DNA. Evolution 2004;58: 1047–1055. 15212385 10.1111/j.0014-3820.2004.tb00438.x

[14] Whitfield CW, Behura SK, Berlocher SH, Clark AG, Johnston JS, Sheppard WS, et al Thrice out of Africa: ancient and recent expansions of the honey bee, *Apis mellifera* . Science 2007;314: 642–645. 10.1126/science.1132772 17068261

[15] Rivera-Marchand B, Keularts J, Oskay D, Giray T. Coexistence of feral Africanized and European honey bees (Hymenoptera: Apoidea: Apodae) on St. Croix island. Caribb. J. Sci. 2008;44: 204–206.

[16] Smith DR, Taylor OR, Brown WM. Neotropical Africanized honey bees have African mitochondrial DNA. Nature 1989;339: 213–215. 2566123 10.1038/339213a0

[17] Diniz NM, Soares AEE, Sheppard WS, Del Lama MA. Genetic structure of honeybee populations from southern Brazil and Uruguay. Genet. Mol. Biol. 2003;26: 47–52.

[18] Rinderer TE, Buco SM, Rubink WL, Daly HV, Stelzer JA, Riggio RM, et al Morphometric identification of Africanized and European honey bees using large reference populations. Apidologie 1993;24: 569–85.

[19] Rinderer TE, Oldroyd BP, Sheppard WS. Africanized bees in the U.S. Sci. Am. 1993;269: 84–90.

[20] Sheppard WS, Rinderer TE, Mazzoli JA, Stelzer JA, Shimanuki H. Gene flow between African and European derived honey bee populations in Argentina. Nature 1991;349: 782–84.

[21] Quezada-Euán JJG, Medina LM. Hybridization between European and Africanized honey bees (Apis mellifera L.) in tropical Yucatan, Mexico. I. Morphometric changes in feral and managed colonies. Apidologie 1998;29: 555–568.

[22] Quezada-Euán JJG. Hybridization between European and Africanized honeybees in tropical Yucatan, Mexico. II. Morphometric, allozymic and mitochondrial DNA variability in feral colonies. Apidologie 2000;31: 443–453.

[23] Crozier YC, Koulianos S, Crozier RH. An improved test for Africanized honey bee mitochondrial DNA. Experientia 1991;47: 968–969. 1915781 10.1007/BF01929894

[24] Pinto MA, Johnston JS, Rubink WL, Coulson RN, Patton JC, Sheppard WS. Identification of Africanized honey bee (Hymenoptera: Apidae) mitochondrial DNA: validation of a rapid polymerase chain reaction-based assay. Ann. Entomol. Soc. Am. 2003;96: 679–684.

[25] Daly HV, Balling SS. Identification of Africanized honeybees in the western hemisphere by discriminant analysis. J. Kans. Entomol. Soc. 1978;51: 857–869.

[26] Harrison JF, Fewell JH, Anderson KE, Loper GM. Environmental physiology of the invasion of the Americas by Africanized honeybees. Integr. Comp. Biol. 2006;46: 1110–22. 10.1093/icb/icl046 21672812

[27] Schneider CA, Rasband WS, Eliceiri KW, NIH Image to ImageJ: 25 years of image analysis. Nature Methods 2012;9: 671–675. 22930834 10.1038/nmeth.2089 PMC5554542

[28] Pinto MA, Sheppard WS, Johnston JS, Rubink WL, Coulson RN, Schiff NM, et al Honey bees (Hymenoptera: Apidae) of African origin exist in non-Africanized areas of the Southern United States: evidence from mitochondrial DNA. Ann. Entomol. Soc. Am. 2007;100: 289–295.

[29] Garnery L, Solignac M, Celebrano G, Cornuet J-M. A simple test using restricted PCR-amplified mitochondrial DNA to study the genetic structure of *Apis mellifera* L. Experientia 1993;49: 1016–1021.

[30] Folmer O, Black M, Hoeh W, Lutz R, Vrijenhoek R. DNA primers for amplification of mitochondrial cytochrome c oxidase subunit I from diverse metazoan invertebrates. Mol. Mar. Biol. Biotechnol. 1994;3: 294–297. 7881515

[31] Tamura K, Stecher G, Peterson D, Filipski A, Kumar S. MEGA6: Molecular Evolutionary Genetics Analysis Version 6.0. Mol. Biol. Evol. 2013;30: 2725–2729. 10.1093/molbev/mst197 24132122 PMC3840312

[32] Franck P, Garnery L, Loiseau A, Oldroyd BP, Hepburn HR, Solignac M, Cornuet J-M. Genetic diversity of the honeybee in Africa: microsatellite and mitochondrial data. Heredity 2001;86: 420–430. 10.1046/j.1365-2540.2001.00842.x 11520342

[33] Ruttner F. Biogeography and Taxonomy of Honeybees. Springer-Verlag, Berlin, Heidelberg; 1988.

[34] Crozier RH, Crozier YC, The mitochondrial genome of the honeybee *Apis mellifera*: complete sequence and genome organization. Genetics 1993;133: 97–117. 8417993 10.1093/genetics/133.1.97 PMC1205303

[35] Ratnasingham S, Hebert PDN. BOLD: The Barcode of Life Data System (www.barcodinglife.org). Molecular Ecology Notes 2007;7: 355–364. 18784790 10.1111/j.1471-8286.2007.01678.x PMC1890991

[36] Taylor OR, Spivak M. Climatic limits of tropical African honeybees in the Americas. Bee World. 1984;65: 38–47

[37] Southwick EE, Roubik DW, Williams JM. Comparative Energy Balance in Groups of Africanized and European honey bees: Ecological implications. Comp. Biochem. Physiol., Part A: Mol. Integr. Physiol. 1990;97: 1–7. 10.1016/0300-9629(90)90713-3

[38] Nielsen DI, Ebert PR, Page RE, Hunt GJ, Guzmán-Novoa E. Improved polymerase chain reaction-based mitochondrial genotype assay for identification of the Africanized honey bee (Hymenoptera: Apidae). Ann. Entomol. Soc. Am. 2000;93: 1–6. 10.1603/0013-8746(2000)093[0001:IPCRBM]2.0.CO;2

[39] Daly HV, Hoelmer K, Gambino P. Clinal geographic variation in feral honey bees in California, USA. Apidologie 1991;22: 591–609.

[40] Steffan-Dewenter I, Kuhn A. Honeybee foraging in differentially structured landscapes. Proc. R. Soc. Lond. B. 2003;270: 569–575. 10.1098/rspb.2002.2292 PMC1691282 12769455

[41] Chapman NC, Harpur BA, Lim J, et al A SNP test to identify Africanized honeybees via proportion of ‘African’ ancestry. Mol. Ecol. Resources 2015; 10.1111/1755-0998.12411 25846634

[42] Rivera-Marchand B, Oskay D, Giray T. Gentle Africanized bees on an oceanic island. Evol. Appl. 2012;5: 746–756. 10.1111/j.1752-4571.2012.00252.x 23144660 PMC3492899

[43] Galindo-Cardona A, Acevedo-Gonzalez JP, Rivera-Marchand B, Giray T. Genetic structure of the gentle Africanized honey bee population (gAHB) in Puerto Rico. BMC Genet. 2013;14: 65 10.1186/1471-2156-14-65 23915100 PMC3750330

