# Supplementary material for: Range and Frequency of Africanized Honey Bees in California (USA)
Source: PLoS One. 2015 Sep 11;10(9):e0137407. doi: 10.1371/journal.pone.0137407 (PMC4567290; doi:10.1371/journal.pone.0137407)
Supplement: S1 Table — The number of honey bees, date collected, number carrying African mitochondria, location and elevation of collecting sites north of San Diego County. (DOCX) [file pone.0137407.s001.docx]

**Table S1a**. Number of bees collected, number carrying African mitochondria, location and elevation of collecting sites north of San Diego County.

| Total | African | Latitude | Longitude | Altitude (m) | Date Collected |
| --- | --- | --- | --- | --- | --- |
| 3 | 1 | 33.5014952 | -117.654329 | 33 | 4/29/14 |
| 3 | 1 | 33.8691762 | -118.310289 | 13 | 4/29/14 |
| 3 | 2 | 34.040507 | -118.89236 | 31 | 4/21/14 |
| 3 | 1 | 34.166476 | -118.8343201 | 279 | 4/29/14 |
| 3 | 2 | 34.3954 | -119.5228 | 2 | 8/14/13 |
| 3 | 0 | 34.415951 | -119.693796 | 3 | 4/21/14 |
| 3 | 0 | 34.4168922 | -119.5805265 | 29 | 4/29/14 |
| 3 | 0 | 34.435543 | -119.921524 | 22 | 4/21/14 |
| 3 | 1 | 34.5853848 | -120.4106752 | 102 | 4/29/14 |
| 3 | 2 | 34.8331629 | -118.8664723 | 1095 | 5/2/14 |
| 3 | 3 | 34.9985714 | -120.4106752 | 118 | 4/29/14 |
| 3 | 1 | 35.178032 | -120.748535 | 13 | 4/21/14 |
| 3 | 0 | 35.3671997 | -120.8423751 | 41 | 4/29/14 |
| 3 | 0 | 35.382854 | -119.0703167 | 120 | 5/2/14 |
| 3 | 2 | 35.3999003 | -119.4653163 | 82 | 5/2/14 |
| 1 | 1 | 35.4331876 | -120.2969051 | 515 | 5/2/14 |
| 3 | 0 | 35.539749 | -121.08833 | 22 | 8/15/13 |
| 3 | 0 | 35.6463521 | -120.6931743 | 220 | 5/2/14 |
| 3 | 0 | 35.6909008 | -121.2890437 | 10 | 4/29/14 |
| 3 | 0 | 35.767933 | -121.322457 | 41 | 4/21/14 |
| 3 | 0 | 35.8869709 | -121.4601547 | 71 | 4/29/14 |
| 3 | 0 | 35.9898 | -121.4955 | 49 | 8/15/13 |
| 3 | 0 | 36.0205 | -121.5493 | 109 | 8/16/13 |
| 3 | 0 | 36.0698 | -121.599 | 28 | 8/16/13 |
| 3 | 0 | 36.0724189 | -121.5966156 | 190 | 4/30/14 |
| 3 | 1 | 36.074014 | -121.59418 | 280 | 8/17/13 |
| 3 | 0 | 36.0754 | -121.5972 | 154 | 8/16/13 |
| 3 | 0 | 36.083298 | -121.588107 | 126 | 8/17/13 |
| 3 | 0 | 36.0931691 | -120.5155386 | 468 | 5/2/14 |
| 3 | 0 | 36.1296655 | -121.6436002 | 76 | 4/30/14 |
| 3 | 0 | 36.1420564 | -121.0097978 | 135 | 5/2/14 |
| 3 | 0 | 36.2543954 | -120.2548278 | 156 | 5/2/14 |
| 3 | 0 | 36.255516 | -119.8509768 | 61 | 5/2/14 |
| 3 | 0 | 36.3306652 | -121.8916089 | 40 | 4/30/14 |
| 3 | 0 | 36.406086 | -121.910569 | 50 | 4/21/14 |
| 3 | 0 | 36.4881977 | -121.9381654 | 45 | 4/30/14 |
| 3 | 0 | 36.8428606 | -119.8020664 | 106 | 5/2/14 |
| 3 | 0 | 36.9142171 | -121.7854695 | 12 | 4/30/14 |
| 3 | 0 | 36.970439 | -122.120133 | 25 | 4/21/14 |
| 3 | 0 | 37.0305 | -122.2178 | 34 | 8/20/13 |
| 3 | 0 | 37.0447142 | -121.262665 | 253 | 5/1/14 |
| 3 | 0 | 37.0533244 | -121.6064838 | 75 | 5/1/14 |
| 3 | 0 | 37.0563724 | -120.9339119 | 40 | 5/1/14 |
| 3 | 0 | 37.165359 | -122.1931326 | 421 | 4/30/14 |
| 1 | 0 | 37.174645 | -122.221055 | 341 | 8/19/13 |
| 3 | 0 | 37.2230959 | -120.4880029 | 45 | 5/1/14 |
| 3 | 0 | 37.2794082 | -122.0305477 | 113 | 4/30/14 |
| 3 | 1 | 37.3340837 | -119.6450222 | 725 | 5/2/14 |
| 3 | 0 | 37.4428 | -122.1718 | 23 | 8/5/13 |
| 3 | 0 | 37.4477802 | -119.7525757 | 778 | 5/2/14 |
| 3 | 0 | 37.629716 | -121.882486 | 91 | 5/1/14 |
| 3 | 0 | 37.7187379 | -122.4724069 | 71 | 4/30/14 |
| 3 | 0 | 37.751176 | -121.8714689 | 160 | 5/1/14 |
| 3 | 0 | 37.7977 | -121.1743717 | 17 | 5/1/14 |
| 3 | 1 | 37.91913 | -122.108974 | 158 | 8/1/14 |
| 3 | 0 | 37.9294 | -122.6821 | 4 | 8/21/13 |
| 3 | 0 | 37.9320057 | -120.4445573 | 402 | 5/1/14 |
| 3 | 0 | 38.025783 | -122.101731 | 1 | 8/1/14 |
| 3 | 1 | 38.1077738 | -120.5979278 | 366 | 5/1/14 |
| 3 | 1 | 38.183884 | -121.8433948 | 53 | 5/1/14 |
| 3 | 0 | 38.201294 | -121.0556449 | 44 | 5/1/14 |
| 3 | 0 | 38.2352148 | -122.6248919 | 4 | 5/1/14 |
| 3 | 1 | 38.3138 | -121.755951 | 4 | 8/1/14 |
| 3 | 0 | 38.519558 | -121.9759771 | 41 | 8/1/14 |
| 3 | 0 | 38.5531272 | -121.7478616 | 14 | 8/1/14 |
| 3 | 0 | 38.6573079 | -121.5235487 | 7 | 7/30/14 |
| 3 | 0 | 38.6953432 | -122.0304539 | 64 | 8/1/14 |
| 1 | 0 | 38.9942428 | -122.5499173 | 311 | 8/1/14 |
| 3 | 0 | 39.141744 | -121.6322062 | 18 | 7/30/14 |
| 3 | 0 | 39.1527542 | -122.2362166 | 39 | 8/1/14 |
| 3 | 0 | 39.1689176 | -122.9593209 | 412 | 8/1/14 |
| 3 | 0 | 39.3934224 | -123.4474478 | 578 | 8/1/14 |
| 3 | 0 | 39.4124563 | -123.8078936 | 28 | 8/1/14 |
| 3 | 0 | 39.760755 | -121.84927 | 61 | 7/30/14 |
| 3 | 0 | 40.0544811 | -123.7925558 | 141 | 7/31/14 |
| 3 | 0 | 40.1860723 | -122.2022258 | 83 | 7/30/14 |
| 3 | 0 | 40.4475674 | -124.0403024 | 82 | 7/31/14 |
| 1 | 0 | 40.5585581 | -122.3538305 | 165 | 7/30/14 |
| 3 | 0 | 40.5928179 | -122.3772612 | 147 | 7/30/14 |
| 3 | 0 | 40.8069288 | -124.1459355 | 3 | 7/31/14 |
| 3 | 0 | 41.2390726 | -124.0839373 | 9 | 7/31/14 |
| 3 | 0 | 41.3035529 | -122.3079945 | 1075 | 7/30/14 |
| 3 | 0 | 41.6125315 | -124.10597 | 74 | 7/31/14 |
| 3 | 0 | 41.739219 | -122.633711 | 790 | 7/30/14 |
| 3 | 0 | 41.7467898 | -124.2008183 | 6 | 7/31/14 |
| 3 | 0 | 41.8090867 | -124.0483654 | 66 | 7/31/14 |
| 3 | 0 | 41.8889962 | -123.80267 | 346 | 7/31/14 |
| 3 | 0 | 42.1821713 | -122.6857009 | 627 | 7/30/14 |
| 3 | 0 | 42.2192247 | -123.6479904 | 376 | 7/31/14 |
| 3 | 0 | 42.3312321 | -122.8668634 | 415 | 7/31/14 |
| 3 | 0 | 42.4375903 | -123.3133986 | 288 | 7/31/14 |
| 265 | 23 |  |  |  |  |

**Table S1b**. Number of bees collected, number carrying African mitochondria, location and elevation of collecting sites in San Diego County. Bees with unknown colletion dates were kindly collected by colleagues during spring and summer of 2013

| Total | African | Latitude | Longitude | Altitude (m) | Date Collected |
| --- | --- | --- | --- | --- | --- |
| 3 | 1 | 32.563875 | -116.9668852 | 149 | 4/11/13 |
| 3 | 3 | 32.5766379 | -117.0843878 | 9 | 4/11/13 |
| 3 | 2 | 32.5908726 | -116.5243876 | 676 | 5/16/13 |
| 3 | 2 | 32.5961128 | -116.6523967 | 517 | 4/11/13 |
| 3 | 1 | 32.6109821 | -116.6364695 | 741 | 4/11/13 |
| 3 | 1 | 32.6140925 | -116.7145607 | 288 | 4/11/13 |
| 3 | 3 | 32.6273939 | -116.9636611 | 195 | 4/11/13 |
| 3 | 2 | 32.6309694 | -116.436177 | 826 | 5/16/13 |
| 3 | 0 | 32.6340543 | -116.9172117 | 156 | 4/11/13 |
| 2 | 0 | 32.6408321 | -116.772593 | 348 | 4/11/13 |
| 3 | 3 | 32.6523514 | -116.8549418 | 194 | 4/11/13 |
| 3 | 3 | 32.6524828 | -116.396316 | 953 | 5/16/13 |
| 3 | 2 | 32.6689 | -117.1084 | 6 |  |
| 3 | 1 | 32.6693213 | -116.3071929 | 1145 | 5/16/13 |
| 3 | 1 | 32.7026445 | -117.1457487 | 15 | 4/11/13 |
| 3 | 3 | 32.7216 | -117.0647 | 116 |  |
| 3 | 0 | 32.7279406 | -117.1515297 | 76 | 4/11/13 |
| 3 | 3 | 32.7384 | -116.91 | 151 | 4/13/13 |
| 3 | 3 | 32.7436393 | -115.9943339 | 114 | 4/9/13 |
| 3 | 1 | 32.7465 | -117.1795 | 55 |  |
| 3 | 2 | 32.7627932 | -117.2321295 | 3 | 4/11/13 |
| 1 | 0 | 32.764644 | -116.68729 | 760 | 4/4/13 |
| 3 | 2 | 32.767061 | -117.150974 | 14 | 4/4/13 |
| 3 | 3 | 32.772696 | -117.021148 | 158 | 4/4/13 |
| 3 | 3 | 32.780344 | -116.872045 | 144 | 4/4/13 |
| 3 | 2 | 32.7829 | -116.9972 | 201 |  |
| 3 | 3 | 32.793394 | -116.805916 | 456 | 4/4/13 |
| 3 | 3 | 32.7938948 | -116.1082163 | 288 | 4/9/13 |
| 3 | 2 | 32.795368 | -116.943229 | 143 | 4/4/13 |
| 3 | 3 | 32.7966361 | -117.2536033 | 8 | 4/11/13 |
| 3 | 1 | 32.798116 | -116.747611 | 428 | 4/4/13 |
| 3 | 1 | 32.799973 | -117.137015 | 116 | 3/8/12 |
| 2 | 2 | 32.807893 | -116.640025 | 969 | 4/4/13 |
| 3 | 2 | 32.8269748 | -116.1655539 | 379 | 4/9/13 |
| 3 | 0 | 32.8300494 | -117.2768857 | 23 | 4/11/13 |
| 4 | 1 | 32.833602 | -116.865411 | 263 | 4/4/13 |
| 3 | 2 | 32.839024 | -117.044578 | 86 | 4/4/13 |
| 3 | 3 | 32.841553 | -116.540304 | 1134 | 4/4/13 |
| 3 | 2 | 32.8532228 | -117.256651 | 4 | 4/11/13 |
| 3 | 3 | 32.8547 | -117.2079 | 115 |  |
| 2 | 2 | 32.855621 | -116.939859 | 115 | 4/4/13 |
| 3 | 1 | 32.856349 | -116.809354 | 431 | 4/4/13 |
| 3 | 3 | 32.858339 | -116.819531 | 377 | 4/4/13 |
| 3 | 0 | 32.8673 | -116.6106 | 1086 | 9/13/13 |
| 3 | 2 | 32.8717995 | -116.2092469 | 217 | 4/9/13 |
| 3 | 1 | 32.8753 | -117.2483 | 107 | 4/17/12 |
| 3 | 2 | 32.876784 | -117.248458 | 115 | 4/2/13 |
| 3 | 2 | 32.879 | -117.2404 | 128 |  |
| 3 | 3 | 32.8914 | -117.0917 | 231 | 3/20/12 |
| 3 | 3 | 32.902 | -116.552 | 1440 | 8/3/13 |
| 3 | 0 | 32.905 | -116.572 | 1290 | 5/27/13 |
| 3 | 3 | 32.911198 | -116.2358018 | 297 | 4/9/13 |
| 3 | 2 | 32.9194 | -116.5725 | 1244 | 9/13/13 |
| 3 | 2 | 32.930002 | -116.970101 | 362 | 4/3/13 |
| 3 | 3 | 32.93859 | -117.258136 | 13 | 4/2/13 |
| 3 | 2 | 32.938807 | -117.258201 | 14 | 4/2/13 |
| 3 | 3 | 32.938807 | -117.258201 | 14 | 4/2/13 |
| 3 | 3 | 32.939945 | -117.050897 | 215 | 4/3/13 |
| 3 | 3 | 32.942221 | -117.223124 | 36 | 4/3/13 |
| 3 | 3 | 32.9509709 | -116.2878892 | 359 | 4/9/13 |
| 3 | 2 | 32.956412 | -117.108975 | 197 | 4/3/13 |
| 3 | 1 | 32.9605 | -116.58128 | 1476 | 6/29/13 |
| 3 | 1 | 32.9755287 | -116.3503291 | 467 | 4/9/13 |
| 3 | 2 | 32.978978 | -116.4231394 | 611 | 4/9/13 |
| 2 | 1 | 32.9922 | -117.114 | 240 |  |
| 3 | 2 | 32.994317 | -117.212593 | 10 | 4/3/13 |
| 3 | 1 | 32.994459 | -116.975378 | 541 | 4/3/13 |
| 3 | 2 | 33.016028 | -116.903691 | 433 | 4/3/13 |
| 3 | 3 | 33.038 | -116.4088 | 775 | 8/17/13 |
| 3 | 1 | 33.04759 | -117.293885 | 19 | 4/2/13 |
| 3 | 1 | 33.047774 | -116.870501 | 441 | 4/3/13 |
| 3 | 3 | 33.068312 | -117.063507 | 106 | 4/3/13 |
| 3 | 2 | 33.0688 | -116.1162 | 70 |  |
| 3 | 3 | 33.0688 | -116.1162 | 70 |  |
| 3 | 1 | 33.068962 | -117.119074 | 104 | 4/3/13 |
| 3 | 0 | 33.094486 | -116.960706 | 128 | 4/3/13 |
| 3 | 2 | 33.106001 | -116.789635 | 693 | 4/3/13 |
| 3 | 2 | 33.108 | -116.4976 | 732 |  |
| 3 | 1 | 33.1174759 | -116.4370251 | 595 | 4/9/13 |
| 3 | 2 | 33.1224 | -116.2376 | 324 | 5/16/13 |
| 3 | 3 | 33.1224 | -116.2376 | 324 | 3/2/13 |
| 3 | 1 | 33.1324 | -116.3448 | 386 | 5/16/13 |
| 3 | 3 | 33.1324 | -116.3448 | 386 | 3/23/13 |
| 3 | 2 | 33.1338 | -116.3665 | 423 | 5/16/13 |
| 3 | 2 | 33.1338 | -116.3665 | 423 | 3/2/13 |
| 3 | 2 | 33.1367 | -116.38 | 442 | 5/11/13 |
| 3 | 1 | 33.1369 | -116.3796 | 438 |  |
| 3 | 3 | 33.1501785 | -116.3492812 | 513 | 4/9/13 |
| 3 | 1 | 33.1511736 | -117.0390338 | 221 | 5/16/13 |
| 3 | 3 | 33.1536 | -116.5475 | 827 | 5/11/13 |
| 3 | 1 | 33.1559 | -116.3449 | 479 |  |
| 3 | 2 | 33.2101 | -116.4894 | 1241 | 5/11/13 |
| 3 | 2 | 33.2119256 | -116.3656794 | 211 | 4/9/13 |
| 3 | 1 | 33.231234 | -117.317748 | 26 | 4/2/13 |
| 3 | 1 | 33.2505283 | -116.4055112 | 265 | 4/9/13 |
| 3 | 1 | 33.258141 | -117.23706 | 70 | 4/2/13 |
| 3 | 3 | 33.2762 | -116.4259 | 317 | 5/11/13 |
| 3 | 3 | 33.305764 | -116.869751 | 1443 | 4/2/13 |
| 3 | 3 | 33.3348 | -116.9197 | 1654 | 7/19/13 |
| 3 | 1 | 33.346081 | -117.024321 | 219 | 4/2/13 |
| 3 | 3 | 33.361858 | -117.103829 | 109 | 4/2/13 |
| 298 | 193 |  |  |  |  |
