# Supplementary material for: Range and Frequency of Africanized Honey Bees in California (USA)
Source: PLoS One. 2015 Sep 11;10(9):e0137407. doi: 10.1371/journal.pone.0137407 (PMC4567290; doi:10.1371/journal.pone.0137407)
Supplement: S2 Table — (DOCX) [file pone.0137407.s002.docx]

**Table S2a**. Morphometric measurements, discriminant function scores and outcomes, and cytochrome b restriction results for honey bees collected north of San Diego County: FWL, fore wing length; HWL, hind wing lenghth; TL, tibia length; FL, femur length (all in mm).

| Site | Latitude | Longtitude | Alti-tude | FWL | HWL | TL | FL | Discriminant score | Discriminant function result | Cyt-b result |
| --- | --- | --- | --- | --- | --- | --- | --- | --- | --- | --- |
| 1 | 36.07540 | -121.59720 | 154 | 9.332 | 4.569 | 3.366 | 2.612 | -0.5615709 | European | European |
|  | 36.07540 | -121.59720 | 154 | 9.622 | 4.548 | 3.364 | 2.745 | 2.3376611 | European | European |
|  | 36.07540 | -121.59720 | 154 | 9.206 | 4.288 | 3.176 | 2.734 | 2.7944146 | European | European |
| 2 | 37.92940 | -122.68210 | 4 | Frayed | 4.124 | 3.193 | 2.612 |  | no result | European |
|  | 37.92940 | -122.68210 | 4 | 8.902 | 4.158 | 3.312 | 2.733 | 0.4085765 | European | European |
|  | 37.92940 | -122.68210 | 4 | 9.132 | 4.347 | 3.313 | 2.717 | 0.9450156 | European | European |
| 3 | 36.02050 | -121.54930 | 109 | 9.11 | 4.36 | 3.143 | 2.754 | 3.3182378 | European | European |
|  | 36.02050 | -121.54930 | 109 | 9.228 | 4.461 | 3.141 | 2.581 | 0.9317554 | European | European |
|  | 36.02050 | -121.54930 | 109 | 9.274 | 4.407 | 3.147 | 2.631 | 1.7352326 | European | European |
| 4 | 35.98980 | -121.49550 | 49 | 9.338 | 4.392 | 3.193 | 2.588 | 0.6860184 | European | European |
|  | 35.98980 | -121.49550 | 49 | 9.482 | 4.487 | 3.097 | 2.569 | 1.874374 | European | European |
|  | 35.98980 | -121.49550 | 49 | Frayed | 4.289 | 3.114 | 2.456 |  | no result | European |
| 5 | 34.39540 | -119.52280 | 2 | 9.025 | 4.229 | 3.24 | 2.31 | -5.3432839 | African | African |
|  | 34.39540 | -119.52280 | 2 | 9.122 | 4.293 | 3.277 | 2.739 | 1.5966404 | European | African |
|  | 34.39540 | -119.52280 | 2 | 9.398 | 4.35 | 3.164 | 2.404 | -1.9129106 | African | European |
| 6 | 36.06980 | -121.59900 | 28 | 9.398 | 4.403 | 3.139 | 2.77 | 4.3992895 | European | European |
|  | 36.06980 | -121.59900 | 28 | 9.455 | 4.536 | 3.204 | 2.781 | 4.1929079 | European | European |
|  | 36.06980 | -121.59900 | 28 | 9.551 | 4.433 | 3.308 | 2.665 | 1.3072498 | European | European |
| 7 | 37.03050 | -122.21780 | 34 | 9.268 | 4.284 | 3.285 | 2.767 | 2.3256361 | European | European |
|  | 37.03050 | -122.21780 | 34 | 9.083 | 4.198 | 3.199 | 2.633 | 0.4801137 | European | European |
|  | 37.03050 | -122.21780 | 34 | 9.342 | 4.254 | 3.129 | 2.747 | 3.8076483 | European | European |
| 8 | 36.08330 | -121.58811 | 126 | 9.747 | 4.641 | 3.2 | 2.745 | 4.5095282 | European | European |
|  | 36.08330 | -121.58811 | 126 | 9.271 | 4.497 | 3.167 | 2.436 | -1.5626581 | African | European |
|  | 36.08330 | -121.58811 | 126 | 9.545 | 4.556 | 3.133 | 2.749 | 4.6758247 | European | European |
| 9 | 35.53975 | -121.08833 | 22 | 9.069 | 4.262 | no leg | no leg |  | no result | European |
|  | 35.53975 | -121.08833 | 22 | 9.323 | 4.287 | 3.143 | 2.65 | 2.0657047 | European | European |
|  | 35.53975 | -121.08833 | 22 | 9.159 | 4.305 | 3.12 | 2.676 | 2.3444615 | European | European |
| 10 | 36.07401 | -121.59418 | 280 | 9.284 | 4.505 | 3.188 | 2.778 | 3.8460485 | European | European |
|  | 36.07401 | -121.59418 | 280 | 9.353 | 4.41 | 3.293 | 2.633 | 0.4175661 | European | African |
|  | 36.07401 | -121.59418 | 280 | 9.065 | 4.423 | 3.041 | 2.662 | 2.8627939 | European | European |
| 11 | 37.17465 | -122.22106 | 341 | 9.223 | 4.285 | 3.162 | 2.862 | 5.0744633 | European | European |
| 12 | 37.44280 | -122.17180 | 23 | 9.043 | 4.242 | 3.153 | 2.63 | 0.8731632 | European | European |
|  | 37.44280 | -122.17180 | 23 | 9.036 | 4.415 | 3.115 | 2.719 | 2.924659 | European | European |
|  | 37.44280 | -122.17180 | 23 | 9.094 | 4.274 | 3.213 | 2.54 | -1.0686786 | African | European |
| 13 | 34.04051 | -118.89236 | 31 | 8.658 | 4.086 | 3.102 | 2.51 | -1.7041836 | African | African |
|  | 34.04051 | -118.89236 | 31 | 9.192 | 4.301 | 3.179 | 2.576 | 0.1607487 | European | European |
|  | 34.04051 | -118.89236 | 31 | 8.849 | 4.216 | 3.175 | 2.55 | -1.188127 | African | African |
| 14 | 36.40609 | -121.91057 | 50 | 9.173 | 4.358 | 3.243 | 2.648 | 0.6783258 | European | European |
|  | 36.40609 | -121.91057 | 50 | 9.114 | 4.332 | 3.372 | 2.701 | -0.0075209 | European | European |
|  | 36.40609 | -121.91057 | 50 | 9.16 | 4.385 | 3.179 | 2.621 | 0.917835 | European | European |
| 15 | 34.41595 | -119.69380 | 3 | 9.018 | 4.214 | 3.022 | 2.486 | -0.18405 | European | European |
|  | 34.41595 | -119.69380 | 3 | 9.428 | 4.407 | 3.218 | 2.693 | 2.3809524 | European | European |
|  | 34.41595 | -119.69380 | 3 | 9.148 | 4.359 | 3.234 | 2.548 | -0.9220379 | African | European |
| 16 | 36.97044 | -122.12013 | 25 | 9.019 | 4.293 | 3.276 | 2.635 | -0.3516788 | European | European |
|  | 36.97044 | -122.12013 | 25 | 9.119 | 4.441 | 3.176 | 2.595 | 0.4897184 | European | European |
|  | 36.97044 | -122.12013 | 25 | 9.504 | 4.497 | 3.311 | 2.716 | 2.0684287 | European | European |
| 17 | 35.17803 | -120.74854 | 13 | 9.157 | 4.249 | 3.296 | 2.639 | -0.2052516 | European | European |
|  | 35.17803 | -120.74854 | 13 | 9.531 | 4.429 | 3.222 | 2.685 | 2.4935978 | European | African |
|  | 35.17803 | -120.74854 | 13 | 8.882 | 4.2 | 3.154 | 2.533 | -1.1790389 | African | European |
| 18 | 34.43554 | -119.92152 | 22 | 9.378 | 4.728 | 3.376 | 2.745 | 1.8148943 | European | European |
|  | 34.43554 | -119.92152 | 22 | 9.569 | 4.521 | 3.28 | 2.664 | 1.7409971 | European | European |
|  | 34.43554 | -119.92152 | 22 | 9.304 | 4.444 | 3.357 | 2.581 | -1.1949581 | African | European |
| 19 | 35.76793 | -121.32246 | 41 | 9.041 | Frayed | 3.289 | 2.657 |  | no result | European |
|  | 35.76793 | -121.32246 | 41 | 9.218 | 4.353 | 3.22 | 2.652 | 1.0954787 | European | European |
|  | 35.76793 | -121.32246 | 41 | 9.253 | 4.373 | 3.156 | 2.614 | 1.2674809 | European | European |
| 20 | 33.50150 | -117.65433 | 33 | 8.815 | 4.117 | 3.111 | 2.526 | -1.1056339 | African | African |
|  | 33.50150 | -117.65433 | 33 | 8.796 | 4.225 | 3.002 | 2.44 | -1.2684233 | African | European |
|  | 33.50150 | -117.65433 | 33 | 9.145 | 4.342 | 3.116 | 2.449 | -1.3133027 | African | European |
| 21 | 33.86918 | -118.31029 | 13 | 8.427 | 4.03 | 3.01 | 2.439 | -2.5355041 | African | European |
|  | 33.86918 | -118.31029 | 13 | 8.807 | 4.134 | 3.06 | 2.56 | -0.0069866 | European | European |
|  | 33.86918 | -118.31029 | 13 | 9.022 | 4.377 | 3.288 | 2.598 | -0.9743455 | African | African |
| 22 | 34.16648 | -118.83432 | 279 | 8.897 | 4.31 | 3.236 | 2.503 | -2.3699801 | African | European |
|  | 34.16648 | -118.83432 | 279 | 8.543 | 4.276 | 3.184 | 2.536 | -2.2097264 | African | African |
|  | 34.16648 | -118.83432 | 279 | 9.134 | 4.446 | 3.387 | 2.713 | 0.2180125 | European | European |
| 23 | 34.41689 | -119.58053 | 29 | 8.546 | 4.154 | 3.237 | 2.576 | -2.2604478 | African | European |
|  | 34.41689 | -119.58053 | 29 | 9.315 | 4.378 | 3.08 | 2.657 | 2.9406705 | European | European |
|  | 34.41689 | -119.58053 | 29 | 9.311 | 4.517 | 3.324 | 2.631 | 0.0795872 | European | European |
| 24 | 34.58538 | -120.41068 | 102 | 8.936 | 4.298 | 3.27 | 2.697 | 0.5226749 | European | African |
|  | 34.58538 | -120.41068 | 102 | 9.242 | 4.319 | 3.247 | 2.606 | 0.0755511 | European | European |
|  | 34.58538 | -120.41068 | 102 | 9.168 | 4.213 | 3.191 | 2.646 | 1.0098017 | European | European |
| 25 | 34.99857 | -120.41068 | 118 | 9.306 | 4.463 | 3.187 | 2.636 | 1.5401433 | European | African |
|  | 34.99857 | -120.41068 | 118 | 8.581 | 4.254 | 2.959 | 2.357 | -2.6734011 | African | African |
|  | 34.99857 | -120.41068 | 118 | 8.921 | 4.225 | 3.089 | 2.437 | -1.9282022 | African | African |
| 26 | 35.36720 | -120.84238 | 41 | 9.036 | 4.188 | 3.143 | 2.578 | 0.046363 | European | European |
|  | 35.36720 | -120.84238 | 41 | 8.988 | 4.252 | 3.165 | 2.631 | 0.6356369 | European | European |
|  | 35.36720 | -120.84238 | 41 | 9.304 | 4.348 | 3.103 | 2.606 | 1.7983554 | European | European |
| 27 | 35.69090 | -121.28904 | 10 | 9.194 | 4.252 | 3.189 | 2.636 | 0.9804804 | European | European |
|  | 35.69090 | -121.28904 | 10 | 9.22 | 4.337 | 3.219 | 2.61 | 0.4052489 | European | European |
|  | 35.69090 | -121.28904 | 10 | 8.944 | 4.359 | 3.087 | 2.511 | -0.4766746 | European | European |
| 28 | 35.88697 | -121.46015 | 71 | 9.049 | 4.411 | 3.218 | 2.625 | 0.3207152 | European | European |
|  | 35.88697 | -121.46015 | 71 | 9.139 | 4.524 | 3.149 | 2.507 | -0.5101359 | European | European |
|  | 35.88697 | -121.46015 | 71 | 9.023 | 4.333 | 3.146 | 2.58 | 0.1907363 | European | European |
| 29 | 36.07242 | -121.59662 | 190 | 9.077 | 4.298 | 3.15 | 2.667 | 1.6634423 | European | European |
|  | 36.07242 | -121.59662 | 190 | 8.908 | 4.215 | 3.092 | 2.664 | 1.7050841 | European | European |
|  | 36.07242 | -121.59662 | 190 | 8.976 | 4.569 | 3.133 | 2.52 | -0.4829533 | European | European |
| 30 | 36.12967 | -121.64360 | 76 | 8.792 | 4.242 | 3.024 | 2.591 | 0.9761271 | European | European |
|  | 36.12967 | -121.64360 | 76 | 9.056 | 4.28 | 3.146 | 2.606 | 0.6342762 | European | European |
|  | 36.12967 | -121.64360 | 76 | 9.081 | Frayed | 3.318 | 2.579 |  | no result | European |
| 31 | 36.33067 | -121.89161 | 40 | 8.821 | 4.192 | 3.212 | 2.673 | 0.3290147 | European | European |
|  | 36.33067 | -121.89161 | 40 | 9.211 | 4.342 | 3.178 | 2.509 | -0.8259935 | African | European |
|  | 36.33067 | -121.89161 | 40 | 9.171 | 4.247 | 3.163 | 2.629 | 1.078642 | European | European |
| 32 | 36.48820 | -121.93817 | 45 | 9.268 | 4.348 | 3.236 | 2.71 | 1.9929958 | European | European |
|  | 36.48820 | -121.93817 | 45 | 9.043 | 4.387 | 3.159 | 2.683 | 1.8518818 | European | European |
|  | 36.48820 | -121.93817 | 45 | 9.117 | 4.222 | 3.197 | 2.606 | 0.1748388 | European | European |
| 33 | 36.91422 | -121.78547 | 12 | 9.069 | 4.346 | 3.154 | 2.57 | 0.0737736 | European | European |
|  | 36.91422 | -121.78547 | 12 | 9.287 | 4.486 | 3.229 | 2.643 | 1.1880095 | European | European |
|  | 36.91422 | -121.78547 | 12 | 9.203 | 4.255 | 3.173 | 2.741 | 2.8930548 | European | European |
| 34 | 37.16536 | -122.19313 | 421 | 9.106 | 4.309 | 2.965 | 2.612 | 2.8184643 | European | European |
|  | 37.16536 | -122.19313 | 421 | 8.771 | 4.215 | 2.996 | 2.576 | 0.9430917 | European | European |
|  | 37.16536 | -122.19313 | 421 | 8.928 | 4.259 | 3.181 | 2.636 | 0.4047141 | European | European |
| 35 | 37.27941 | -122.03055 | 113 | 9.034 | 4.321 | 3.182 | 2.705 | 1.8639318 | European | European |
|  | 37.27941 | -122.03055 | 113 | 9.489 | 4.384 | 3.267 | 2.741 | 2.7698499 | European | European |
|  | 37.27941 | -122.03055 | 113 | 8.949 | 4.25 | 3.067 | 2.549 | 0.2371545 | European | European |
| 36 | 37.71874 | -122.47241 | 71 | 8.998 | 4.183 | 3.14 | 2.672 | 1.5128937 | European | European |
|  | 37.71874 | -122.47241 | 71 | 8.614 | 3.972 | 3.08 | 2.498 | -1.9156614 | African | European |
|  | 37.71874 | -122.47241 | 71 | 8.815 | Frayed | 3.18 | 2.701 |  | no result | European |
| 37 | 38.23521 | -122.62489 | 4 | 9.318 | 4.442 | 3.215 | 2.688 | 2.0968922 | European | European |
|  | 38.23521 | -122.62489 | 4 | 9.319 | 4.266 | 3.22 | 2.514 | -1.0116064 | African | European |
|  | 38.23521 | -122.62489 | 4 | 9.522 | 4.369 | 3.208 | 2.753 | 3.6582798 | European | European |
| 38 | 38.18388 | -121.84339 | 53 | 9.349 | 4.339 | 3.14 | 2.562 | 0.7880015 | European | European |
|  | 38.18388 | -121.84339 | 53 | 9.467 | 4.46 | 3.092 | 2.508 | 0.8599988 | European | European |
|  | 38.18388 | -121.84339 | 53 | 9.14 | 4.233 | 3.137 | 2.571 | 0.3121904 | European | African |
| 39 | 38.20129 | -121.05564 | 44 | Frayed | Frayed | no leg | no leg |  | no result | European |
|  | 38.20129 | -121.05564 | 44 | Frayed | Frayed | 3.195 | 2.659 |  | no result | European |
|  | 38.20129 | -121.05564 | 44 | Frayed | Frayed | 3.268 | 2.683 |  | no result | European |
| 40 | 38.10777 | -120.59793 | 366 | 9.148 | 4.284 | 3.164 | 2.544 | -0.334114 | European | European |
|  | 38.10777 | -120.59793 | 366 | 8.824 | 4.231 | 3.136 | 2.485 | -1.8803636 | African | African |
|  | 38.10777 | -120.59793 | 366 | 9.029 | 4.21 | 3.186 | 2.648 | 0.7422402 | European | European |
| 41 | 37.93201 | -120.44456 | 402 | Frayed | Frayed | no leg | no leg |  | no result | European |
|  | 37.93201 | -120.44456 | 402 | 9.086 | 4.31 | 3.203 | 2.566 | -0.51374 | European | European |
|  | 37.93201 | -120.44456 | 402 | 9.274 | 4.417 | 3.21 | 2.692 | 2.0743267 | European | European |
| 42 | 37.79770 | -121.17437 | 17 | 8.958 | 4.327 | 3.027 | 2.562 | 0.9913211 | European | European |
|  | 37.79770 | -121.17437 | 17 | 9.163 | 4.257 | 3.074 | 2.39 | -1.8889539 | African | European |
|  | 37.79770 | -121.17437 | 17 | 9.207 | 4.291 | 3.2 | 2.549 | -0.4782972 | European | European |
| 43 | 37.75118 | -121.87147 | 160 | 9.138 | 4.436 | 3.055 | 2.438 | -0.7476342 | African | European |
|  | 37.75118 | -121.87147 | 160 | 9.125 | 4.24 | 3.171 | 2.692 | 1.8989572 | European | European |
|  | 37.75118 | -121.87147 | 160 | 9.012 | 4.316 | 3.062 | 2.523 | 0.1041737 | European | European |
| 44 | 37.62972 | -121.88249 | 91 | 9.158 | 4.243 | 3.036 | 2.442 | -0.6645229 | African | European |
|  | 37.62972 | -121.88249 | 91 | 9.134 | 4.348 | 3.24 | 2.593 | -0.2989805 | African | European |
|  | 37.62972 | -121.88249 | 91 | 9.147 | 4.193 | 3.133 | 2.522 | -0.4771395 | European | European |
| 45 | 37.05332 | -121.60648 | 75 | 8.939 | 4.21 | 3.169 | 2.579 | -0.4311597 | European | European |
|  | 37.05332 | -121.60648 | 75 | 9.14 | 4.236 | 3.179 | 2.637 | 0.9478403 | European | European |
|  | 37.05332 | -121.60648 | 75 | Frayed | Frayed | 3.154 | 2.588 |  | no result | European |
| 46 | 37.04471 | -121.26267 | 253 | Frayed | Frayed | 3.077 | 2.461 |  | no result | European |
|  | 37.04471 | -121.26267 | 253 | 9.307 | 4.559 | 3.251 | 2.576 | -0.0019263 | European | European |
|  | 37.04471 | -121.26267 | 253 | Frayed | Frayed | 3.108 | 2.465 |  | no result | European |
| 47 | 37.05637 | -120.93391 | 40 | 9.016 | 4.283 | 3.147 | 2.551 | -0.3722822 | European | European |
|  | 37.05637 | -120.93391 | 40 | 8.608 | 4.329 | 3.036 | 2.451 | -1.7968804 | African | European |
|  | 37.05637 | -120.93391 | 40 | 9.05 | 4.123 | 2.988 | 2.517 | 0.6540992 | European | European |
| 48 | 37.22310 | -120.48800 | 45 | Frayed | Frayed | no leg | no leg |  | no result | European |
|  | 37.22310 | -120.48800 | 45 | 9.037 | 4.265 | 3.113 | 2.594 | 0.7230741 | European | European |
|  | 37.22310 | -120.48800 | 45 | Frayed | Frayed | no leg | no leg |  | no result | European |
| 49 | 37.33408 | -119.64502 | 725 | 8.891 | 4.333 | 3.159 | 2.559 | -0.6229132 | European | European |
|  | 37.33408 | -119.64502 | 725 | 8.553 | 4.128 | 3.113 | 2.483 | -2.4756147 | African | African |
|  | 37.33408 | -119.64502 | 725 | 9.019 | 4.398 | 2.994 | 2.405 | -0.9838671 | African | European |
| 50 | 37.44778 | -119.75258 | 778 | 9.184 | 4.309 | 3.129 | 2.631 | 1.5810392 | European | European |
|  | 37.44778 | -119.75258 | 778 | 8.881 | 4.221 | 3.193 | 2.516 | -1.8486561 | African | European |
|  | 37.44778 | -119.75258 | 778 | 8.917 | 4.481 | 3.281 | 2.599 | -1.0213208 | African | European |
| 51 | 36.84286 | -119.80207 | 106 | 9.135 | 4.327 | 3.169 | 2.621 | 0.8907588 | European | European |
|  | 36.84286 | -119.80207 | 106 | 9.398 | 4.353 | 3.208 | 2.7 | 2.4605651 | European | European |
|  | 36.84286 | -119.80207 | 106 | 8.954 | 4.332 | 3.214 | 2.603 | -0.3314223 | European | European |
| 52 | 36.25552 | -119.85098 | 61 | 9.11 | 4.299 | 3.259 | 2.587 | -0.719093 | African | European |
|  | 36.25552 | -119.85098 | 61 | 8.923 | 4.274 | 3.227 | 2.541 | -1.6315375 | African | European |
|  | 36.25552 | -119.85098 | 61 | 9.359 | 4.324 | 3.141 | 2.632 | 1.9283644 | European | European |
| 53 | 36.25440 | -120.25483 | 156 | 9.243 | 4.446 | 3.288 | 2.675 | 0.9241563 | European | European |
|  | 36.25440 | -120.25483 | 156 | 9.507 | 4.513 | 3.196 | 2.611 | 1.6024168 | European | European |
|  | 36.25440 | -120.25483 | 156 | 9.108 | 4.175 | 3.096 | 2.583 | 0.7933298 | European | European |
| 54 | 36.09317 | -120.51554 | 468 | 9.034 | 4.16 | 2.978 | 2.466 | -0.0683578 | African | European |
|  | 36.09317 | -120.51554 | 468 | 9.329 | 4.53 | 3.207 | 2.657 | 1.8099957 | European | European |
|  | 36.09317 | -120.51554 | 468 | 9.125 | 4.297 | 3.162 | 2.661 | 1.557323 | European | European |
| 55 | 36.14206 | -121.00980 | 135 | 9.04 | 4.281 | 3.111 | 2.492 | -0.8957289 | African | European |
|  | 36.14206 | -121.00980 | 135 | 8.912 | 4.218 | 3.031 | 2.582 | 1.0273692 | European | European |
|  | 36.14206 | -121.00980 | 135 | 9.406 | 4.518 | 3.095 | 2.605 | 2.3304721 | European | European |
| 56 | 35.64635 | -120.69317 | 220 | 9.162 | 4.36 | 2.997 | 2.49 | 0.6870986 | European | European |
|  | 35.64635 | -120.69317 | 220 | 9.049 | 4.337 | 3.02 | 2.511 | 0.4733828 | European | European |
|  | 35.64635 | -120.69317 | 220 | 8.767 | 4.294 | 3.061 | 2.481 | -1.2149023 | African | European |
| 57 | 35.43319 | -120.29691 | 515 | Frayed | Frayed | no leg | no leg |  | no result | African |
| 58 | 35.39990 | -119.46532 | 82 | 8.901 | 4.179 | 3.028 | 2.515 | -0.1108658 | European | European |
|  | 35.39990 | -119.46532 | 82 | 9.054 | 4.359 | 3.212 | 2.623 | 0.3011962 | European | African |
|  | 35.39990 | -119.46532 | 82 | 9.064 | 4.437 | 3.244 | 2.661 | 0.7019294 | European | African |
| 59 | 35.38285 | -119.07032 | 120 | 9.075 | 4.119 | 3.164 | 2.523 | -1.0616566 | African | European |
|  | 35.38285 | -119.07032 | 120 | 9.103 | 4.341 | 3.223 | 2.57 | -0.5806047 | European | European |
|  | 35.38285 | -119.07032 | 120 | 8.632 | 4.142 | 3.025 | 2.482 | -1.3402076 | African | European |
| 60 | 34.83316 | -118.86647 | 1095 | 8.953 | 4.388 | 3.147 | 2.588 | 0.2015784 | European | African |
|  | 34.83316 | -118.86647 | 1095 | 9.308 | 4.339 | 3.002 | 2.441 | 0.17493 | European | African |
|  | 34.83316 | -118.86647 | 1095 | Frayed | Frayed | 3.142 | 2.554 |  | no result | European |
| 61 | 38.65731 | -121.52355 | 7 | 9.196 | 4.39 | 3.263 | 2.743 | 2.1150703 | European | European |
|  | 38.65731 | -121.52355 | 7 | 9.322 | 4.449 | 3.219 | 2.668 | 1.7460487 | European | European |
|  | 38.65731 | -121.52355 | 7 | Frayed | Frayed |  |  |  | no result | European |
| 62 | 39.14174 | -121.63221 | 18 | 8.992 | 4.292 | 3.156 | 2.617 | 0.5612443 | European | European |
|  | 39.14174 | -121.63221 | 18 | 9.042 | 4.257 | 3.076 | 2.595 | 1.13579 | European | European |
|  | 39.14174 | -121.63221 | 18 | 9.11 | 4.34 | 3.237 | 2.775 | 2.6373953 | European | European |
| 63 | 39.76076 | -121.84927 | 61 | 8.851 | 4.232 | 3.018 | 2.628 | 1.7809736 | European | European |
|  | 39.76076 | -121.84927 | 61 | 8.932 | 4.222 | 3.244 | 2.638 | -0.2675636 | European | European |
|  | 39.76076 | -121.84927 | 61 | 8.747 | 4.212 | 2.971 | 2.545 | 0.6382795 | European | European |
| 64 | 40.18607 | -122.20223 | 83 | 9.038 | 4.255 | 3.319 | 2.724 | 0.6472049 | European | European |
|  | 40.18607 | -122.20223 | 83 | 8.996 | 4.127 | 3.234 | 2.666 | 0.3419607 | European | European |
|  | 40.18607 | -122.20223 | 83 | 9.093 | 4.35 | 3.145 | 2.591 | 0.5779731 | European | European |
| 65 | 40.55856 | -122.35383 | 165 | 8.813 | 4.223 | 2.926 | 2.454 | -0.1909561 | European | European |
| 66 | 40.59282 | -122.37726 | 147 | 9.156 | 4.403 | 3.118 | 2.524 | -0.0069311 | European | European |
|  | 40.59282 | -122.37726 | 147 | 9.016 | 4.262 | 2.938 | 2.63 | 3.1161924 | European | European |
|  | 40.59282 | -122.37726 | 147 | 9.179 | 4.409 | 3.14 | 2.513 | -0.3555246 | European | European |
| 67 | 41.30355 | -122.30799 | 1075 | 8.887 | 4.308 | 3.027 | 2.402 | -1.8254694 | African | European |
|  | 41.30355 | -122.30799 | 1075 | 9.324 | 4.458 | 3.147 | 2.65 | 2.2335976 | European | European |
|  | 41.30355 | -122.30799 | 1075 | 9.026 | 4.143 | 3.188 | 2.677 | 1.1059276 | European | European |
| 68 | 41.73922 | -122.63371 | 790 | 9.178 | 4.44 | 3.163 | 2.651 | 1.6904913 | European | European |
|  | 41.73922 | -122.63371 | 790 | 9.403 | 4.343 | 3.233 | 2.659 | 1.5249982 | European | European |
|  | 41.73922 | -122.63371 | 790 | 9.102 | 4.351 | 2.984 | 2.564 | 1.8728829 | European | European |
| 69 | 42.18217 | -122.68570 | 627 | 9.237 | 4.417 | 3.315 | 2.674 | 0.5702917 | European | European |
|  | 42.18217 | -122.68570 | 627 | 9.366 | 4.526 | 3.224 | 2.743 | 3.1230091 | European | European |
|  | 42.18217 | -122.68570 | 627 | Frayed | Frayed | 3.233 | 2.61 |  | no result | European |
| 70 | 42.33123 | -122.86686 | 415 | 8.87 | 4.172 | 2.96 | 2.497 | 0.2316451 | European | European |
|  | 42.33123 | -122.86686 | 415 | 8.796 | 4.291 | 2.913 | 2.549 | 1.5398796 | European | European |
|  | 42.33123 | -122.86686 | 415 | 8.923 | 4.287 | 3.245 | 2.562 | -1.4639497 | African | European |
| 71 | 42.43759 | -123.31340 | 288 | 9.206 | 4.44 | 3.159 | 2.676 | 2.2120904 | European | European |
|  | 42.43759 | -123.31340 | 288 | 9.122 | 4.395 | 3.125 | 2.512 | -0.3727919 | European | European |
|  | 42.43759 | -123.31340 | 288 | 9.265 | 4.454 | 3.159 | 2.684 | 2.5083318 | European | European |
| 72 | 42.21922 | -123.64799 | 376 | 9.003 | 4.237 | 3.178 | 2.434 | -2.7028667 | African | European |
|  | 42.21922 | -123.64799 | 376 | Frayed | Frayed | 3.076 | 2.545 |  |  | European |
|  | 42.21922 | -123.64799 | 376 | 9.208 | 4.281 | 3.143 | 2.575 | 0.5432308 | European | European |
| 73 | 41.88900 | -123.80267 | 346 | 9.05 | 4.24 | 3.063 | 2.666 | 2.4339306 | European | European |
|  | 41.88900 | -123.80267 | 346 | 8.979 | 4.33 | 3.271 | 2.681 | 0.3976509 | European | European |
|  | 41.88900 | -123.80267 | 346 | 9.155 | 4.3 | 3.167 | 2.788 | 3.65896 | European | European |
| 74 | 41.80909 | -124.04837 | 66 | 9.456 | 4.408 | 3.181 | 2.683 | 2.6827057 | European | European |
|  | 41.80909 | -124.04837 | 66 | 9.085 | 4.373 | 3.18 | 2.616 | 0.6221591 | European | European |
|  | 41.80909 | -124.04837 | 66 | 8.987 | 4.273 | 3.144 | 2.655 | 1.2742556 | European | European |
| 75 | 41.74679 | -124.20082 | 6 | 9.229 | 4.289 | 3.283 | 2.745 | 1.8952814 | European | European |
|  | 41.74679 | -124.20082 | 6 | 9.365 | 4.356 | 3.118 | 2.695 | 3.2566561 | European | European |
|  | 41.74679 | -124.20082 | 6 | 9.24 | 4.153 | 3.184 | 2.633 | 0.980007 | European | European |
| 76 | 41.61253 | -124.10597 | 74 | 8.709 | 4.003 | 3.042 | 2.628 | 0.8899493 | European | European |
|  | 41.61253 | -124.10597 | 74 | 8.862 | 4.074 | 3.073 | 2.518 | -0.7662452 | European | European |
|  | 41.61253 | -124.10597 | 74 | 8.834 | 4.199 | 2.891 | 2.616 | 2.8526645 | European | European |
| 77 | 41.23907 | -124.08394 | 9 | 8.894 | 4.137 | 3.02 | 2.61 | 1.4582069 | European | European |
|  | 41.23907 | -124.08394 | 9 | 9.193 | 4.043 | 2.956 | 2.645 | 3.3490308 | European | European |
|  | 41.23907 | -124.08394 | 9 | 8.928 | 4.38 | 3.061 | 2.547 | 0.3735029 | European | European |
| 78 | 40.80693 | -124.14594 | 3 | 9.445 | 4.411 | 3.322 | 2.73 | 1.9272167 | European | European |
|  | 40.80693 | -124.14594 | 3 | 9.181 | 4.349 | 3.023 | 2.578 | 1.8832729 | European | European |
|  | 40.80693 | -124.14594 | 3 | 9.019 | 4.302 | 3.023 | 2.601 | 1.7943785 | European | European |
| 79 | 40.44757 | -124.04030 | 82 | 9.313 | 4.157 | 3.076 | 2.641 | 2.4479638 | European | European |
|  | 40.44757 | -124.04030 | 82 | 9.207 | 4.372 | 3.115 | 2.634 | 1.9134482 | European | European |
|  | 40.44757 | -124.04030 | 82 | 9.118 | 4.347 | 3.036 | 2.6 | 1.9436109 | European | European |
| 80 | 40.05448 | -123.79256 | 141 | 8.615 | 4.139 | 3.035 | 2.555 | -0.2998854 | European | European |
|  | 40.05448 | -123.79256 | 141 | 8.926 | 4.308 | 3.22 | 2.731 | 1.5971425 | European | European |
|  | 40.05448 | -123.79256 | 141 | 8.972 | 4.27 | 3.067 | 2.642 | 1.8393324 | European | European |
| 81 | 39.41246 | -123.80789 | 28 | 8.985 | 4.229 | 3.091 | 2.541 | -0.0837946 | European | European |
|  | 39.41246 | -123.80789 | 28 | 9.205 | 4.38 | 3.052 | 2.553 | 1.2643295 | European | European |
|  | 39.41246 | -123.80789 | 28 | 9.097 | 4.245 | 3.002 | 2.527 | 0.9352504 | European | European |
| 82 | 39.39342 | -123.44745 | 578 | 8.868 | 4.239 | 3.084 | 2.527 | -0.5204198 | European | European |
|  | 39.39342 | -123.44745 | 578 | 8.847 | 4.229 | 3.116 | 2.597 | 0.2183106 | European | European |
|  | 39.39342 | -123.44745 | 578 | 8.886 | 4.22 | 2.855 | 2.517 | 1.7738867 | European | European |
| 83 | 39.16892 | -122.95932 | 412 | 9.039 | 4.185 | 2.937 | 2.616 | 2.8622663 | European | European |
|  | 39.16892 | -122.95932 | 412 | 8.93 | 4.18 | 3.12 | 2.565 | -0.1979545 | European | European |
|  | 39.16892 | -122.95932 | 412 | 8.909 | Frayed | 3.112 | 2.489 |  | no result | European |
| 84 | 38.99424 | -122.54992 | 311 | 9.164 | 4.264 | 3.078 | 2.627 | 1.9530357 | European | European |
| 85 | 39.15275 | -122.23622 | 39 | 8.963 | 4.313 | 3.024 | 2.699 | 3.2579016 | European | European |
|  | 39.15275 | -122.23622 | 39 | 9.176 | 4.36 | 3.034 | 2.578 | 1.7670742 | European | European |
|  | 39.15275 | -122.23622 | 39 | 8.806 | 4.241 | 3.087 | 2.469 | -1.6538578 | African | European |
| 86 | 38.69534 | -122.03045 | 64 | 9.192 | 4.289 | 2.937 | 2.521 | 1.8210586 | European | European |
|  | 38.69534 | -122.03045 | 64 | Frayed | 4.317 | 3.122 | 2.624 |  | no result | European |
|  | 38.69534 | -122.03045 | 64 | 9.043 | 4.286 | 3.079 | 2.579 | 0.8801583 | European | European |
| 87 | 38.51956 | -121.97598 | 41 | 9.095 | 4.261 | 2.937 | 2.689 | 4.2886978 | European | European |
|  | 38.51956 | -121.97598 | 41 | 8.862 | 4.301 | 2.99 | 2.511 | 0.2781116 | European | European |
|  | 38.51956 | -121.97598 | 41 | 9.13 | 4.237 | 2.932 | 2.606 | 3.0442245 | European | European |
| 88 | 38.55313 | -121.74786 | 14 | Frayed | 4.219 | 3.078 | 2.452 |  | no result | European |
|  | 38.55313 | -121.74786 | 14 | 8.85 | 4.214 | 3.205 | 2.624 | -0.2976618 | European | European |
|  | 38.55313 | -121.74786 | 14 | 9.116 | 4.205 | 3.129 | 2.626 | 1.2017509 | European | European |
| 89 | 37.91913 | -122.10897 | 158 | 9.265 | 4.285 | 3.255 | 2.669 | 1.0366686 | European | European |
|  | 37.91913 | -122.10897 | 158 | 9.164 | 4.199 | 3.175 | 2.691 | 1.8883586 | European | European |
|  | 37.91913 | -122.10897 | 158 | 8.981 | 4.282 | 3.005 | 2.582 | 1.555344 | European | African |
| 90 | 38.02578 | -122.10173 | 1 | 9.259 | 4.32 | 3.03 | 2.705 | 4.0455171 | European | European |
|  | 38.02578 | -122.10173 | 1 | 9.126 | 4.265 | 3.07 | 2.663 | 2.5320936 | European | European |
|  | 38.02578 | -122.10173 | 1 | 9.187 | 4.36 | 3.216 | 2.591 | 0.0715461 | European | European |
| 91 | 38.31380 | -121.75595 | 4 | 9.33 | 4.401 | 3.291 | 2.694 | 1.3669949 | European | European |
|  | 38.31380 | -121.75595 | 4 | Frayed | Frayed | 3.147 | 2.807 |  |  | European |
|  | 38.31380 | -121.75595 | 4 | 9.318 | 4.471 | 3.213 | 2.694 | 2.2514879 | European | African |

**Table S2b**. Morphometric measurements, discriminant function scores and outcomes, and cytochrome b restriction results for honey bees collected in San Diego County: FWL, fore wing length; HWL, hind wing lenghth; TL, tibia length; FL, femur length (all in mm).

| Site | Latitude | Longitude | Alti-tude | FWL | HWL | TL | FL | Discriminant score | Discriminant function result | Cyt-b result |
| --- | --- | --- | --- | --- | --- | --- | --- | --- | --- | --- |
| 1 | 32.876784 | -117.248458 | 115 | 8.784 | 4.149 | 3.046 | 2.47 | -1.3686779 | African | European |
|  | 32.876784 | -117.248458 | 115 | Frayed | Frayed | 3.137 | 2.535 |  | no result | African |
|  | 32.876784 | -117.248458 | 115 | 8.944 | 4.15 | 3.296 | 2.552 | -2.2835382 | African | African |
| 2 | 32.938807 | -117.258201 | 14 | 9.095 | 4.162 | 3.346 | 2.553 | -2.4044071 | African | African |
|  | 32.938807 | -117.258201 | 14 | 8.516 | 4.099 | 2.998 | 2.345 | -3.6363468 | African | African |
|  | 32.938807 | -117.258201 | 14 | 8.628 | 4.096 | 3.113 | 2.281 | -5.6272613 | African | European |
| 3 | 32.93859 | -117.258136 | 13 | 8.878 | 4.272 | 3.059 | 2.48 | -0.9574044 | African | African |
|  | 32.93859 | -117.258136 | 13 | 8.786 | 4.283 | 3.322 | 2.543 | -2.9430354 | African | African |
|  | 32.93859 | -117.258136 | 13 | 9.201 | 4.269 | 3.422 | 2.414 | -5.0876751 | African | African |
| 4 | 33.04759 | -117.293885 | 19 | 8.692 | 4.24 | 3.324 | 2.502 | -3.9232318 | African | European |
|  | 33.04759 | -117.293885 | 19 | 8.987 | 4.279 | 3.36 | 2.241 | -7.7821132 | African | European |
|  | 33.04759 | -117.293885 | 19 | 9.193 | 4.257 | 3.342 | 2.563 | -1.836308 | African | African |
| 5 | 32.938807 | -117.258201 | 14 | 8.702 | 4.105 | 3.121 | 2.41 | -3.4068263 | African | African |
|  | 32.938807 | -117.258201 | 14 | 9.159 | 4.28 | 3.271 | 2.332 | -4.9142132 | African | African |
|  | 32.938807 | -117.258201 | 14 | 8.956 | 4.227 | 3.435 | 2.388 | -6.3184651 | African | African |
| 6 | 33.231234 | -117.317748 | 26 | Frayed | Frayed | 3.164 | 2.463 |  | no result | European |
|  | 33.231234 | -117.317748 | 26 | Frayed | 4.252 | 3.259 | 2.515 |  | no result | African |
|  | 33.231234 | -117.317748 | 26 | 9.109 | 4.245 | 3.268 | 2.507 | -2.1905004 | African | European |
| 7 | 33.258141 | -117.237060 | 70 | 9.156 | 4.212 | 3.354 | 2.549 | -2.3405721 | African | African |
|  | 33.258141 | -117.237060 | 70 | 9.254 | 4.395 | 3.292 | 2.654 | 0.5040615 | European | European |
|  | 33.258141 | -117.237060 | 70 | 9.116 | 4.327 | 3.174 | 2.66 | 1.4271813 | European | European |
| 8 | 33.361858 | -117.103829 | 109 | 8.823 | 4.255 | 3.228 | 2.663 | 0.0770406 | European | African |
|  | 33.361858 | -117.103829 | 109 | 8.667 | 4.086 | 3.246 | 2.428 | -4.5532622 | African | African |
|  | 33.361858 | -117.103829 | 109 | 8.969 | 4.171 | 3.323 | 2.486 | -3.5609529 | African | African |
| 9 | 33.346081 | -117.024321 | 219 | 9.117 | Blurry | 3.305 | 2.256 |  | no result | European |
|  | 33.346081 | -117.024321 | 219 | Frayed | Frayed | 3.213 | 2.305 |  | no result | African |
|  | 33.346081 | -117.024321 | 219 | 9.176 | 4.312 | 3.127 | 2.478 | -0.9147898 | African | European |
| 10 | 33.305764 | -116.869751 | 1443 | Blurry | 4.392 | 3.105 | 2.634 |  | no result | African |
|  | 33.305764 | -116.869751 | 1443 | 8.577 | 3.922 | 3.262 | 2.43 | -5.1166276 | African | African |
|  | 33.305764 | -116.869751 | 1443 | 9.12 | 4.406 | 3.319 | 2.758 | 1.5928432 | European | African |
| 11 | 32.942221 | -117.223124 | 36 | 9.297 | 4.406 | 3.166 | 2.623 | 1.4590663 | European | African |
|  | 32.942221 | -117.223124 | 36 | 9.366 | 4.152 | 3.163 | 2.448 | -1.5044164 | African | African |
|  | 32.942221 | -117.223124 | 36 | 9.309 | 4.561 | 3.475 | 2.495 | -3.700692 | African | African |
| 12 | 32.956412 | -117.108975 | 197 | 8.89 | Blurry | 3.187 | 2.49 |  | no result | European |
|  | 32.956412 | -117.108975 | 197 | 8.841 | 3.974 | 3.272 | 2.39 | -5.1491832 | African | African |
|  | 32.956412 | -117.108975 | 197 | 8.94 | 4.184 | 3.367 | 2.494 | -3.955337 | African | African |
| 13 | 32.939945 | -117.050897 | 215 | 9.078 | 4.258 | 3.281 | 2.613 | -0.6585115 | African | African |
|  | 32.939945 | -117.050897 | 215 | 8.603 | 3.903 | 2.998 | 2.04 | -8.6406259 | African | African |
|  | 32.939945 | -117.050897 | 215 | 9.112 | 4.39 | 3.368 | 2.613 | -1.3377523 | African | African |
| 14 | 32.930002 | -116.970101 | 362 | 8.838 | 4.235 | 3.204 | 2.619 | -0.3734086 | European | African |
|  | 32.930002 | -116.970101 | 362 | 8.899 | 4.171 | 3.055 | 2.614 | 1.2052591 | European | African |
|  | 32.930002 | -116.970101 | 362 | 9.117 | 4.35 | 3.2 | 2.547 | -0.6657229 | African | European |
| 15 | 32.994459 | -116.975378 | 541 | 9.018 | 4.408 | 3.223 | 2.43 | -3.0011794 | African | European |
|  | 32.994459 | -116.975378 | 541 | 9.207 | 4.448 | 3.384 | 2.49 | -3.2086414 | African | European |
|  | 32.994459 | -116.975378 | 541 | 9.207 | 4.222 | 3.286 | 2.283 | -5.8243333 | African | African |
| 16 | 33.016028 | -116.903691 | 433 | Blurry | 4.363 | 3.102 | 2.249 |  | no result | African |
|  | 33.016028 | -116.903691 | 433 | Frayed | 4.17 | 3.032 | 2.464 |  | no result | African |
|  | 33.016028 | -116.903691 | 433 | 9.522 | 4.545 | 3.457 | 2.734 | 0.9134797 | European | European |
| 17 | 33.106001 | -116.789635 | 693 | 9.262 | 4.393 | 3.164 | 2.723 | 3.0108468 | European | African |
|  | 33.106001 | -116.789635 | 693 | 9.114 | 4.264 | 3.05 | 2.499 | 0.0329933 | European | European |
|  | 33.106001 | -116.789635 | 693 | 9.158 | 4.343 | 3.386 | 2.647 | -0.9148934 | African | African |
| 18 | 33.047774 | -116.870501 | 441 | 8.782 | 3.733 | 3.121 | 2.57 | -1.0428051 | African | African |
|  | 33.047774 | -116.870501 | 441 | Blurry | Blurry | 3.258 | 2.551 |  | no result | European |
|  | 33.047774 | -116.870501 | 441 | 8.621 | 4.129 | 3.37 | 2.487 | -4.9712572 | African | European |
| 19 | 33.094486 | -116.960706 | 128 | 9.023 | Blurry | 3.176 | 2.591 |  | no result | European |
|  | 33.094486 | -116.960706 | 128 | Blurry | 4.403 | 3.191 | 2.521 |  | no result | European |
|  | 33.094486 | -116.960706 | 128 | 9.221 | 4.301 | 3.184 | 2.487 | -1.2740608 | African | European |
| 20 | 33.068312 | -117.063507 | 106 | Blurry | 4.278 | 3.171 | 2.876 |  | no result | African |
|  | 33.068312 | -117.063507 | 106 | 8.742 | 4.251 | 3.342 | 2.254 | -8.0287649 | African | African |
|  | 33.068312 | -117.063507 | 106 | 8.694 | 4.121 | 3.257 | 2.421 | -4.6741618 | African | African |
| 21 | 33.068962 | -117.119074 | 104 | 9.23 | 4.535 | 3.282 | 2.627 | 0.2789646 | European | European |
|  | 33.068962 | -117.119074 | 104 | 8.763 | 4.22 | 3.198 | 2.442 | -3.4094338 | African | European |
|  | 33.068962 | -117.119074 | 104 | 9.154 | 4.505 | 3.412 | 2.655 | -0.8737576 | African | African |
| 22 | 32.994317 | -117.212593 | 10 | 9.242 | 4.398 | 3.474 | 2.685 | -0.9515059 | African | African |
|  | 32.994317 | -117.212593 | 10 | 9.155 | 4.473 | 3.25 | 2.633 | 0.4532514 | European | European |
|  | 32.994317 | -117.212593 | 10 | 9.137 | 4.284 | 3.225 | 2.678 | 1.1795166 | European | African |
| 23 | 32.839024 | -117.044578 | 86 | 8.818 | 4.23 | 3.067 | 2.572 | 0.2590978 | European | African |
|  | 32.839024 | -117.044578 | 86 | 9.006 | 4.252 | 3.15 | 2.549 | -0.4997337 | European | European |
|  | 32.839024 | -117.044578 | 86 | 8.996 | 4.399 | 3.104 | 2.552 | 0.1921089 | European | African |
| 24 | 32.855621 | -116.939859 | 115 | 8.887 | Frayed | 3.19 | 2.653 |  | no result | African |
|  | 32.855621 | -116.939859 | 115 | 8.805 | 4.178 | 3.118 | 2.519 | -1.2454845 | African | African |
| 25 | 32.833602 | -116.865411 | 263 | 9.053 | 4.192 | 3.183 | 2.58 | -0.2987308 | European | European |
|  | 32.833602 | -116.865411 | 263 | 9.412 | 4.33 | 3.321 | 2.574 | -0.7933252 | African | European |
|  | 32.833602 | -116.865411 | 263 | 9.29 | 4.368 | 3.606 | 2.635 | -3.0882899 | African | European |
|  | 32.833602 | -116.865411 | 263 | 8.859 | 4.181 | 3.218 | 2.614 | -0.6168407 | European | African |
| 26 | 32.858339 | -116.819531 | 377 | 9.2 | 4.391 | 3.199 | 2.476 | -1.5567911 | African | African |
|  | 32.858339 | -116.819531 | 377 | 8.949 | 4.21 | 3.153 | 2.579 | -0.2358261 | European | African |
|  | 32.858339 | -116.819531 | 377 | 9.256 | 4.388 | 3.289 | 2.676 | 0.8920556 | European | African |
| 27 | 32.856349 | -116.809354 | 431 | 9.094 | 4.412 | 3.43 | 2.62 | -1.9012976 | African | European |
|  | 32.856349 | -116.809354 | 431 | 8.944 | 4.31 | 3.285 | 2.532 | -2.2988806 | African | African |
|  | 32.856349 | -116.809354 | 431 | 8.944 | 4.297 | 3.435 | 2.557 | -3.5014298 | African | European |
| 28 | 32.841553 | -116.540304 | 1134 | Frayed | 4.166 | 3.043 | 2.455 |  | no result | African |
|  | 32.841553 | -116.540304 | 1134 | 8.923 | 4.314 | 3.137 | 2.667 | 1.4336339 | European | African |
|  | 32.841553 | -116.540304 | 1134 | Frayed | 4.044 | 3.211 | 2.528 |  | no result | African |
| 29 | 32.807893 | -116.640025 | 969 | 8.942 | 4.371 | 3.219 | 2.406 | -3.5871253 | African | African |
|  | 32.807893 | -116.640025 | 969 | 9.052 | 4.354 | 3.317 | 2.498 | -2.8696416 | African | African |
| 30 | 32.764644 | -116.687290 | 760 | 8.781 | 4.245 | 3.025 | 2.33 | -3.3242991 | African | European |
| 31 | 32.798116 | -116.747611 | 428 | 8.907 | 4.272 | 3.288 | 2.479 | -3.3363251 | African | European |
|  | 32.798116 | -116.747611 | 428 | 8.779 | 4.215 | 3.042 | 2.542 | -0.0817073 | European | European |
|  | 32.798116 | -116.747611 | 428 | 9.097 | 4.31 | 3.372 | 2.411 | -4.8167805 | African | African |
| 32 | 32.793394 | -116.805916 | 456 | 8.715 | 4.208 | 3.19 | 2.427 | -3.7048855 | African | African |
|  | 32.793394 | -116.805916 | 456 | 8.761 | 4.05 | 3.025 | 2.473 | -1.2745499 | African | African |
|  | 32.793394 | -116.805916 | 456 | Frayed | Frayed | 3.213 | 2.453 |  | no result | African |
| 33 | 32.780344 | -116.872045 | 144 | 8.843 | Frayed | 3.07 | 2.572 |  | no result | African |
|  | 32.780344 | -116.872045 | 144 | 8.822 | 4.269 | 3.165 | 2.534 | -1.3467735 | African | African |
|  | 32.780344 | -116.872045 | 144 | 8.837 | 4.24 | 3.423 | 2.46 | -5.297722 | African | African |
| 34 | 32.795368 | -116.943229 | 143 | 9.148 | 4.236 | 3.227 | 2.567 | -0.6866103 | African | European |
|  | 32.795368 | -116.943229 | 143 | 8.948 | 4.25 | 3.341 | 2.515 | -3.2352089 | African | African |
|  | 32.795368 | -116.943229 | 143 | 8.899 | 4.464 | 3.232 | 2.513 | -1.9717173 | African | African |
| 35 | 32.772696 | -117.021148 | 158 | 9.16 | 4.378 | 3.366 | 2.631 | -0.9160945 | African | African |
|  | 32.772696 | -117.021148 | 158 | 9.138 | 4.487 | 3.266 | 2.467 | -2.4557618 | African | African |
|  | 32.772696 | -117.021148 | 158 |  | 4.351 | 3.141 | 2.614 |  | no result | African |
| 36 | 32.767061 | -117.150974 | 14 | 9.084 | 4.375 | 3.063 | 2.526 | 0.3954887 | European | European |
|  | 32.767061 | -117.150974 | 14 | 8.932 | 4.268 | 3.219 | 2.537 | -1.5964761 | African | African |
|  | 32.767061 | -117.150974 | 14 | 9.122 | 4.327 | 3.351 | 2.667 | -0.3258142 | European | African |
| 37 | 32.7436393 | -115.994334 | 114 | 9.049 | 4.213 | 3.076 | 2.389 | -2.2669382 | African | African |
|  | 32.7436393 | -115.994334 | 114 | 9.059 | 4.345 | 3.247 | 2.63 | 0.0389169 | European | African |
|  | 32.7436393 | -115.994334 | 114 | Frayed | Frayed | 3.201 | 2.577 |  | no result | African |
| 38 | 32.7938948 | -116.108216 | 288 | 9.087 | 4.333 | 3.316 | 2.462 | -3.3848463 | African | African |
|  | 32.7938948 | -116.108216 | 288 | Frayed | 4.285 | 3.194 | 2.343 |  | no result | African |
|  | 32.7938948 | -116.108216 | 288 | 8.736 | 4.215 | 3.204 | 2.405 | -4.151994 | African | African |
| 39 | 32.8269748 | -116.165554 | 379 | 9.02 | 4.191 | 3.365 | 2.409 | -5.113474 | African | African |
|  | 32.8269748 | -116.165554 | 379 | 8.846 | 4.275 | 3.133 | 2.56 | -0.5138039 | European | African |
|  | 32.8269748 | -116.165554 | 379 | 9.068 | 4.342 | 3.268 | 2.376 | -4.3167814 | African | European |
| 40 | 32.8717995 | -116.209247 | 217 | 8.799 | 4.304 | 3.342 | 2.522 | -3.4407222 | African | African |
|  | 32.8717995 | -116.209247 | 217 | 8.983 | Frayed | 3.274 | 2.499 |  | no result | European |
|  | 32.8717995 | -116.209247 | 217 | 8.862 | 4.21 | 3.151 | 2.38 | -3.6859178 | African | African |
| 41 | 32.911198 | -116.235802 | 297 | 9.102 | 4.382 | 3.313 | 2.51 | -2.4711072 | African | African |
|  | 32.911198 | -116.235802 | 297 | 9.139 | 4.48 | 3.301 | 2.389 | -4.1088269 | African | African |
|  | 32.911198 | -116.235802 | 297 | 9.051 | 4.308 | 3.199 | 2.501 | -1.6240569 | African | African |
| 42 | 32.9509709 | -116.287889 | 359 | 8.809 | 4.202 | 3.012 | 2.479 | -0.7326197 | African | African |
|  | 32.9509709 | -116.287889 | 359 | 8.89 | 4.186 | 3.284 | 2.387 | -4.9447677 | African | African |
|  | 32.9509709 | -116.287889 | 359 | 8.848 | 4.151 | 3.091 | 2.49 | -1.3569205 | African | African |
| 43 | 32.9755287 | -116.350329 | 467 | 8.988 | 4.397 | 3.205 | 2.446 | -2.6371031 | African | African |
|  | 32.9755287 | -116.350329 | 467 | Frayed | 4.268 | 3.167 | 2.461 |  | no result | European |
|  | 32.9755287 | -116.350329 | 467 | Frayed | Frayed | 3.264 | 2.514 |  | no result | European |
| 44 | 32.978978 | -116.423139 | 611 | 8.453 | 3.795 | 3.133 | 2.403 | -4.6523734 | African | African |
|  | 32.978978 | -116.423139 | 611 | 8.982 | 4.24 | 3.361 | 2.322 | -6.528895 | African | African |
|  | 32.978978 | -116.423139 | 611 | 8.89 | 4.245 | 3.216 | 2.578 | -1.0281239 | African | European |
| 45 | 33.1174759 | -116.437025 | 595 | Frayed | 4.182 | 3.138 | 2.602 |  | no result | European |
|  | 33.1174759 | -116.437025 | 595 | 9.099 | 4.462 | 3.323 | 2.61 | -0.8533504 | African | European |
|  | 33.1174759 | -116.437025 | 595 | 8.942 | 4.292 | 3.016 | 2.505 | 0.0938915 | European | African |
| 46 | 33.1501785 | -116.349281 | 513 | Frayed | Frayed | 3.176 | 2.387 |  | no result | African |
|  | 33.1501785 | -116.349281 | 513 | Frayed | 4.212 | 3.206 | 2.272 |  | no result | African |
|  | 33.1501785 | -116.349281 | 513 | Frayed | 4.196 | 3.335 | 2.471 |  | no result | African |
| 47 | 33.2119256 | -116.365679 | 211 | Frayed | Frayed | 3.165 | 2.465 |  | no result | European |
|  | 33.2119256 | -116.365679 | 211 | 8.768 | Frayed | 3.282 | 2.545 |  | no result | African |
|  | 33.2119256 | -116.365679 | 211 | 9.113 | Blurry | 3.311 | 2.606 |  | no result | African |
| 48 | 33.2505283 | -116.405511 | 265 | 8.93 | 4.173 | 3.065 | 2.504 | -0.6184857 | European | European |
|  | 33.2505283 | -116.405511 | 265 | 9.012 | 4.144 | 3.045 | 2.37 | -2.4247726 | African | African |
|  | 33.2505283 | -116.405511 | 265 | Frayed | Frayed | 3.071 | 2.494 |  | no result | European |
| 49 | 32.8532228 | -117.256651 | 4 | 9.049 | 4.414 | 3.267 | 2.582 | -0.8995692 | African | African |
|  | 32.8532228 | -117.256651 | 4 | 9.036 | 4.291 | 3.119 | 2.506 | -0.7499057 | African | African |
|  | 32.8532228 | -117.256651 | 4 | 8.922 | 4.272 | 3.073 | 2.413 | -2.0906225 | African | European |
| 50 | 32.8300494 | -117.276886 | 23 | 8.81 | 4.169 | 3.026 | 2.455 | -1.31138 | African | European |
|  | 32.8300494 | -117.276886 | 23 | 8.765 | 4.2 | 3.007 | 2.558 | 0.498573 | European | European |
|  | 32.8300494 | -117.276886 | 23 | 8.865 | 4.262 | 3.146 | 2.497 | -1.6497275 | African | European |
| 51 | 32.7966361 | -117.253603 | 8 | 8.918 | 4.205 | 3.402 | 2.539 | -3.6219344 | African | African |
|  | 32.7966361 | -117.253603 | 8 | 8.801 | 4.195 | 3.136 | 2.556 | -0.8215963 | African | African |
|  | 32.7966361 | -117.253603 | 8 | 8.753 | Frayed | 3.197 | 2.543 |  | no result | African |
| 52 | 32.7627932 | -117.232130 | 3 | 8.789 | 4.016 | 3.16 | 2.578 | -0.9651278 | African | African |
|  | 32.7627932 | -117.232130 | 3 | 9.283 | 4.42 | 3.02 | 2.582 | 2.323557 | European | European |
|  | 32.7627932 | -117.232130 | 3 | 8.844 | 4.157 | 3.117 | 2.648 | 0.9461199 | European | African |
| 53 | 32.7279406 | -117.151530 | 76 | 8.724 | 4.269 | 3.193 | 2.584 | -1.0739825 | African | European |
|  | 32.7279406 | -117.151530 | 76 | 9.004 | 4.158 | 3.117 | 2.462 | -1.6900056 | African | European |
|  | 32.7279406 | -117.151530 | 76 | Frayed | Frayed | 3.167 | 2.438 |  | no result | European |
| 54 | 32.7026445 | -117.145749 | 15 | 9.032 | 4.263 | 3.079 | 2.451 | -1.267507 | African | European |
|  | 32.7026445 | -117.145749 | 15 | 8.666 | 4.011 | 3.086 | 2.347 | -4.269131 | African | African |
|  | 32.7026445 | -117.145749 | 15 | Frayed | Frayed | 3.22 | 2.422 |  | no result | European |
| 55 | 32.5766379 | -117.084388 | 9 | 8.82 | 4.335 | 3.257 | 2.38 | -4.7669927 | African | African |
|  | 32.5766379 | -117.084388 | 9 | Frayed | Frayed | 3.128 | 2.421 |  | no result | African |
|  | 32.5766379 | -117.084388 | 9 | 8.939 | 4.203 | 3.281 | 2.58 | -1.6145143 | African | African |
| 56 | 32.563875 | -116.966885 | 149 | 8.759 | 4.15 | 3.102 | 2.399 | -3.1863825 | African | European |
|  | 32.563875 | -116.966885 | 149 | 8.972 | 4.32 | 3.415 | 2.482 | -4.4160854 | African | European |
|  | 32.563875 | -116.966885 | 149 | 8.795 | 4.307 | 3.138 | 2.463 | -2.2417678 | African | African |
| 57 | 32.6273939 | -116.963661 | 195 | 9.033 | 4.231 | 3.13 | 2.601 | 0.60527 | European | African |
|  | 32.6273939 | -116.963661 | 195 | 8.763 | 4.203 | 3.293 | 2.504 | -3.4271643 | African | African |
|  | 32.6273939 | -116.963661 | 195 | 9.171 | 4.291 | 3.28 | 2.408 | -3.7242255 | African | African |
| 58 | 32.6340543 | -116.917212 | 156 | 8.76 | 4.274 | 3.25 | 2.437 | -3.9860951 | African | European |
|  | 32.6340543 | -116.917212 | 156 | 8.981 | 4.264 | 3.071 | 2.485 | -0.7538501 | African | European |
|  | 32.6340543 | -116.917212 | 156 | 8.703 | 4.192 | 3.054 | 2.483 | -1.3928367 | African | European |
| 59 | 32.6523514 | -116.854942 | 194 | 8.829 | 4.25 | 3.13 | 2.366 | -3.72579 | African | African |
|  | 32.6523514 | -116.854942 | 194 | 9.003 | 4.226 | 3.206 | 2.521 | -1.5921191 | African | African |
|  | 32.6523514 | -116.854942 | 194 | 8.662 | 4.218 | 3.065 | 2.46 | -1.957297 | African | African |
| 60 | 32.6408321 | -116.772593 | 348 | 9.136 | 4.425 | 3.111 | 2.246 | -4.4996643 | African | European |
|  | 32.6408321 | -116.772593 | 348 |  |  |  |  |  | no result | not apis |
|  | 32.6408321 | -116.772593 | 348 | 8.785 | 4.279 | 3.072 | 2.189 | -6.077256 | African | European |
| 61 | 32.6140925 | -116.714561 | 288 | 8.952 | 4.298 | 3.213 | 2.398 | -3.7176596 | African | European |
|  | 32.6140925 | -116.714561 | 288 | 9.274 | 4.473 | 3.208 | 2.554 | -0.0917699 | European | African |
|  | 32.6140925 | -116.714561 | 288 | 9.106 | 4.374 | 3.128 | 2.501 | -0.6502779 | African | European |
| 62 | 32.5961128 | -116.652397 | 517 | 8.638 | 4.077 | 3.117 | 2.449 | -2.9219666 | African | African |
|  | 32.5961128 | -116.652397 | 517 | 8.916 | 4.235 | 3.228 | 2.518 | -2.0831177 | African | African |
|  | 32.5961128 | -116.652397 | 517 | 9.009 | 4.275 | 3.226 | 2.511 | -1.8935926 | African | European |
| 63 | 32.6109821 | -116.636470 | 741 | 9.724 | 4.564 | 3.355 | 2.615 | 0.5848017 | European | African |
|  | 32.6109821 | -116.636470 | 741 | 9.095 | 4.297 | 3.237 | 2.355 | -4.3170724 | African | European |
|  | 32.6109821 | -116.636470 | 741 | 8.944 | 4.13 | 3.18 | 2.438 | -2.9373312 | African | European |
| 64 | 32.5908726 | -116.524388 | 676 | 8.912 | 4.257 | 3.078 | 2.467 | -1.3046324 | African | European |
|  | 32.5908726 | -116.524388 | 676 | Frayed | Frayed | 3.087 | 2.393 |  | no result | African |
|  | 32.5908726 | -116.524388 | 676 | 9.036 | 4.335 | 3.113 | 2.477 | -1.1065656 | African | African |
| 65 | 32.6309694 | -116.436177 | 826 | 8.883 | 4.136 | 3.129 | 2.488 | -1.7239256 | African | African |
|  | 32.6309694 | -116.436177 | 826 | Frayed | Frayed | 3.183 | 2.395 |  | no result | African |
|  | 32.6309694 | -116.436177 | 826 | 9.187 | 4.371 | 3.278 | 2.449 | -2.89532 | African | European |
| 66 | 32.6524828 | -116.396316 | 953 | Frayed | 4.255 | 3.114 | 2.497 |  | no result | African |
|  | 32.6524828 | -116.396316 | 953 | 8.717 | 4.076 | 3.138 | 2.493 | -2.2286029 | African | African |
|  | 32.6524828 | -116.396316 | 953 | 9.198 | 4.26 | 3.244 | 2.541 | -1.1373553 | African | African |
| 67 | 32.6693213 | -116.307193 | 1145 | Frayed | 4.321 | 3.127 | 2.413 |  | no result | European |
|  | 32.6693213 | -116.307193 | 1145 | Frayed | 4.218 | 3.212 | 2.434 |  | no result | European |
|  | 32.6693213 | -116.307193 | 1145 | 9.166 | 4.373 | 3.021 | 2.51 | 0.7845945 | European | African |
| 68 | 33.1511736 | -117.039034 | 221 | 8.841 | 4.2 | 3.212 | 2.481 | -2.7489589 | African | European |
|  | 33.1511736 | -117.039034 | 221 | 9.306 | 4.375 | 3.209 | 2.599 | 0.5944366 | European | European |
|  | 33.1511736 | -117.039034 | 221 | 9.169 | 4.467 | 3.083 | 2.563 | 1.1132578 | European | African |
| 69 | 33.1224 | -116.237600 | 324 | 8.738 | 4.243 | 3.126 | 2.641 | 0.5738212 | European | African |
|  | 33.1224 | -116.237600 | 324 | 8.616 | 4.194 | 2.995 | 2.534 | -0.1482924 | European | African |
|  | 33.1224 | -116.237600 | 324 | 8.079 | 4.222 | 3.103 | 2.657 | -0.6038991 | European | European |
| 70 | 33.1338 | -116.366500 | 423 | 8.98 | 4.064 | 3.121 | 2.562 | -0.2728462 | European | African |
|  | 33.1338 | -116.366500 | 423 | 8.759 | 4.162 | 2.989 | 2.496 | -0.3846106 | European | European |
|  | 33.1338 | -116.366500 | 423 | 8.88 | 4.106 | 3.075 | 2.418 | -2.3377024 | African | African |
| 71 | 33.1324 | -116.344800 | 386 | 9.01 | 4.259 | 3.132 | 2.545 | -0.3550916 | European | African |
|  | 33.1324 | -116.344800 | 386 | 8.995 | 4.18 | 3.002 | 2.467 | -0.3810899 | European | African |
|  | 33.1324 | -116.344800 | 386 | 8.939 | 4.305 | 3.026 | 2.484 | -0.3474289 | European | African |
| 72 | 33.038 | -116.408800 | 775 | 8.945 | 4.267 | 3.085 | 2.491 | -0.8916278 | African | African |
|  | 33.038 | -116.408800 | 775 | 8.679 | 4.178 | 3.253 | 2.613 | -1.4620303 | African | African |
|  | 33.038 | -116.408800 | 775 | 8.77 | 4.168 | 3.18 | 2.57 | -1.1715858 | African | African |
| 73 | 32.8673 | -116.610600 | 1086 | 9.085 | 4.231 | 3.046 | 2.623 | 1.989079 | European | European |
|  | 32.8673 | -116.610600 | 1086 | 8.778 | 4.184 | 3.022 | 2.516 | -0.334146 | European | European |
|  | 32.8673 | -116.610600 | 1086 | 9.167 | 4.442 | 3.055 | 2.549 | 1.1468097 | European | European |
| 74 | 32.9194 | -116.572500 | 1244 | 8.939 | 4.336 | 3.049 | 2.549 | 0.5079987 | European | African |
|  | 32.9194 | -116.572500 | 1244 | 9 | 4.229 | 3.242 | 2.535 | -1.7500876 | African | African |
|  | 32.9194 | -116.572500 | 1244 | 9.098 | 4.352 | 3.167 | 2.67 | 1.6501718 | European | European |
| 75 | 32.7384 | -116.910000 | 151 | 8.767 | 4.125 | 3.084 | 2.525 | -0.9458766 | African | African |
|  | 32.7384 | -116.910000 | 151 | 9.044 | 4.399 | 3.188 | 2.66 | 1.1846469 | European | African |
|  | 32.7384 | -116.910000 | 151 | 8.956 | 4.226 | 3.145 | 2.596 | 0.1641742 | European | African |
| 76 | 33.1367 | -116.380000 | 442 | Frayed | Frayed | 3.002 | 2.589 |  | no result | European |
|  | 33.1367 | -116.380000 | 442 | 8.574 | 4.167 | 2.992 | 2.468 | -1.3336011 | African | African |
|  | 33.1367 | -116.380000 | 442 | 8.551 | 4.261 | 3.073 | 2.46 | -2.2694185 | African | African |
| 77 | 33.1536 | -116.547500 | 827 | 8.931 | Frayed | 3.219 | 2.711 |  | no result | African |
|  | 33.1536 | -116.547500 | 827 | Frayed | Frayed | 3.133 | 2.624 |  | no result | African |
|  | 33.1536 | -116.547500 | 827 | 8.613 | Frayed | 3.111 | 2.604 |  | no result | African |
| 78 | 33.2101 | -116.489400 | 1241 | 9.142 | 4.25 | 3.058 | 2.609 | 1.7991741 | European | European |
|  | 33.2101 | -116.489400 | 1241 | 9.005 | 4.284 | 3.095 | 2.581 | 0.6446215 | European | African |
|  | 33.2101 | -116.489400 | 1241 | 8.793 | 4.199 | 2.955 | 2.496 | 0.1075457 | European | African |
| 79 | 33.2762 | -116.425900 | 317 | 8.781 | 4.35 | 3.149 | 2.643 | 0.6001967 | European | African |
|  | 33.2762 | -116.425900 | 317 | 8.338 | 4.045 | 2.92 | 2.414 | -2.1926187 | African | African |
|  | 33.2762 | -116.425900 | 317 | 8.57 | 4.195 | 3.094 | 2.547 | -1.1032846 | African | African |
| 80 | 32.905 | -116.572000 | 1290 | 8.864 | 4.05 | 3.113 | 2.675 | 1.3501743 | European | European |
|  | 32.905 | -116.572000 | 1290 | 8.617 | 4.244 | 3.001 | 2.662 | 1.9432246 | European | European |
|  | 32.905 | -116.572000 | 1290 | 9.187 | 4.168 | 3.138 | 2.624 | 1.2070188 | European | European |
| 81 | 32.9605 | -116.581280 | 1476 | 8.59 | 3.913 | 2.996 | 2.53 | -0.6313979 | European | European |
|  | 32.9605 | -116.581280 | 1476 | 8.976 | 4.345 | 3.034 | 2.544 | 0.6898631 | European | European |
|  | 32.9605 | -116.581280 | 1476 | 8.963 | 4.227 | 3.031 | 2.603 | 1.5098706 | European | African |
| 82 | 33.3348 | -116.919700 | 1654 | 8.899 | 4.081 | 3.045 | 2.488 | -0.8571473 | African | African |
|  | 33.3348 | -116.919700 | 1654 | 8.756 | 4.231 | 3.118 | 2.58 | -0.3073675 | European | African |
|  | 33.3348 | -116.919700 | 1654 | 9.401 | 4.324 | 3.253 | 2.744 | 2.6733828 | European | African |
| 83 | 32.902 | -116.552000 | 1440 | 8.7 | 4.327 | 3.189 | 2.628 | -0.3021799 | European | African |
|  | 32.902 | -116.552000 | 1440 | 8.93 | 4.172 | 3.124 | 2.6 | 0.3218124 | European | African |
|  | 32.902 | -116.552000 | 1440 | 8.84 | 4.24 | 3.035 | 2.534 | 0.0458886 | European | African |
| 84 | 32.799973 | -117.137015 | 116 | Frayed | 4.091 | 3.114 | 2.49 |  | no result | European |
|  | 32.799973 | -117.137015 | 116 | 9.16 | 4.275 | 3.091 | 2.663 | 2.4064626 | European | African |
|  | 32.799973 | -117.137015 | 116 | 9.061 | 4.501 | 3.038 | 2.62 | 2.2930315 | European | European |
| 85 | 32.8753 | -117.248300 | 107 | 8.714 | Frayed | 3.232 | 2.549 |  | no result | European |
|  | 32.8753 | -117.248300 | 107 | Frayed | Frayed | 2.889 | 2.295 |  | no result | African |
|  | 32.8753 | -117.248300 | 107 | 8.825 | 4.198 | 3.113 | 2.612 | 0.4023222 | Eruopean | European |
| 86 | 32.8914 | -117.091700 | 231 | Frayed | Frayed | 3.064 | 2.644 |  | no result | African |
|  | 32.8914 | -117.091700 | 231 | 9.141 | 4.337 | 3.131 | 2.656 | 1.8942055 | European | African |
|  | 32.8914 | -117.091700 | 231 | 8.751 | 4.124 | 3.075 | 2.521 | -0.9570101 | African | African |
| 87 | 33.0688 | -116.116200 | 70 | Frayed | Frayed | 3.113 | 2.61 |  | no result | African |
|  | 33.0688 | -116.116200 | 70 | 8.789 | 4.256 | 3.167 | 2.546 | -1.2707658 | African | African |
|  | 33.0688 | -116.116200 | 70 | 8.784 | 4.167 | 3.144 | 2.605 | -0.182654 | European | African |
| 88 | 33.1224 | -116.237600 | 324 | Frayed | Frayed | 3.009 | 2.598 |  | no result | African |
|  | 33.1224 | -116.237600 | 324 | 8.626 | 4.167 | 3.113 | 2.598 | -0.3649489 | European | African |
|  | 33.1224 | -116.237600 | 324 | 8.741 | 4.058 | 3.03 | 2.572 | 0.2497174 | European | African |
| 89 | 33.1338 | -116.366500 | 423 | 8.806 | Frayed | 3.154 | 2.531 |  | no result | African |
|  | 33.1338 | -116.366500 | 423 | 8.835 | 4.389 | 3.171 | 2.582 | -0.4474587 | European | African |
|  | 33.1338 | -116.366500 | 423 | 8.996 | 4.285 | 2.935 | 2.585 | 2.3902614 | European | European |
| 90 | 33.0688 | -116.116200 | 70 | Frayed | Frayed | 3.002 | 2.548 |  | no result | African |
|  | 33.0688 | -116.116200 | 70 | 8.928 | 4.12 | 3.036 | 2.558 | 0.5030418 | European | African |
|  | 33.0688 | -116.116200 | 70 | 8.885 | 4.15 | 3.208 | 2.597 | -0.7605975 | African | European |
| 91 | 32.879 | -117.240400 | 128 | Frayed | Frayed | 3.121 | 2.469 |  | no result | African |
|  | 32.879 | -117.240400 | 128 | Frayed | 4.272 | 3.017 | 2.523 |  | no result | African |
|  | 32.879 | -117.240400 | 128 | Frayed | Frayed | 3.05 | 2.442 |  | no result | European |
| 92 | 32.9922 | -117.114000 | 240 | Frayed | Frayed | 3.032 | 2.541 |  | no result | African |
|  | 32.9922 | -117.114000 | 240 | Frayed | Frayed | 3.011 | 2.528 |  | no result | European |
|  | 32.9922 | -117.114000 | 240 | 8.916 | 4.326 | 3.011 | 2.594 | 1.5775908 | European | Blank |
| 93 | 33.1324 | -116.344800 | 386 | Frayed | 4.365 | 3.164 | 2.513 |  | no result | African |
|  | 33.1324 | -116.344800 | 386 | 8.885 | 4.365 | 3.093 | 2.453 | -1.6296066 | African | European |
|  | 33.1324 | -116.344800 | 386 | 8.983 | 4.271 | 3.157 | 2.492 | -1.5405603 | African | European |
| 94 | 33.1369 | -116.379600 | 438 | Frayed | Frayed | 3.123 | 2.511 |  | no result | European |
|  | 33.1369 | -116.379600 | 438 | 8.984 | 4.298 | 3.038 | 2.591 | 1.3784679 | European | European |
|  | 33.1369 | -116.379600 | 438 | 8.744 | 4.333 | 3.013 | 2.472 | -0.8619457 | African | African |
| 95 | 33.1559 | -116.344900 | 479 | Frayed | Frayed | 2.975 | 2.231 |  | no result | African |
|  | 33.1559 | -116.344900 | 479 | Frayed | Frayed | 3.031 | 2.427 |  | no result | European |
|  | 33.1559 | -116.344900 | 479 | 8.875 | 4.081 | 3.191 | 2.587 | -0.8522924 | African | European |
| 96 | 33.108 | -116.497600 | 732 | Frayed | Frayed | 2.975 | 2.512 |  | no result | European |
|  | 33.108 | -116.497600 | 732 | 8.429 | 4.02 | 2.945 | 2.516 | -0.592836 | European | African |
|  | 33.108 | -116.497600 | 732 | 8.897 | 4.219 | 3.209 | 2.559 | -1.2782074 | African | African |
| 97 | 32.7216 | -117.064700 | 116 | 8.917 | 4.498 | 3.038 | 2.577 | 1.2242345 | European | African |
|  | 32.7216 | -117.064700 | 116 | 8.596 | 4.141 | 2.96 | 2.459 | -1.1166096 | African | African |
|  | 32.7216 | -117.064700 | 116 | 8.954 | 4.133 | 3.088 | 2.465 | -1.488759 | African | African |
| 98 | 32.7465 | -117.179500 | 55 | 8.999 | 4.378 | 3.207 | 2.613 | 0.0756353 | European | European |
|  | 32.7465 | -117.179500 | 55 | 8.942 | 4.203 | 3.205 | 2.638 | 0.1492867 | European | African |
|  | 32.7465 | -117.179500 | 55 | 9.017 | 4.312 | 3.16 | 2.688 | 1.7663468 | European | European |
| 99 | 32.6689 | -117.108400 | 6 | 9.011 | 4.317 | 3.113 | 2.633 | 1.3582866 | European | European |
|  | 32.6689 | -117.108400 | 6 | 9.056 | 4.297 | 3.237 | 2.597 | -0.4599882 | European | African |
|  | 32.6689 | -117.108400 | 6 | 8.785 | 4.263 | 2.928 | 2.615 | 2.3973174 | European | African |
| 100 | 32.7829 | -116.997200 | 201 | Frayed | 4.126 | 2.937 | 2.472 |  | no result | European |
|  | 32.7829 | -116.997200 | 201 | 8.916 | 4.14 | 3.104 | 2.665 | 1.5227395 | European | African |
|  | 32.7829 | -116.997200 | 201 | 9.002 | 4.305 | 2.999 | 2.576 | 1.6019043 | European | African |
| 101 | 32.8547 | -117.207900 | 115 | 8.649 | 4.098 | 3.011 | 2.599 | 0.7102063 | European | African |
|  | 32.8547 | -117.207900 | 115 | 8.786 | 4.357 | 3.151 | 2.583 | -0.3806152 | European | African |
|  | 32.8547 | -117.207900 | 115 | 9.101 | 4.284 | 3.082 | 2.548 | 0.48511 | European | African |
