# Supplementary material for: Range and Frequency of Africanized Honey Bees in California (USA)
Source: PLoS One. 2015 Sep 11;10(9):e0137407. doi: 10.1371/journal.pone.0137407 (PMC4567290; doi:10.1371/journal.pone.0137407)
Supplement: S3 Table — (DOCX) [file pone.0137407.s003.docx]

**Table S3a**. Morphometric measurements, discriminant function scores and outcomes, and cytochrome b restriction results for honey bees collected from hives of 11 San Diego beekeepers: FWL, fore wing length; HWL, hind wing lenghth; TL, tibia length; FL, femur length (all in mm).

| Keeper | Hive | Bee | FWL | HWL | TL | FL | Ave. FWL | Ave. HWL | Ave. TL | Ave. FL | Disc. score | Disc. result | Cyt-b result |
| --- | --- | --- | --- | --- | --- | --- | --- | --- | --- | --- | --- | --- | --- |
| 1 | 1 | 1 | 9.077 | 4.325 | 3.174 | 2.605 | 9.1778 | 4.3408 | 3.1703 | 2.5841 | 0.4874 | European | European |
|  |  | 2 | 9.206 | 4.625 | 3.29 | 2.725 |  |  |  |  |  |  |  |
|  |  | 3 | 9.303 | 4.374 | 3.19 | 2.521 |  |  |  |  |  |  |  |
|  |  | 4 | 9.628 | 4.58 | 3.171 | 2.465 |  |  |  |  |  |  |  |
|  |  | 5 | 8.862 | 4.167 | 3.046 | 2.57 |  |  |  |  |  |  |  |
|  |  | 6 | 9.281 | 4.251 | 3.218 | 2.651 |  |  |  |  |  |  |  |
|  |  | 7 | 9.334 | 4.316 | 3.254 | 2.669 |  |  |  |  |  |  |  |
|  |  | 8 | 9.008 | 4.301 | 3.146 | 2.656 |  |  |  |  |  |  |  |
|  |  | 9 | 9.075 | 4.265 | 3.091 | 2.477 |  |  |  |  |  |  |  |
|  |  | 10 | 9.004 | 4.204 | 3.123 | 2.502 |  |  |  |  |  |  |  |
| 2 | 1 | 1 | 9.276 | 4.336 | 3.248 | 2.549 | 9.0348 | 4.3301 | 3.1941 | 2.5733 | -0.7020 | European | European |
|  |  | 2 | 8.842 | 4.27 | 3.144 | 2.503 |  |  |  |  |  |  |  |
|  |  | 3 | 8.68 | 4.241 | 3.073 | 2.508 |  |  |  |  |  |  |  |
|  |  | 4 | 9.025 | 4.221 | 3.144 | 2.544 |  |  |  |  |  |  |  |
|  |  | 5 | 9.048 | 4.224 | 3.168 | 2.543 |  |  |  |  |  |  |  |
|  |  | 6 | 9.079 | 4.413 | 3.232 | 2.581 |  |  |  |  |  |  |  |
|  |  | 7 | 9.223 | 4.349 | 3.162 | 2.584 |  |  |  |  |  |  |  |
|  |  | 8 | 9.301 | 4.619 | 3.358 | 2.67 |  |  |  |  |  |  |  |
|  |  | 9 | 8.814 | 4.275 | 3.211 | 2.53 |  |  |  |  |  |  |  |
|  |  | 10 | 9.06 | 4.353 | 3.201 | 2.721 |  |  |  |  |  |  |  |
| 3 | 1 | 1 | 9.079 | 4.252 | 3.232 | 2.732 | 8.9874 | 4.2672 | 3.1239 | 2.5926 | 0.9104 | European | European |
|  |  | 2 | 8.948 | 4.335 | 3.165 | 2.623 |  |  |  |  |  |  |  |
|  |  | 3 | 9.004 | 4.249 | 3.025 | 2.496 |  |  |  |  |  |  |  |
|  |  | 4 | 8.732 | 4.202 | 3.07 | 2.546 |  |  |  |  |  |  |  |
|  |  | 5 | 8.974 | 4.201 | 3.13 | 2.64 |  |  |  |  |  |  |  |
|  |  | 6 | 9.06 | 4.272 | 3.049 | 2.542 |  |  |  |  |  |  |  |
|  |  | 7 | 9.192 | 4.402 | 3.149 | 2.642 |  |  |  |  |  |  |  |
|  |  | 8 | 8.725 | 4.018 | 3.106 | 2.561 |  |  |  |  |  |  |  |
|  |  | 9 | 9.098 | 4.372 | 3.159 | 2.566 |  |  |  |  |  |  |  |
|  |  | 10 | 9.062 | 4.369 | 3.154 | 2.578 |  |  |  |  |  |  |  |
| 4 | 1 | 1 | 8.882 | 4.247 | 3.162 | 2.581 | 8.7865 | 4.1199 | 3.1285 | 2.5692 | -0.7814 | European | European |
|  |  | 2 | 8.697 | 4.043 | 3.102 | 2.577 |  |  |  |  |  |  |  |
|  |  | 3 | 8.796 | 4.17 | 3.13 | 2.574 |  |  |  |  |  |  |  |
|  |  | 4 | 8.895 | 4.144 | 3.213 | 2.561 |  |  |  |  |  |  |  |
|  |  | 5 | 8.682 | 4.052 | 3.122 | 2.618 |  |  |  |  |  |  |  |
|  |  | 6 | 8.69 | 4.074 | 3.129 | 2.563 |  |  |  |  |  |  |  |
|  |  | 7 | 8.901 | 4.209 | 3.197 | 2.54 |  |  |  |  |  |  |  |
|  |  | 8 | 8.744 | 4.114 | 3.124 | 2.567 |  |  |  |  |  |  |  |
|  |  | 9 | 8.773 | 4.102 | 3.084 | 2.615 |  |  |  |  |  |  |  |
|  |  | 10 | 8.805 | 4.044 | 3.022 | 2.496 |  |  |  |  |  |  |  |
| 5 | 1 | 1 | 9.116 | 4.445 | 3.173 | 2.529 | 8.9760 | 4.3148 | 3.1357 | 2.5012 | -1.7761 | African | African |
|  |  | 2 | 9.134 | 4.314 | 3.028 | 2.316 |  |  |  |  |  |  |  |
|  |  | 3 | 9.122 | 4.355 | 3.206 | 2.648 |  |  |  |  |  |  |  |
|  |  | 4 | 8.835 | 4.302 | 3.111 | 2.64 |  |  |  |  |  |  |  |
|  |  | 5 | 8.922 | 4.308 | 3.228 | 2.538 |  |  |  |  |  |  |  |
|  |  | 6 | 8.727 | 4.215 | 3.132 | 2.518 |  |  |  |  |  |  |  |
|  |  | 7 | 8.822 | 4.212 | 3.052 | 2.391 |  |  |  |  |  |  |  |
|  |  | 8 | 8.732 | 4.203 | 3.229 | 2.424 |  |  |  |  |  |  |  |
|  |  | 9 | 9.189 | 4.373 | 3.113 | 2.574 |  |  |  |  |  |  |  |
|  |  | 10 | 9.161 | 4.421 | 3.085 | 2.434 |  |  |  |  |  |  |  |
| 6 | 1 | 1 | 8.897 | 4.264 | 3.18 | 2.39 | 9.0324 | 4.3143 | 3.1947 | 2.5296 | -1.9829 | African | European |
|  |  | 2 | 9.026 | 4.316 | 3.185 | 2.524 |  |  |  |  |  |  |  |
|  |  | 3 | 9.047 | 4.318 | 3.212 | 2.757 |  |  |  |  |  |  |  |
|  |  | 4 | 9.146 | 4.322 | 3.201 | 2.59 |  |  |  |  |  |  |  |
|  |  | 5 | 8.93 | 4.175 | 3.172 | 2.453 |  |  |  |  |  |  |  |
|  |  | 6 | 9.243 | 4.458 | 3.226 | 2.593 |  |  |  |  |  |  |  |
|  |  | 7 | 9.029 | 4.335 | 3.173 | 2.536 |  |  |  |  |  |  |  |
|  |  | 8 | 9.007 | 4.321 | 3.192 | 2.504 |  |  |  |  |  |  |  |
|  |  | 9 | 9.009 | 4.299 | 3.158 | 2.54 |  |  |  |  |  |  |  |
|  |  | 10 | 8.99 | 4.335 | 3.248 | 2.409 |  |  |  |  |  |  |  |
| 7 | 1 | 1 | 8.495 | 4.076 | 3.028 | 2.475 | 8.8663 | 4.2625 | 3.1059 | 2.5770 | 0.4807 | European | African |
|  |  | 2 | 9.108 | 4.371 | 3.167 | 2.587 |  |  |  |  |  |  |  |
|  |  | 3 | 9.035 | 4.336 | 3.134 | 2.696 |  |  |  |  |  |  |  |
|  |  | 4 | 8.839 | 4.233 | 3.107 | 2.626 |  |  |  |  |  |  |  |
|  |  | 5 | 8.873 | 4.313 | 3.088 | 2.479 |  |  |  |  |  |  |  |
|  |  | 6 | 8.99 | 4.341 | 3.168 | 2.556 |  |  |  |  |  |  |  |
|  |  | 7 | 8.858 | 4.282 | 3.173 | 2.626 |  |  |  |  |  |  |  |
|  |  | 8 | 8.87 | 4.196 | 2.973 | 2.519 |  |  |  |  |  |  |  |
|  |  | 9 | 8.823 | 4.197 | 3.137 | 2.546 |  |  |  |  |  |  |  |
|  |  | 10 | 8.772 | 4.28 | 3.084 | 2.66 |  |  |  |  |  |  |  |
| 8 | 1 | 1 | 9.256 | 4.307 | 3.247 | 2.639 | 9.1367 | 4.2741 | 3.1600 | 2.5906 | 0.5782 | European | European |
|  |  | 2 | 9.093 | 4.29 | 3.143 | 2.554 |  |  |  |  |  |  |  |
|  |  | 3 | 9.272 | 4.344 | 3.207 | 2.599 |  |  |  |  |  |  |  |
|  |  | 4 | 9.338 | 4.399 | 3.189 | 2.582 |  |  |  |  |  |  |  |
|  |  | 5 | 8.891 | 4.165 | 2.904 | 2.488 |  |  |  |  |  |  |  |
|  |  | 6 | 9.08 | 4.174 | 3.18 | 2.596 |  |  |  |  |  |  |  |
|  |  | 7 | 8.934 | 4.246 | 3.056 | 2.524 |  |  |  |  |  |  |  |
|  |  | 8 | 9.257 | 4.29 | 3.3 | 2.715 |  |  |  |  |  |  |  |
|  |  | 9 | 9.223 | 4.226 | 3.22 | 2.62 |  |  |  |  |  |  |  |
|  |  | 10 | 9.023 | 4.3 | 3.154 | 2.589 |  |  |  |  |  |  |  |
| 9 | 1 | 1 | 8.509 | 4.081 | 3.003 | 2.44 | 8.7544 | 4.1622 | 3.0886 | 2.4985 | -1.9501 | African | African |
|  |  | 2 | 8.775 | 4.08 | 2.978 | 2.437 |  |  |  |  |  |  |  |
|  |  | 3 | 8.954 | 4.239 | 3.081 | 2.462 |  |  |  |  |  |  |  |
|  |  | 4 | 8.479 | 4.107 | 3.066 | 2.461 |  |  |  |  |  |  |  |
|  |  | 5 | 8.819 | 4.224 | 3.07 | 2.505 |  |  |  |  |  |  |  |
|  |  | 6 | 8.649 | 4.104 | 3.105 | 2.47 |  |  |  |  |  |  |  |
|  |  | 7 | 8.587 | 4.008 | 3.081 | 2.486 |  |  |  |  |  |  |  |
|  |  | 8 | 9.048 | 4.324 | 3.114 | 2.592 |  |  |  |  |  |  |  |
|  |  | 9 | 8.877 | 4.287 | 3.126 | 2.502 |  |  |  |  |  |  |  |
|  |  | 10 | 8.847 | 4.168 | 3.262 | 2.63 |  |  |  |  |  |  |  |
| 10 | 1 | 1 | 8.844 | 4.237 | 3.171 | 2.686 | 8.9255 | 4.2423 | 3.1517 | 2.6252 | 1.0397 | European | European |
|  |  | 2 | 9.132 | 4.362 | 3.294 | 2.679 |  |  |  |  |  |  |  |
|  |  | 3 | 8.807 | 4.24 | 3.072 | 2.669 |  |  |  |  |  |  |  |
|  |  | 4 | 8.671 | 4.029 | 3.058 | 2.55 |  |  |  |  |  |  |  |
|  |  | 5 | 8.942 | 4.193 | 3.128 | 2.546 |  |  |  |  |  |  |  |
|  |  | 6 | 9.121 | 4.288 | 3.089 | 2.608 |  |  |  |  |  |  |  |
|  |  | 7 | 8.913 | 4.257 | 3.227 | 2.647 |  |  |  |  |  |  |  |
|  |  | 8 | 8.77 | 4.23 | 3.059 | 2.609 |  |  |  |  |  |  |  |
|  |  | 9 | 9.027 | 4.337 | 3.297 | 2.677 |  |  |  |  |  |  |  |
|  |  | 10 | 9.028 | 4.25 | 3.122 | 2.581 |  |  |  |  |  |  |  |
| 10 | 2 | 1 | missing | 4.306 | 3.138 | 2.653 | 9.0836 | 4.3471 | 3.1626 | 2.6171 | 1.3180 | European | European |
|  |  | 2 | 9.195 | 4.426 | 3.27 | 2.629 |  |  |  |  |  |  |  |
|  |  | 3 | 9.324 | 4.416 | 3.194 | 2.603 |  |  |  |  |  |  |  |
|  |  | 4 | 8.873 | 4.228 | 3.171 | 2.548 |  |  |  |  |  |  |  |
|  |  | 5 | 9.129 | 4.411 | 3.169 | 2.589 |  |  |  |  |  |  |  |
|  |  | 6 | 9.029 | 4.276 | 3.016 | 2.647 |  |  |  |  |  |  |  |
|  |  | 7 | 9.282 | 4.503 | 3.146 | 2.667 |  |  |  |  |  |  |  |
|  |  | 8 | 8.739 | 4.165 | 3.201 | 2.597 |  |  |  |  |  |  |  |
|  |  | 9 | 9.182 | 4.398 | 3.16 | 2.648 |  |  |  |  |  |  |  |
|  |  | 10 | 8.999 | 4.301 | 3.136 | 2.626 |  |  |  |  |  |  |  |
| 10 | 3 | 1 | 8.774 | 4.074 | 3.181 | 2.603 | 8.9149 | 4.1475 | 3.1478 | 2.5486 | -1.3064 | African | European |
|  |  | 2 | 8.745 | 4.078 | 3.094 | 2.502 |  |  |  |  |  |  |  |
|  |  | 3 | 8.791 | 4.165 | 3.094 | 2.448 |  |  |  |  |  |  |  |
|  |  | 4 | 9.057 | 4.233 | 3.173 | 2.599 |  |  |  |  |  |  |  |
|  |  | 5 | 9.117 | 4.264 | 3.165 | 2.584 |  |  |  |  |  |  |  |
|  |  | 6 | 8.664 | 4.031 | 3.118 | 2.488 |  |  |  |  |  |  |  |
|  |  | 7 | 9.019 | 4.133 | 3.129 | 2.489 |  |  |  |  |  |  |  |
|  |  | 8 | 8.833 | 4.091 | 3.173 | 2.573 |  |  |  |  |  |  |  |
|  |  | 9 | 9.026 | 4.248 | 3.104 | 2.581 |  |  |  |  |  |  |  |
|  |  | 10 | 9.123 | 4.158 | 3.247 | 2.619 |  |  |  |  |  |  |  |
| 10 | 4 | 1 | 9.424 | 4.433 | 3.129 | 2.655 | 9.1392 | 4.3249 | 3.1677 | 2.6241 | 1.5062 | European | European |
|  |  | 2 | 9.277 | 4.37 | 3.23 | 2.639 |  |  |  |  |  |  |  |
|  |  | 3 | 9.241 | 4.293 | 3.217 | 2.703 |  |  |  |  |  |  |  |
|  |  | 4 | 8.743 | 4.126 | 3.213 | 2.634 |  |  |  |  |  |  |  |
|  |  | 5 | 9.126 | 4.335 | 3.21 | 2.637 |  |  |  |  |  |  |  |
|  |  | 6 | 9.011 | 4.22 | 3.09 | 2.529 |  |  |  |  |  |  |  |
|  |  | 7 | 9.378 | 4.448 | 3.242 | 2.746 |  |  |  |  |  |  |  |
|  |  | 8 | 9.135 | 4.321 | 3.148 | 2.595 |  |  |  |  |  |  |  |
|  |  | 9 | 9.048 | 4.355 | 3.114 | 2.721 |  |  |  |  |  |  |  |
|  |  | 10 | 9.009 | 4.348 | 3.084 | 2.382 |  |  |  |  |  |  |  |
| 10 | 5 | 1 | 8.938 | 4.109 | 3.152 | 2.552 | 8.8143 | 4.1223 | 3.0857 | 2.5517 | -0.3502 | European | European |
|  |  | 2 | 8.96 | 4.144 | 3.116 | 2.769 |  |  |  |  |  |  |  |
|  |  | 3 | 8.883 | 4.244 | 3.069 | 2.529 |  |  |  |  |  |  |  |
|  |  | 4 | 8.814 | 4.148 | 2.983 | 2.529 |  |  |  |  |  |  |  |
|  |  | 5 | 8.752 | 4.124 | 3.107 | 2.54 |  |  |  |  |  |  |  |
|  |  | 6 | 8.552 | 4.008 | 3.069 | 2.496 |  |  |  |  |  |  |  |
|  |  | 7 | 8.927 | 4.207 | 3.213 | 2.579 |  |  |  |  |  |  |  |
|  |  | 8 | 8.842 | 4.082 | 3.087 | 2.496 |  |  |  |  |  |  |  |
|  |  | 9 | 8.661 | 4.035 | 2.975 | 2.475 |  |  |  |  |  |  |  |
|  |  | 10 | Frayed | Frayed | 2.987 | 2.501 |  |  |  |  |  |  |  |
| 11 | 1 | 1 | 8.843 | 4.275 | 3.167 | 2.605 | 8.9824 | 4.3406 | 3.1391 | 2.5703 | 0.1737 | European | european |
|  |  | 2 | 8.822 | 4.208 | 3.218 | 2.577 |  |  |  |  |  |  |  |
|  |  | 3 | 8.721 | 4.23 | 3.123 | 2.483 |  |  |  |  |  |  |  |
|  |  | 4 | 9.07 | 4.35 | 3.101 | 2.546 |  |  |  |  |  |  |  |
|  |  | 5 | 8.902 | 4.291 | 3.106 | 2.545 |  |  |  |  |  |  |  |
|  |  | 6 | 9.09 | 4.431 | 3.168 | 2.608 |  |  |  |  |  |  |  |
|  |  | 7 | 9.145 | 4.441 | 3.056 | 2.513 |  |  |  |  |  |  |  |
|  |  | 8 | 9.214 | 4.498 | 3.165 | 2.636 |  |  |  |  |  |  |  |
|  |  | 9 | 9.213 | 4.439 | 3.206 | 2.668 |  |  |  |  |  |  |  |
|  |  | 10 | 8.804 | 4.243 | 3.081 | 2.522 |  |  |  |  |  |  |  |
| 11 | 2 | 1 | 8.826 | 4.085 | 3.068 | 2.517 | 8.9211 | 4.1933 | 3.1170 | 2.5293 | -1.1030 | African | European |
|  |  | 2 | 8.652 | 4.002 | 3.031 | 2.432 |  |  |  |  |  |  |  |
|  |  | 3 | 9.031 | 4.27 | 3.107 | 2.552 |  |  |  |  |  |  |  |
|  |  | 4 | 9.109 | 4.336 | 3.163 | 2.588 |  |  |  |  |  |  |  |
|  |  | 5 | 8.788 | 4.145 | 3.103 | 2.554 |  |  |  |  |  |  |  |
|  |  | 6 | 8.816 | 4.083 | 3.089 | 2.463 |  |  |  |  |  |  |  |
|  |  | 7 | 8.946 | 4.209 | 3.129 | 2.521 |  |  |  |  |  |  |  |
|  |  | 8 | 9.289 | 4.291 | 3.208 | 2.557 |  |  |  |  |  |  |  |
|  |  | 9 | 9.005 | 4.262 | 3.231 | 2.618 |  |  |  |  |  |  |  |
|  |  | 10 | 8.749 | 4.25 | 3.041 | 2.491 |  |  |  |  |  |  |  |
| 11 | 3 | 1 | 9.182 | 4.356 | 3.005 | 2.478 | 9.1216 | 4.3929 | 3.1094 | 2.5656 | 1.1469 | European | European |
|  |  | 2 | 9.189 | 4.351 | 3.114 | 2.571 |  |  |  |  |  |  |  |
|  |  | 3 | 8.83 | 4.348 | 3.166 | 2.56 |  |  |  |  |  |  |  |
|  |  | 4 | 9.004 | 4.39 | 3.161 | 2.566 |  |  |  |  |  |  |  |
|  |  | 5 | 9.056 | 4.426 | 3.042 | 2.542 |  |  |  |  |  |  |  |
|  |  | 6 | 9.401 | 4.494 | 3.171 | 2.595 |  |  |  |  |  |  |  |
|  |  | 7 | 9.31 | 4.471 | 3.187 | 2.693 |  |  |  |  |  |  |  |
|  |  | 8 | 9.222 | 4.415 | 3.224 | 2.633 |  |  |  |  |  |  |  |
|  |  | 9 | 9.282 | 4.45 | 3.03 | 2.5 |  |  |  |  |  |  |  |
|  |  | 10 | 8.74 | 4.228 | 2.994 | 2.518 |  |  |  |  |  |  |  |
| 11 | 4 | 1 | 9.007 | 4.316 | 3.187 | 2.635 | 8.9761 | 4.2799 | 3.1923 | 2.5941 | -0.3809 | European | European |
|  |  | 2 | 8.931 | 4.347 | 3.224 | 2.565 |  |  |  |  |  |  |  |
|  |  | 3 | 9.137 | 4.354 | 3.359 | 2.644 |  |  |  |  |  |  |  |
|  |  | 4 | 8.835 | 4.226 | 3.279 | 2.664 |  |  |  |  |  |  |  |
|  |  | 5 | 9.029 | 4.33 | 3.256 | 2.567 |  |  |  |  |  |  |  |
|  |  | 6 | 8.726 | 4.162 | 2.956 | 2.434 |  |  |  |  |  |  |  |
|  |  | 7 | 9.168 | 4.224 | 3.085 | 2.65 |  |  |  |  |  |  |  |
| 11 | 5 | 1 | 9.296 | 4.443 | 3.154 | 2.489 | 9.1067 | 4.3735 | 3.0850 | 2.5238 | 0.3646 | European | European |
|  |  | 2 | 9.093 | 4.419 | 3.1 | 2.648 |  |  |  |  |  |  |  |
|  |  | 3 | 9.053 | 4.329 | 3.08 | 2.473 |  |  |  |  |  |  |  |
|  |  | 4 | 9.121 | 4.336 | 2.916 | 2.368 |  |  |  |  |  |  |  |
|  |  | 5 | 8.946 | 4.375 | 3.006 | 2.534 |  |  |  |  |  |  |  |
|  |  | 6 | 9.131 | 4.339 | 3.254 | 2.631 |  |  |  |  |  |  |  |
| 11 | 6 | 1 | 9.491 | 4.505 | 3.033 | 2.672 | 9.1298 | 4.3148 | 3.1478 | 2.6203 | 1.7363 | European | European |
|  |  | 2 | 9.037 | 4.277 | 3.243 | 2.697 |  |  |  |  |  |  |  |
|  |  | 3 | 9.145 | 4.361 | 3.258 | 2.682 |  |  |  |  |  |  |  |
|  |  | 4 | 8.863 | 4.192 | 2.916 | 2.483 |  |  |  |  |  |  |  |
|  |  | 5 | 9.048 | 4.253 | 3.068 | 2.507 |  |  |  |  |  |  |  |
|  |  | 6 | 9.265 | 4.39 | 3.246 | 2.693 |  |  |  |  |  |  |  |
|  |  | 7 | 9.091 | 4.335 | 3.245 | 2.656 |  |  |  |  |  |  |  |
|  |  | 8 | 9.225 | 4.248 | 3.279 | 2.666 |  |  |  |  |  |  |  |
|  |  | 9 | 9.307 | 4.42 | 3.208 | 2.652 |  |  |  |  |  |  |  |
|  |  | 10 | 8.826 | 4.167 | 2.982 | 2.495 |  |  |  |  |  |  |  |
| 11 | 7 | 1 | 8.914 | 4.324 | 3.096 | 2.573 | 9.1597 | 4.3723 | 3.1135 | 2.6365 | 3.0960 | European | European |
|  |  | 2 | 9.115 | 4.341 | 3.087 | 2.533 |  |  |  |  |  |  |  |
|  |  | 3 | 9.475 | 4.501 | 3.038 | 2.608 |  |  |  |  |  |  |  |
|  |  | 4 | 9.116 | 4.302 | 3.144 | 2.819 |  |  |  |  |  |  |  |
|  |  | 5 | 9.086 | 4.326 | 3.095 | 2.552 |  |  |  |  |  |  |  |
|  |  | 6 | 9.252 | 4.44 | 3.221 | 2.734 |  |  |  |  |  |  |  |
| 11 | 8 | 1 | 8.9 | 4.211 | 3.157 | 2.692 | 8.8630 | 4.2351 | 3.1305 | 2.6162 | 1.0116 | European | European |
|  |  | 2 | 9.28 | 4.484 | 3.101 | 2.604 |  |  |  |  |  |  |  |
|  |  | 3 | 8.736 | 4.138 | 3.111 | 2.668 |  |  |  |  |  |  |  |
|  |  | 4 | 8.648 | 3.981 | 3.096 | 2.415 |  |  |  |  |  |  |  |
|  |  | 5 | 8.811 | 4.252 | 3.239 | 2.772 |  |  |  |  |  |  |  |
|  |  | 6 | 9.029 | 4.348 | 3.138 | 2.72 |  |  |  |  |  |  |  |
|  |  | 7 | 9.051 | 4.246 | 3.059 | 2.569 |  |  |  |  |  |  |  |
|  |  | 8 | 8.563 | 4.158 | 3.068 | 2.536 |  |  |  |  |  |  |  |
|  |  | 9 | 8.658 | 4.157 | 3.037 | 2.571 |  |  |  |  |  |  |  |
|  |  | 10 | 8.954 | 4.376 | 3.299 | 2.615 |  |  |  |  |  |  |  |
| 11 | 14 | 1 | 8.747 | 4.269 | 3.096 | 2.524 | 8.7732 | 4.2370 | 3.0589 | 2.5087 | -0.8320 | European | European |
|  |  | 2 | 8.63 | 4.247 | 3.12 | 2.513 |  |  |  |  |  |  |  |
|  |  | 3 | 8.757 | 4.282 | 3.025 | 2.512 |  |  |  |  |  |  |  |
|  |  | 4 | 8.952 | 4.247 | 3.031 | 2.528 |  |  |  |  |  |  |  |
|  |  | 5 | 8.862 | 4.344 | 3.04 | 2.438 |  |  |  |  |  |  |  |
|  |  | 6 | 8.642 | 4.168 | 3.067 | 2.614 |  |  |  |  |  |  |  |
|  |  | 7 | 8.648 | 4.168 | 3.006 | 2.539 |  |  |  |  |  |  |  |
|  |  | 8 | 8.942 | 4.246 | 3.083 | 2.531 |  |  |  |  |  |  |  |
|  |  | 9 | 8.724 | 4.188 | 3.003 | 2.3 |  |  |  |  |  |  |  |
|  |  | 10 | 8.828 | 4.211 | 3.118 | 2.588 |  |  |  |  |  |  |  |

**Table 3b**. Morphometric measurements, discriminant function scores and outcomes, and cytochrome b restriction results for honey bees collected from 10 feral hives in San Diego County: FWL, fore wing length; HWL, hind wing lenghth; TL, tibia length; FL, femur length (all in mm).

| Hive | Bee | Latitude | Longitude | FWL | HWL | TL | FL | Ave. FL | Ave. HWL | Ave.TL | Ave. FL | Disc. score | Disc. result | cytB result |
| --- | --- | --- | --- | --- | --- | --- | --- | --- | --- | --- | --- | --- | --- | --- |
| 1 | F1 | 32.749049 | -117.63217 | 8.492 | 4.165 | 3 | 2.48 | 8.6446 | 4.2114 | 2.9791 | 2.4517 | -1.28699 | African | African |
|  | F2 |  |  | 8.712 | 4.189 | 3.042 | 2.468 |  |  |  |  |  |  |  |
|  | F3 |  |  | 8.728 | 4.189 | 3.009 | 2.434 |  |  |  |  |  |  |  |
|  | F4 |  |  | 8.46 | 4.247 | 2.998 | 2.432 |  |  |  |  |  |  |  |
|  | F5 |  |  | 8.712 | 4.227 | 2.944 | 2.484 |  |  |  |  |  |  |  |
|  | F6 |  |  | 8.539 | 4.196 | 2.99 | 2.403 |  |  |  |  |  |  |  |
|  | F7 |  |  | frayed | frayed | 2.974 | 2.502 |  |  |  |  |  |  |  |
|  | F8 |  |  | 8.695 | 4.255 | 2.95 | 2.44 |  |  |  |  |  |  |  |
|  | F9 |  |  | 8.723 | 4.207 | 2.946 | 2.483 |  |  |  |  |  |  |  |
|  | F10 |  |  | 8.74 | 4.228 | 2.933 | 2.441 |  |  |  |  |  |  |  |
| 2 | F11 | 32.82299 | -117.05395 | 8.741 | 4.235 | 2.981 | 2.473 | 8.6844 | 4.1910 | 2.9077 | 2.4560 | 0.28531 | European | African |
|  | F12 |  |  | 8.906 | 4.286 | 2.912 | 2.559 |  |  |  |  |  |  |  |
|  | F13 |  |  | 8.543 | 4.096 | 2.863 | 2.467 |  |  |  |  |  |  |  |
|  | F14 |  |  | 8.562 | 4.262 | 2.83 | 2.427 |  |  |  |  |  |  |  |
|  | F15 |  |  | 8.256 | 3.998 | 2.717 | 2.339 |  |  |  |  |  |  |  |
|  | F16 |  |  | 8.891 | 4.37 | 2.952 | 2.547 |  |  |  |  |  |  |  |
|  | F17 |  |  | 8.679 | 4.167 | 3.006 | 2.521 |  |  |  |  |  |  |  |
|  | F18 |  |  | 8.88 | 4.201 | 3.013 | 2.354 |  |  |  |  |  |  |  |
|  | F19 |  |  | 8.786 | 4.093 | 2.937 | 2.494 |  |  |  |  |  |  |  |
|  | F20 |  |  | 8.6 | 4.202 | 2.866 | 2.379 |  |  |  |  |  |  |  |
| 3 | F21 | 32.822365 | -117.01544 | 8.657 | 3.954 | 2.818 | 2.386 | 8.5714 | 4.0728 | 2.9179 | 2.4355 | -1.11458 | African | African |
|  | F22 |  |  | 8.553 | 4.123 | 2.926 | 2.499 |  |  |  |  |  |  |  |
|  | F23 |  |  | frayed | frayed | 2.844 | 2.401 |  |  |  |  |  |  |  |
|  | F24 |  |  | 8.471 | 4.084 | 2.926 | 2.448 |  |  |  |  |  |  |  |
|  | F25 |  |  | frayed | frayed | 2.892 | 2.336 |  |  |  |  |  |  |  |
|  | F26 |  |  | 8.589 | 4.015 | 2.943 | 2.473 |  |  |  |  |  |  |  |
|  | F27 |  |  | 8.243 | 3.886 | 2.832 | 2.343 |  |  |  |  |  |  |  |
|  | F28 |  |  | 8.732 | 4.116 | 3.075 | 2.423 |  |  |  |  |  |  |  |
|  | F29 |  |  | 8.564 | 4.046 | 2.887 | 2.496 |  |  |  |  |  |  |  |
|  | F30 |  |  | 8.762 | 4.358 | 2.936 | 2.416 |  |  |  |  |  |  |  |
| 4 | F31 | 32.822123 | -117.06949 | 8.812 | 4.134 | 3.069 | 2.553 | 8.7431 | 4.1787 | 3.0482 | 2.5163 | -0.64988 | European | African |
|  | F32 |  |  | 8.69 | 4.265 | 3.041 | 2.504 |  |  |  |  |  |  |  |
|  | F33 |  |  | 8.604 | 4.004 | 2.964 | 2.44 |  |  |  |  |  |  |  |
|  | F34 |  |  | 8.816 | 4.235 | 3.132 | 2.568 |  |  |  |  |  |  |  |
|  | F35 |  |  | 8.633 | 4.079 | 2.958 | 2.499 |  |  |  |  |  |  |  |
|  | F36 |  |  | 8.72 | 4.18 | 2.969 | 2.507 |  |  |  |  |  |  |  |
|  | F37 |  |  | 8.772 | 4.224 | 3.023 | 2.495 |  |  |  |  |  |  |  |
|  | F38 |  |  | 8.745 | 4.251 | 3.112 | 2.539 |  |  |  |  |  |  |  |
|  | F39 |  |  | 8.865 | 4.227 | 3.157 | 2.547 |  |  |  |  |  |  |  |
|  | F40 |  |  | 8.774 | 4.188 | 3.057 | 2.511 |  |  |  |  |  |  |  |
| 5 | F41 | 32.845184 | -117.04648 | 8.563 | 4.032 | 2.808 | 2.402 | 8.6883 | 4.1577 | 2.9046 | 2.4853 | 1.08542 | European | African |
|  | F42 |  |  | 9.064 | 4.323 | 3.045 | 2.542 |  |  |  |  |  |  |  |
|  | F43 |  |  | 8.502 | 4.069 | 2.75 | 2.388 |  |  |  |  |  |  |  |
|  | F44 |  |  | 8.611 | 4.161 | 2.836 | 2.396 |  |  |  |  |  |  |  |
|  | F45 |  |  | 8.636 | 4.157 | 2.914 | 2.551 |  |  |  |  |  |  |  |
|  | F46 |  |  | 8.771 | 4.174 | 2.976 | 2.533 |  |  |  |  |  |  |  |
|  | F47 |  |  | 8.588 | 4.126 | 2.987 | 2.576 |  |  |  |  |  |  |  |
|  | F48 |  |  | 8.72 | 4.223 | 2.86 | 2.408 |  |  |  |  |  |  |  |
|  | F49 |  |  | 8.546 | 4.083 | 2.793 | 2.393 |  |  |  |  |  |  |  |
|  | F50 |  |  | 8.882 | 4.229 | 3.077 | 2.664 |  |  |  |  |  |  |  |
| 6 | F51 | 32.821506 | -117.046799 | 8.432 | 3.891 | 3.052 | 2.466 | 8.6155 | 4.0505 | 2.9962 | 2.5016 | -0.73883 | European | African |
|  | F52 |  |  | 8.594 | 4.004 | 2.953 | 2.48 |  |  |  |  |  |  |  |
|  | F53 |  |  | 8.579 | 4.095 | 3.009 | 2.466 |  |  |  |  |  |  |  |
|  | F54 |  |  | 8.611 | 4.069 | 3.01 | 2.499 |  |  |  |  |  |  |  |
|  | F55 |  |  | 8.534 | 3.984 | 3.014 | 2.529 |  |  |  |  |  |  |  |
|  | F56 |  |  | 8.38 | 3.736 | 2.853 | 2.48 |  |  |  |  |  |  |  |
|  | F57 |  |  | 8.852 | 4.202 | 3.03 | 2.521 |  |  |  |  |  |  |  |
|  | F58 |  |  | 8.436 | 4.096 | 2.976 | 2.452 |  |  |  |  |  |  |  |
|  | F59 |  |  | 8.956 | 4.222 | 3.104 | 2.561 |  |  |  |  |  |  |  |
|  | F60 |  |  | 8.781 | 4.206 | 2.961 | 2.562 |  |  |  |  |  |  |  |
| 7 | F61 | 32.7921 | -117.0607 | 8.633 | 4.023 | 3.011 | 2.569 | 8.6960 | 4.0278 | 2.9768 | 2.5303 | 0.60301 | European | European |
|  | F62 |  |  | 8.537 | frayed | 3.056 | 2.508 |  |  |  |  |  |  |  |
|  | F63 |  |  | 8.72 | 3.94 | 2.97 | 2.512 |  |  |  |  |  |  |  |
|  | F64 |  |  | 8.733 | 3.949 | 3.047 | 2.547 |  |  |  |  |  |  |  |
|  | F65 |  |  | frayed | frayed | 3.055 | 2.495 |  |  |  |  |  |  |  |
|  | F66 |  |  | 8.52 | 4.113 | 2.902 | 2.439 |  |  |  |  |  |  |  |
|  | F67 |  |  | frayed | 4.003 | 3.099 | 2.488 |  |  |  |  |  |  |  |
|  | F68 |  |  | 8.699 | 4.025 | 2.98 | 2.451 |  |  |  |  |  |  |  |
|  | F69 |  |  | 8.871 | 4.117 | 2.951 | 2.664 |  |  |  |  |  |  |  |
|  | F70 |  |  | frayed | 4.16 | 2.981 | 2.478 |  |  |  |  |  |  |  |
| 8 | F71 | 32.8556 | -117.1898 | 8.698 | 4.173 | 2.909 | 2.484 | 8.6358 | 4.1405 | 2.9250 | 2.4657 | -0.05149 | European | African |
|  | F72 |  |  | 8.437 | 4.03 | 2.79 | 2.418 |  |  |  |  |  |  |  |
|  | F73 |  |  | 8.753 | 4.204 | 2.904 | 2.487 |  |  |  |  |  |  |  |
|  | F74 |  |  | 8.715 | 4.295 | 2.994 | 2.474 |  |  |  |  |  |  |  |
|  | F75 |  |  | 8.814 | 4.134 | 3.007 | 2.545 |  |  |  |  |  |  |  |
|  | F76 |  |  | 8.429 | 4.1 | 2.905 | 2.467 |  |  |  |  |  |  |  |
|  | F77 |  |  | 8.72 | 4.197 | 2.998 | 2.487 |  |  |  |  |  |  |  |
|  | F78 |  |  | 8.533 | 4.016 | 2.859 | 2.342 |  |  |  |  |  |  |  |
|  | F79 |  |  | 8.547 | 4.123 | 2.909 | 2.391 |  |  |  |  |  |  |  |
|  | F80 |  |  | 8.712 | 4.133 | 2.975 | 2.562 |  |  |  |  |  |  |  |
| 9 | F81 | 32.748935 | -117.03230 | 8.677 | 4.11 | 3.031 | 2.507 | 8.6890 | 4.0963 | 3.0254 | 2.4747 | -1.73584 | African | European |
|  | F82 |  |  | 8.86 | frayed | 3 | 2.528 |  |  |  |  |  |  |  |
|  | F83 |  |  | 8.523 | 4.037 | 3.009 | 2.502 |  |  |  |  |  |  |  |
|  | F84 |  |  | frayed | 3.936 | 3.124 | 2.549 |  |  |  |  |  |  |  |
|  | F85 |  |  | 8.662 | 4.111 | 2.964 | 2.476 |  |  |  |  |  |  |  |
|  | F86 |  |  | frayed | frayed | 3.104 | 2.582 |  |  |  |  |  |  |  |
|  | F87 |  |  | 8.684 | 4.068 | 2.98 | 2.42 |  |  |  |  |  |  |  |
|  | F88 |  |  | 8.914 | 4.242 | 3.219 | 2.426 |  |  |  |  |  |  |  |
|  | F89 |  |  | 8.565 | 4.004 | 2.897 | 2.476 |  |  |  |  |  |  |  |
|  | F90 |  |  | 8.798 | 4.102 | 3.078 | 2.516 |  |  |  |  |  |  |  |
| 10 | F91 | 33.08214 | -117.15437 | 8.594 | 4.121 | 2.955 | 2.414 | 8.7458 | 4.0431 | 2.9078 | 2.4567 | 0.07509 | European | European |
|  | F92 |  |  | 8.76 | 4.129 | 3.001 | 2.521 |  |  |  |  |  |  |  |
|  | F93 |  |  | 8.752 | 4.138 | 2.83 | 2.475 |  |  |  |  |  |  |  |
|  | F94 |  |  | 8.708 | 4.212 | 2.768 | 2.48 |  |  |  |  |  |  |  |
|  | F95 |  |  | 8.71 | 3.998 | 3.054 | 2.539 |  |  |  |  |  |  |  |
|  | F96 |  |  | 8.874 | 4.284 | 2.88 | 2.558 |  |  |  |  |  |  |  |
|  | F97 |  |  | 8.859 | 3.16 | 2.887 | 2.587 |  |  |  |  |  |  |  |
|  | F98 |  |  | 8.667 | 4.074 | 2.847 | 2.442 |  |  |  |  |  |  |  |
|  | F99 |  |  | 8.745 | 4.242 | 2.908 | 2.22 |  |  |  |  |  |  |  |
|  | F100 |  |  | 8.789 | 4.073 | 2.948 | 2.331 |  |  |  |  |  |  |  |
