# Supplementary material for: Range and Frequency of Africanized Honey Bees in California (USA)
Source: PLoS One. 2015 Sep 11;10(9):e0137407. doi: 10.1371/journal.pone.0137407 (PMC4567290; doi:10.1371/journal.pone.0137407)
Supplement: S4 Table — (DOCX) [file pone.0137407.s004.docx]

**Table S4.** Genbank accession numbers for honey bee mitochondrial COI – COII spacer region sequences used in Figure S1.

| Sequence | Accession |
| --- | --- |
| A1a | FJ477984.1 |
| A1b | FJ477985.1 |
| A1c | FJ477986.1 |
| A4a | FJ478009.1 |
| A8 | FJ477981.1 |
| A9 | FJ477982.1 |
| A14 | FJ477991.1 |
| A25 | FJ477989.1 |
| A26 | FJ477990.1 |
| A27 | FJ477983.1 |
| C1 | FJ478010.1 |
| C11 | FJ037776.1 |
| C12 | FJ037777.1 |
| C14 | FJ037778.1 |
| M3 | FJ478004.1 |
| M4 | FJ478006.1 |
| M6 | FJ478008.1 |
| M7 | FJ478005.1 |
| M8 | FJ478007.1 |
| O1a | FJ477992.1 |
| O1b | FJ477993.1 |
| O1c | FJ477994.1 |
| O1d | FJ477995.1 |
| O2 | FJ477996.1 |
| O3 | FJ477997.1 |
| Y1a | FJ477998.1 |
| Y1b | FJ477999.1 |
| Y2a | FJ478000.1 |
| Y2b | FJ478001.1 |
| Y2c | FJ478002.1 |
| Y2d | FJ478003.1 |
