# Supplementary material for: Range and Frequency of Africanized Honey Bees in California (USA)
Source: PLoS One. 2015 Sep 11;10(9):e0137407. doi: 10.1371/journal.pone.0137407 (PMC4567290; doi:10.1371/journal.pone.0137407)
Supplement: S5 Table — Honey bee sequences scored as African have a cytosine at position 2382 (Crozier and Crozier 1993) within the COI gene, all others have a thymine at that position. (DOCX) [file pone.0137407.s005.docx]

**Table S5.** Collection information from 402 high quality sequences of honey bees (*Apis mellifera*) from the Barcode of Life Database (barcode index number (BIN) AAA2326). Honey bee sequences scored as African have a cytosine at position 2382 (Crozier and Crozier 1993) within the COI gene, all others have a thymine at that position.

| Country | Province | Collection Date | African | Total |
| --- | --- | --- | --- | --- |
| Argentina | Corrientes | 1/1/10 | 6 | 6 |
| Argentina | Misiones | 1/6/08 | 2 | 2 |
| Argentina | Misiones | 1/20/08 | 1 | 1 |
| Argentina | Misiones | 4/11/11 | 3 | 3 |
| Australia | Australian Capital Territory | 10/16/10 | 0 | 2 |
| Australia | Australian Capital Territory | 10/29/10 | 0 | 1 |
| Australia | New South Wales | 12/13/10 | 0 | 1 |
| Australia | Queensland | 7/7/08 | 0 | 1 |
| Australia | South Australia | 12/5/11 | 0 | 3 |
| Australia | South Australia | 12/7/11 | 0 | 1 |
| Australia | South Australia | 12/9/11 | 0 | 1 |
| Australia | South Australia | 12/10/11 | 0 | 2 |
| Australia | Tasmania | 3/14/10 | 0 | 1 |
| Bolivia | Santa Cruz | 1/5/92 | 1 | 1 |
| Canada | Alberta | 8/8/10 | 0 | 1 |
| Canada | Alberta | 6/10/12 | 0 | 5 |
| Canada | British Columbia | 7/3/10 | 0 | 1 |
| Canada | British Columbia | 6/13/13 | 0 | 1 |
| Canada | British Columbia | 7/11/13 | 0 | 1 |
| Canada | British Columbia | 8/1/13 | 0 | 3 |
| Canada | Nova Scotia | 6/4/03 | 0 | 3 |
| Canada | Nova Scotia | 6/27/03 | 0 | 1 |
| Canada | Nova Scotia | 8/3/13 | 0 | 1 |
| Canada | Ontario | 10/1/00 | 0 | 1 |
| Canada | Ontario | 6/28/07 | 0 | 1 |
| Canada | Ontario | 7/1/07 | 0 | 3 |
| Canada | Ontario | 7/2/07 | 0 | 5 |
| Canada | Ontario | 7/5/07 | 0 | 4 |
| Canada | Ontario | 7/6/07 | 0 | 3 |
| Canada | Ontario | 7/9/07 | 0 | 2 |
| Canada | Ontario | 7/12/07 | 0 | 3 |
| Canada | Ontario | 7/16/07 | 0 | 6 |
| Canada | Ontario | 7/19/07 | 0 | 5 |
| Canada | Ontario | 7/23/07 | 0 | 3 |
| Canada | Ontario | 7/26/07 | 0 | 5 |
| Canada | Ontario | 7/30/07 | 0 | 2 |
| Canada | Ontario | 8/6/07 | 0 | 1 |
| Canada | Ontario | 8/10/07 | 0 | 2 |
| Canada | Ontario | 8/13/07 | 0 | 1 |
| Canada | Ontario | 8/16/07 | 0 | 1 |
| Canada | Ontario | 8/17/07 | 0 | 2 |
| Canada | Ontario | 8/23/07 | 0 | 1 |
| Canada | Ontario | 9/3/07 | 0 | 1 |
| Canada | Ontario | 9/4/07 | 0 | 1 |
| Canada | Ontario | 5/31/08 | 0 | 1 |
| Canada | Ontario | 6/9/08 | 0 | 6 |
| Canada | Ontario | 6/20/08 | 0 | 1 |
| Canada | Ontario | 7/4/08 | 0 | 1 |
| Canada | Ontario | 7/24/08 | 0 | 1 |
| Canada | Ontario | 8/7/08 | 0 | 2 |
| Canada | Ontario | 8/27/08 | 0 | 3 |
| Canada | Ontario | 8/31/09 | 0 | 8 |
| Canada | Ontario | 4/3/10 | 0 | 1 |
| Canada | Ontario | 5/1/10 | 0 | 1 |
| Canada | Ontario | 5/15/10 | 0 | 1 |
| Canada | Ontario | 6/29/10 | 0 | 1 |
| Canada | Ontario | 7/5/10 | 0 | 1 |
| Canada | Ontario | 8/18/10 | 0 | 1 |
| Canada | Ontario | 9/15/10 | 0 | 1 |
| Canada | Ontario | 9/23/10 | 0 | 1 |
| Canada | Ontario | 9/28/10 | 0 | 1 |
| Canada | Ontario | 10/15/10 | 0 | 3 |
| Canada | Ontario | 10/27/10 | 0 | 1 |
| Canada | Ontario | 6/17/11 | 0 | 2 |
| Canada | Ontario | 9/21/11 | 0 | 1 |
| Canada | Ontario | 5/21/12 | 0 | 1 |
| Canada | Ontario | 6/1/12 | 0 | 1 |
| Canada | Ontario | 6/3/12 | 0 | 1 |
| Canada | Ontario | 6/29/12 | 0 | 1 |
| Canada | Ontario | 9/15/13 | 0 | 2 |
| Canada | Ontario | 9/27/13 | 0 | 4 |
| Canada | Ontario | 10/17/13 | 0 | 1 |
| Canada | Prince Edward Island | 7/10/13 | 0 | 1 |
| Colombia | Huila | 11/27/01 | 1 | 1 |
| Colombia | Risaralda | 12/17/02 | 1 | 1 |
| Costa Rica | Alajuela | 4/26/94 | 1 | 1 |
| Costa Rica | Cartago | 10/19/10 | 1 | 1 |
| Costa Rica | Guanacaste | 4/9/12 | 1 | 1 |
| Costa Rica | Guanacaste | 11/26/12 | 1 | 1 |
| Costa Rica | Limon | 5/29/10 | 1 | 1 |
| Costa Rica | Puntarenas | 4/4/06 | 1 | 1 |
| Egypt | Alexandria | 6/9/13 | 0 | 1 |
| Egypt | Alexandria | 7/9/13 | 0 | 1 |
| Egypt | Alexandria | 8/4/13 | 0 | 1 |
| Egypt | Alexandria | 8/20/13 | 0 | 1 |
| Egypt |  | 9/14/06 | 0 | 3 |
| Finland |  | 6/18/00 | 0 | 1 |
| Finland |  | 7/29/03 | 0 | 1 |
| Finland |  | 5/24/05 | 0 | 1 |
| Germany | Bavaria | 7/25/12 | 0 | 9 |
| Honduras | Cortes | 7/6/12 | 1 | 1 |
| Honduras | Cortes | 6/30/13 | 1 | 1 |
| Kenya | Coast | 6/24/11 | 10 | 10 |
| Kenya | Coast | 6/27/11 | 8 | 8 |
| Kenya | Coast | 1/24/12 | 1 | 1 |
| Kenya | Coast | 5/30/13 | 1 | 1 |
| Kenya | Eastern | 4/8/09 | 1 | 1 |
| Kenya | Nairobi | 5/10/06 | 3 | 3 |
| Kenya | Nairobi | 12/2/09 | 1 | 1 |
| Kenya | Nyanza | 10/17/10 | 9 | 9 |
| Kenya | Rift Valley | 3/31/08 | 1 | 1 |
| Kenya | Rift Valley | 9/9/11 | 2 | 2 |
| Kenya | Western | 10/20/10 | 2 | 2 |
| Kenya | Western | 10/21/10 | 1 | 1 |
| Kenya |  |  | 0 | 1 |
| Kenya |  | 10/14/12 | 1 | 1 |
| Madagascar |  | 7/15/06 | 1 | 4 |
| Mexico | Chiapas | 12/23/08 | 1 | 1 |
| Mexico | Chiapas | 12/30/08 | 1 | 1 |
| Mexico | Chiapas | 1/2/09 | 1 | 1 |
| Mexico | Chiapas | 1/11/09 | 2 | 2 |
| Mexico | Chiapas | 1/12/09 | 1 | 1 |
| Mexico | Chiapas | 1/15/09 | 1 | 1 |
| Mexico | Chiapas | 1/17/09 | 2 | 2 |
| Mexico | Jalisco | 6/27/13 | 1 | 1 |
| Pakistan | Khyber Pakhtunkhwa | 8/30/12 | 0 | 1 |
| Pakistan | Khyber Pakhtunkhwa | 8/31/12 | 0 | 2 |
| Pakistan | Punjab | 4/29/10 | 0 | 1 |
| Panama | Panama | 3/24/09 | 1 | 1 |
| Panama | Panama | 11/15/09 | 1 | 1 |
| Panama | Panama | 11/17/09 | 1 | 1 |
| Panama | Panama | 5/14/10 | 2 | 2 |
| Russia | Primorsky Krai | 7/21/13 | 0 | 23 |
| South Africa | Gauteng | 2/16/12 | 1 | 1 |
| South Africa | Gauteng | 4/8/12 | 1 | 1 |
| South Africa | Gauteng | 11/1/12 | 1 | 1 |
| South Africa | KwaZulu-Natal | 11/30/11 | 2 | 2 |
| South Africa | KwaZulu-Natal | 4/20/12 | 3 | 3 |
| South Africa | KwaZulu-Natal | 2/13/13 | 9 | 9 |
| South Africa | KwaZulu-Natal | 2/20/13 | 7 | 7 |
| South Africa | KwaZulu-Natal | 3/14/13 | 3 | 3 |
| South Africa | KwaZulu-Natal | 3/18/13 | 3 | 3 |
| South Africa | KwaZulu-Natal | 4/9/13 | 1 | 1 |
| South Africa | KwaZulu-Natal | 4/16/13 | 1 | 1 |
| South Africa | KwaZulu-Natal | 4/17/13 | 4 | 4 |
| South Africa | KwaZulu-Natal |  | 3 | 3 |
| South Africa | Western Cape | 9/24/10 | 2 | 2 |
| South Korea |  |  | 0 | 5 |
| Tajikistan | Gorno-Badakhshan | 6/1/09 | 0 | 1 |
| United States | Alaska | 5/25/10 | 0 | 2 |
| United States | Arizona | 7/21/05 | 1 | 1 |
| United States | Arizona | 5/18/07 | 0 | 1 |
| United States | Arizona | 7/12/07 | 4 | 4 |
| United States | Arizona | 9/20/07 | 0 | 1 |
| United States | Arizona | 9/20/07 | 2 | 2 |
| United States | Arizona | 4/22/09 | 1 | 1 |
| United States | Arizona | 6/2/09 | 1 | 1 |
| United States | Arizona | 3/30/10 | 1 | 2 |
| United States | Arizona | 4/15/10 | 0 | 1 |
| United States | Arizona | 4/22/10 | 4 | 4 |
| United States | Arizona | 4/26/10 | 1 | 1 |
| United States | Arizona | 9/5/10 | 1 | 1 |
| United States | Arizona | 6/2/11 | 1 | 1 |
| United States | Arizona | 6/3/11 | 1 | 1 |
| United States | Arizona | 6/4/11 | 1 | 1 |
| United States | Arizona | 6/5/11 | 2 | 2 |
| United States | Arizona | 6/8/11 | 1 | 1 |
| United States | Arizona | 7/13/11 | 2 | 2 |
| United States | Arizona | 7/15/11 | 1 | 1 |
| United States | Arizona | 7/16/11 | 1 | 1 |
| United States | Arkansas | 5/12/09 | 0 | 1 |
| United States | Arkansas | 7/2/11 | 0 | 1 |
| United States | California | 7/20/11 | 3 | 3 |
| United States | California | 7/22/11 | 1 | 1 |
| United States | California | 7/25/11 | 1 | 1 |
| United States | California | 7/25/11 | 1 | 1 |
| United States | California | 7/29/11 | 1 | 1 |
| United States | California | 7/31/11 | 0 | 1 |
| United States | California | 8/1/11 | 0 | 1 |
| United States | California | 8/9/11 | 2 | 2 |
| United States | California | 6/14/13 | 1 | 1 |
| United States | California | 8/23/13 | 2 | 2 |
| United States | California | 9/14/13 | 5 | 7 |
| United States | California | 11/14/13 | 0 | 1 |
| United States | California | 5/2/14 | 1 | 1 |
| United States | California | 5/23/14 | 1 | 1 |
| United States | California | 6/6/14 | 0 | 1 |
| United States | Colorado | 6/13/09 | 0 | 1 |
| United States | Colorado | 5/9/10 | 0 | 3 |
| United States | Colorado | 5/10/10 | 0 | 2 |
| United States | Florida | 6/20/06 | 1 | 1 |
| United States | Florida | 3/10/10 | 0 | 1 |
| United States | Florida | 5/7/11 | 1 | 1 |
| United States | Florida | 5/8/11 | 3 | 3 |
| United States | Florida | 5/10/11 | 1 | 1 |
| United States | Florida | 5/11/11 | 1 | 2 |
| United States | Florida | 5/12/11 | 1 | 1 |
| United States | Florida | 5/18/11 | 0 | 2 |
| United States | Massachusetts | 9/28/10 | 0 | 1 |
| United States | Massachusetts | 9/29/10 | 0 | 2 |
| United States | New Mexico | 6/10/11 | 2 | 2 |
| United States | Oklahoma | 5/15/09 | 1 | 1 |
| United States | Oklahoma | 5/17/09 | 1 | 1 |
| United States | Texas | 5/2/09 | 1 | 1 |
| United States | Texas | 5/21/09 | 1 | 1 |
| United States | Texas | 5/23/09 | 1 | 1 |
| United States | Texas | 6/12/11 | 1 | 1 |
| United States | Texas | 6/15/11 | 1 | 1 |
| United States | Texas | 8/20/11 | 1 | 1 |
| United States | Texas | 8/27/11 | 1 | 1 |
|  |  | Total | 185 | 402 |
