# Supplementary material for: Range and Frequency of Africanized Honey Bees in California (USA)
Source: PLoS One. 2015 Sep 11;10(9):e0137407. doi: 10.1371/journal.pone.0137407 (PMC4567290; doi:10.1371/journal.pone.0137407)

**Figure S1.** Maximum likelihood consensus tree of COI-COII spacer region sequences from 48 honey bee workers collected in San Diego County. Sequences labeled A, M, Y, and O are from Franck et al. (2001) and are used as representatives of the mitotype groups defined therein. All San Diego sequences are labeled (SD).


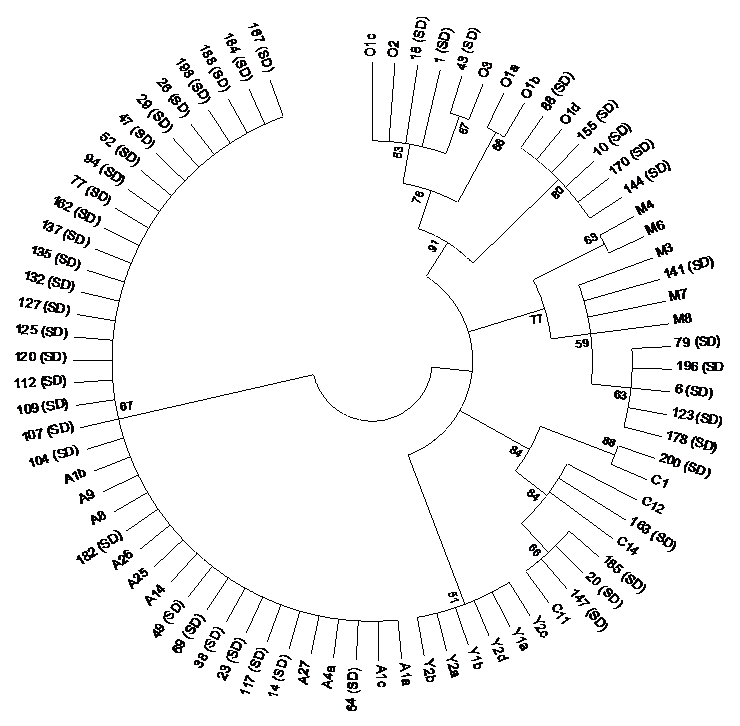

Supplement: S1 Fig — Sequences from 48 honey bee workers collected in San Diego County (this study). Sequences labeled A, M, Y, and O are from Franck et al. (2001) and are used as representatives of the mitotype groups defined therein. All San Diego sequences are labeled (SD). (DOCX) [file pone.0137407.s006.docx]
